# Supplementary material for: Neuron-specific Agrin splicing by Nova RNA-binding proteins regulates conserved neuromuscular junction development in chordates
Source: PLoS Biol. 2025 Sep 12;23(9):e3003392. doi: 10.1371/journal.pbio.3003392 (PMC12445529; doi:10.1371/journal.pbio.3003392)
Supplement: S1 Fig — Right: AlphaFold prediction of rat Agrin-Lrp4 interaction. (B) Alignment of rat and C. robusta (“Cirobu”) Lrp4 sequences highlighting the residues important for N-X-I/V/F binding as color-coded in the ribbon diagrams. (PDF) [file pbio.3003392.s001.pdf]

A

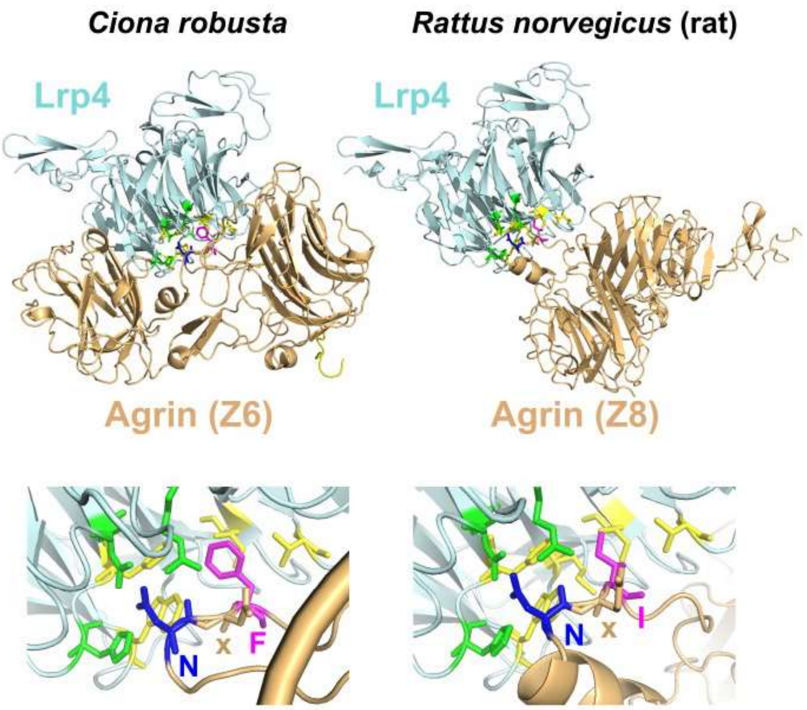

B

```
Rat          AIALDFHHRRELVFWSDVTLDRILRANLNGSNVEEVVSTGLESPGGLAVDWVHDKLYWTD
Cirobu       AIGLDYHIDKGLMFWTDVTLDRIMRSYVNGSDVQEVVSSGLESPGGVAVDWIHNLLYWTD
             **.**:*   : *:**:*******:*:  :***:*:****:*******:****:*:  *****

Rat          SGTSRIEVANLDGAHRKVLLWQSLEKPRAIALHPMEGTIYWTDWGNTPRIEASSMDGSGR
Cirobu       SGTSRLEVSLLDGSLRKVLVWERLEKPRALALHPLHSLMFWTDWGDTPRLESAAMDGSQR
             *****:**: ***: ****:*: ******:****:..  ::*****:***:*:::**** *

Rat          RIIADTHLFWPNGLTIDYAGRRMYWVDAKHHVIERANLDGSHRKAVISQGLPHPFAITVF
Cirobu       HTVASRNLHWPNGLAIDYTRNHIYWADAKYHIIEKANLDGGDRRAVISRGLLHPFGVTIF
             : :*. :*.*****:***: .::**.***:*:**:*****..*:****:** ***.:*:*

Rat          EDSLYWTDWHTKSINSANKFTGKNQEIIRNKLHFPMDIHTLHPQRQPAGKNRCGDNNGGC
Cirobu       EDKLYWTDWQTKSVNSANKFTGRGVKTIRSRLHFPMDIQTYHPLRQPNGHNRCEQ--AHC
             **.******:***:********:. : **.:*******:* ** *** *:*** :  . *
```

X Forms hydrogen bonds with N in Agrin Z loop
X Hydrophobic pocket for I/V (and possibly F) in Agrin Z loop

Figure S1. A) Left: AlphaFold prediction suggesting the N-X-F motif of the *C. robusta* Agrin Z6 loop (bottom panel) might bind to the conserved residues in Lrp4 (see panel **B)**, similar to what was previously determined for N-X-I in rat Agrin by X-ray crystallography (Zong et al. 2012). Right: AlphaFold prediction of rat Agrin-Lrp4 interaction. B) Alignment of rat and *C. robusta* ("Cirobu") Lrp4 sequences highlighting the residues important for N-X-I/V/F binding as color-coded in the ribbon diagrams.
