# Supplementary material for: Neuron-specific Agrin splicing by Nova RNA-binding proteins regulates conserved neuromuscular junction development in chordates
Source: PLoS Biol. 2025 Sep 12;23(9):e3003392. doi: 10.1371/journal.pbio.3003392 (PMC12445529; doi:10.1371/journal.pbio.3003392)
Supplement: S2 Fig — (B) Annotated B. lanceolatum (amphioxus) putative Agrin Z exon region. Based on alignment of Augustus Gene Prediction model g13824.t1 on UCSC browser. (PDF) [file pbio.3003392.s002.pdf]

Figure S2.

A) *Agrin* Z exon alignment

```
Caeleg RQHENDARGIHINEQDALVFPNNVT-------------------FSVGSSKSSNFSFDFRTLKQFGVIWQEG
Stpurp VVVINTDDPLRFDGQTSIQYYNGVSKK-------------------QRALRTHQVQLSFKTAEPNGALFWNG
Brlanc DPAPLRQEGVGLDGTTILNYPNAINKR-ESNSIREQ----------EPNQDDNHFEVTFRTTSDHGLLLWNH
Cirobu LLQDEQATAIYLDGTTKIMYRNAVKAM--PNDFRE--QLSDN----SRARTHNNYEIVFRTTARHGLLLMVG
Cisavi ILSDEQATAVYFDGSTRVMYRNAIKAI--TNEVPE--QYNDN----SRSGSHNKYELVFRTKARHGLLLIAG
Mumusc EKSVGDLETLAFDGRTYIEYLNAVTESELTNEIPAPETLDSRALFSEKALQSNHFELSLRTEATQGLVLWIG
Hosapi EKSAGDVDTLAFDGRTFVEYLNAVTESELANEIPVPETLDSGALHSEKALQSNHFELSLRTEATQGLVLWSG
                : ::    : : * :                             : .. ::*    * :
```

Color legend: **constitutive exon 1** **constitutive exon 2** **Z exon 1** **Z exon 2**
Z exon-encoded NxI/V/F motif **conserved constitutive NxI/V motif of unknown function**

Caeleg: *Caenorhabditis elegans* (nematode), Stpurp: *Strongylocentrotus purpuratus* (sea urchin), Brlanc: *Branchiostoma lanceolatum* (amphioxus), Cirobu: *Ciona robusta*, Cisavi: *Ciona savignyi*, Mumusc: *Mus musculus* (mouse), Hosapi: *Homo sapiens* (human)

B) Annotated B. lanceolatum (amphioxus) putative *Agrin* Z exon region.

Based on alignment of Augustus Gene Prediction model g13824.t1 on UCSC browser.

...CAAGCCGACCCCGCCCCCCTGCGACAGGAAGGCGTAGGCCTTGACGGGACGACCATCCTCAACTATCCCAACGCCATCAACAAAAGGTTGGTGTCTTCACAATA
CTTCATACTCTTACACTTGTCTACAACAACTTTGTGGCTTCCTTCTTTGTCTTGGGCTTCAAGAGCCTTGTGCAAAAGCTGACAAACTATAAGGAGAAATCCTTTTC
CTACTAGAATCGTGTAATTGTGAATGTAAATGAAATACTGTAGTCTGATTCTTGTTGTCTTTCCCAGAACCAGAACTTGATGAGTACAATGTGATGTAACTCTGTTC
TGCTTAATTGTAAAAAGACTGTGGAGCTTCTATTGTTTTTTCCAACATTGCACCATGCATGTTACACCCTCCCTGAGCCCTGTGCCTTGTGTGTAGTACGTGTCTAA
ACTTGTTCACCTTCTCTCTCTTCCTCTCTCTGCCTTTGTCTTCAAGGGAGTCC**AATTCTATT**CGTGAACAGTAAGTCTCTTCTTCAGCTTTCTTTTTCTCATAATTTG
AAACTTCAGAGCTTCTTTTTCCTGTTCTGATGTACAGTATTTAAGCTGACTTGTGCTTCTGTCCTGAATATGCTTTGGTCACATTTGCACTGGTGCCCATTGGGGGT
GTAACCCCGGCCACAAATTTGTCCCAGTGGACTGTTGGAGATCGGGGGTACCTCTCGGTCTTCTCACGATTAGCTTATGGCATTTTTTTTTTGCAAAGTAACAAATA
ACAGCCTGTAATCTACCCGGTTGTGCCCCTTTTTCCAATTGTGACCGTAGCAATAGATTCAGTTGTAGTCCACAGAAGCTCAGATAGTGCAATCAACCAGTTTTGTG
TACGGTTCGTTTATAGTTTGTGCACAATGATGTTGCTACTCCGACTTGCTTGTGTCTCAACAGCTGATTTGTAAAAGTACTTGTCCATCATTCTATTAGTATTTGTA
TTTTATAAGCTGATGCAGTATGTTAGATTGTCTTCTTATACATAAGACAAGATGTATGTATGTGCATACATGTATAAGCCAAATACAACATGCATCAACAATGTTTG
AAAGGGTATGACAACATCCCCTTGGTGGTAATACAGTAAAGCAGTGTGCAGTTTCTTCAATCACTGCTAGGTGGTGCTCCAAAACATTCAAAGTAAGACCTTTCCTT
CATACTGTTCCAGTGTGTACTATAAATTAAGCTTCATGTTTGCCGATTTTCATTTTGTGTTCTGACCTTTAACCTTTAGGGAGCCCAACCAGGACGACAATCACTTT
GAGGTGACCTTCAGAACGACCTCTGACCACGGGCTGCTGCTGTGGAACCACAAACCCGGCGGCGGGGACTTCATCGCCCTTGCCATC...

Color legend:
constitutive exons splice donor site splice acceptor site putative Z-exon **encodes NxI/V motif**
