# Supplementary material for: Neuron-specific Agrin splicing by Nova RNA-binding proteins regulates conserved neuromuscular junction development in chordates
Source: PLoS Biol. 2025 Sep 12;23(9):e3003392. doi: 10.1371/journal.pbio.3003392 (PMC12445529; doi:10.1371/journal.pbio.3003392)
Supplement: S3 Fig — Based on alignment of RefSeq Gene agr-1 on UCSC genome browser. (B) S. purpuratus Agrin intron where Z exons should have been. Based on alignment of mRNA JT097480 on UCSC genome browser. (PDF) [file pbio.3003392.s003.pdf]

Figure S3.

A) ***C. elegans Agrin*** **intron where Z exons should have been.**
Based on alignment of RefSeq Gene *agr-1* on UCSC genome browser.

```
agATGATGCTCGAGGAATTCACATCAACGAGCAAGATGCTCTCGTTTTTCCAAACAATGTCACTTTCTCgtgagtcattttctataaca
aatgttataatttttctaacttttctcataaacaattcaccttcactacgagttataaacaatattgtttcgtaaaaattcatattt
tatttttcagTGTTGGTTCGTCCAAGAGTTCAAATTTTTCATTCGACTTCCGCACCTTAAAACAATTTGgt
```

exon 26 exon 27 splice donor site splice acceptor site

B) ***S. purpuratus Agrin*** **intron where Z exons should have been.**
Based on alignment of mRNA JT097480 on UCSC genome browser.

```
agAAATGCCTGTAACAACCCCCAGACCACCTGTGGTTGCCACGAGAGCAAGACCTGCCATGACGACTGCATCTGTAGTGGTTATTAATA
CTGATGATCCCTTGAGGTTTGATGGACAGACAAGTATTCAGTACTACAATGGAGTTTCCAAAAAgtgagtctcttttgttgttgatgt
tttgatgtcatgttatcaaactcatgtacaatttgatcttttctgatttaggaattatatttttcaaaggtcagaatgacaaataatt
caatcaattttttgttgaaccttctgtccacgtcttgctttctctaaagatttaatttatactggaattaaatttgtcctttggaggtgt
gttcaaattgcttttgtcaccccggaccagcagactgtatccttctctctctcctctctactttcctataactgtctgcatgtattatg
aaatttctatgtattatctgattgttatactcattgtatgtttttttgattggtcaaataaatattaataataaaaaataatattaatct
tgacaccaaacttctttggtgcaagcaattgtttttattgttctttaaaaaaaaaagcaattgtaagaagtttagttagaactgcagtt
aatctaaaggtaatttttcttttttccttcggaaagtctgaatagtattgatctaactgacattctaagacatatgttatgtgatccgtcc
catttataagaaatggagcatgcttttactgtagttttaaaagatttgacctaccctaaaccatatcttacaccacacaatcatttcta
ctttgcagACAAAGAGCTCTACGGACCCACCAAATCCAGCTCTCTTTCAAAACAGCCGAACCAAATGGCGCCCTCTTTTGGAACGGAGT
AGGCAATGCTGACTTCCAGGCTGTAGGAGTCTCAGATGGCTATGTAGAATATGCATACAATCTAGGAAGGGGTATTACAAGGATAAGAA
CTACGCAGAAGGTTGATAATAACAAATGGCATACTGTTATTATAACAAGgt
```

exon 10 exon 11 splice donor site splice acceptor site
