# Supplementary material for: Neuron-specific Agrin splicing by Nova RNA-binding proteins regulates conserved neuromuscular junction development in chordates
Source: PLoS Biol. 2025 Sep 12;23(9):e3003392. doi: 10.1371/journal.pbio.3003392 (PMC12445529; doi:10.1371/journal.pbio.3003392)
Supplement: S4 Fig — M: DNA molecular weight marker in kilobase pairs. (PDF) [file pbio.3003392.s004.pdf]

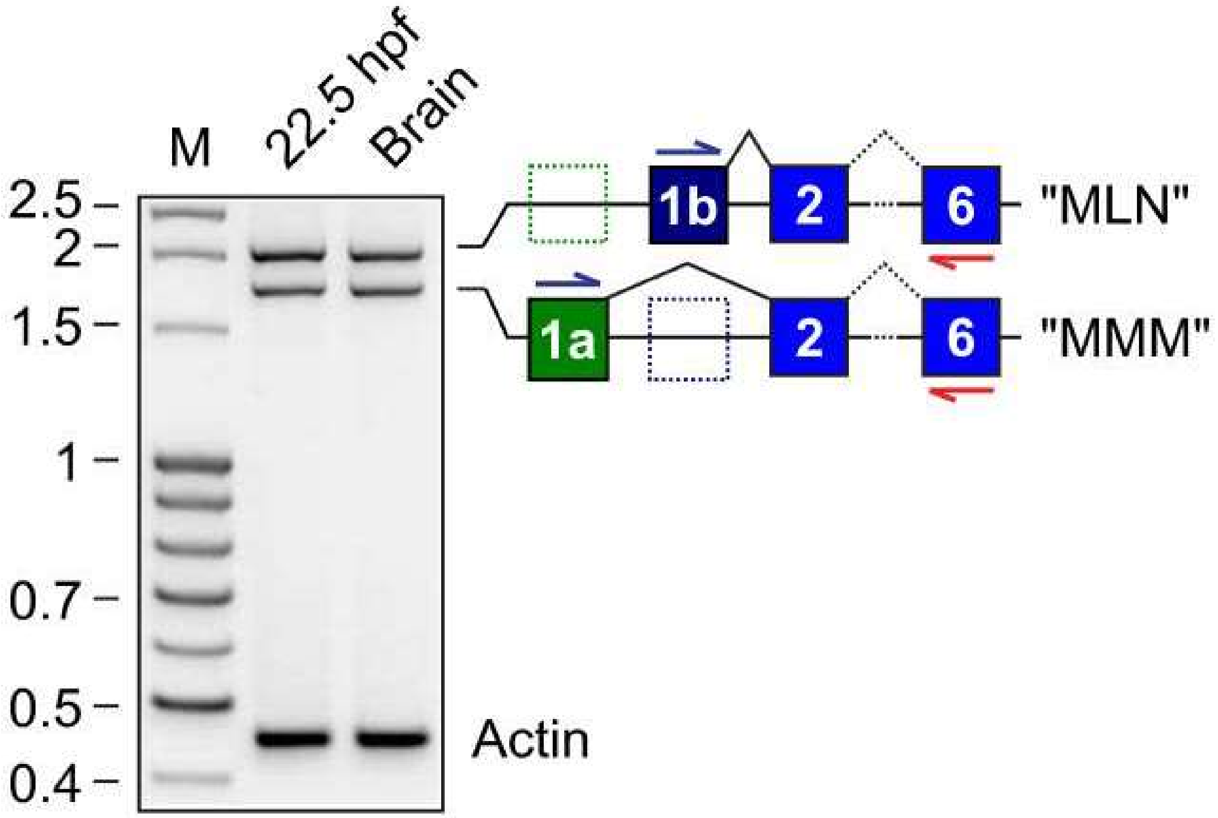

Figure S4. RT-PCR detection of different *Nova* alternative splice forms in larvae (22.5 hours post-fertilization) and adult brain.

M: DNA molecular weight marker in kilobase pairs.
