# Supplementary material for: Neuron-specific Agrin splicing by Nova RNA-binding proteins regulates conserved neuromuscular junction development in chordates
Source: PLoS Biol. 2025 Sep 12;23(9):e3003392. doi: 10.1371/journal.pbio.3003392 (PMC12445529; doi:10.1371/journal.pbio.3003392)
Supplement: S5 Fig — Left: annotated cell clusters (CNS: central nervous system, PGCs: primordial germ cells), right: Nova expression mapped onto cell clusters, showing enrichment in a subset of posterior mesenchyme cells that also express Hlx (Imai and colleagues (2004), Cao and colleagues (2019)), and in the CNS, as confirmed by in situ hybridization (see Fig 2). (B) Nova mRNA in situ hybridization coupled to immunostaining-based detection of Fgf8/17/18 > H2B::mCherry expression, revealing identity of Nova+ MN2 cells adjacent to the Fgf8/17/18 reporter-expressing cells. (PDF) [file pbio.3003392.s005.pdf]

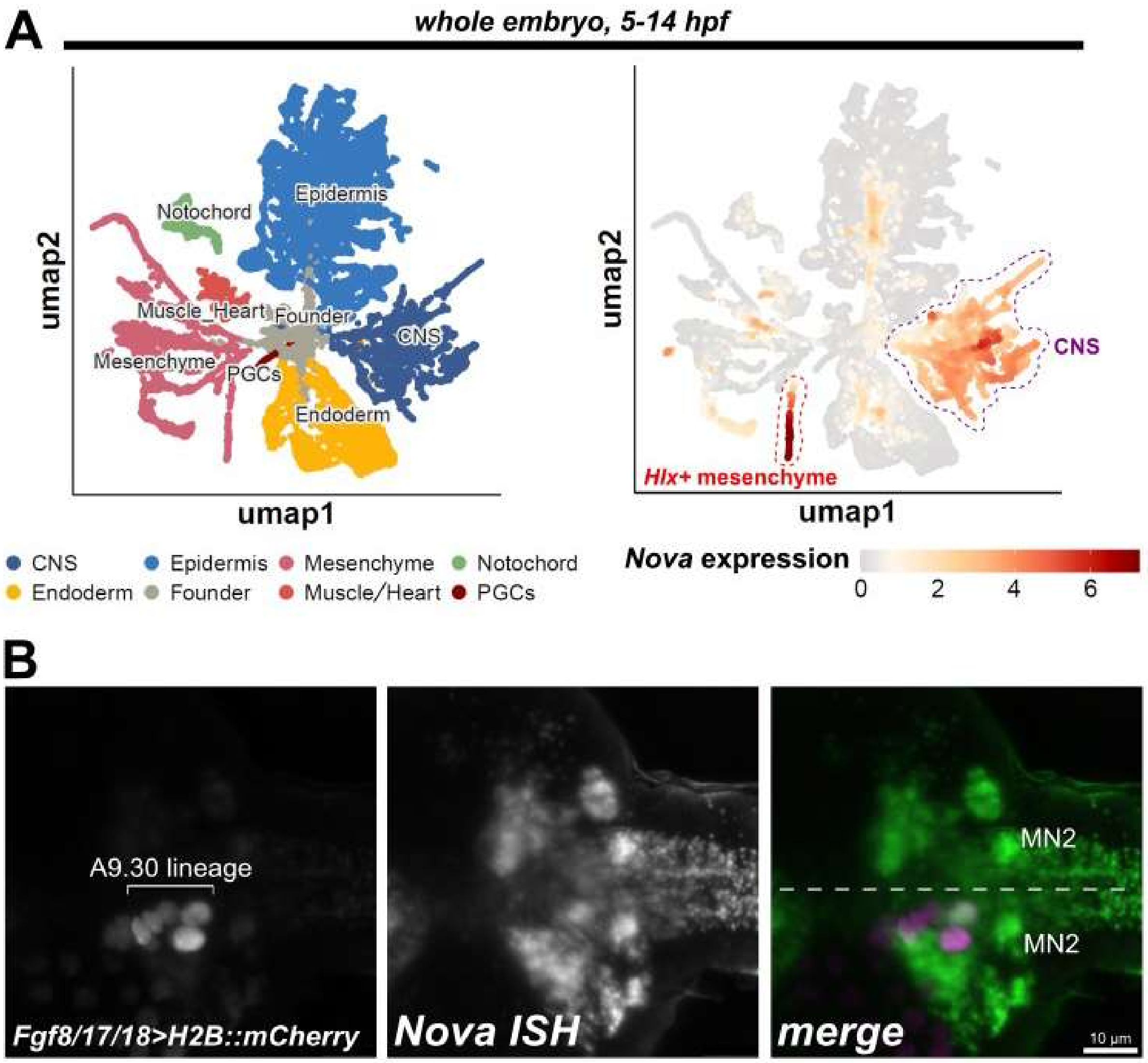

Figure S5. Additional *Nova* expression profiling

A) UMAP plots showing re-analysis of *Nova* expression from recent single-cell RNAseq profiling of pooled cells from whole embryos collected at hourly time points spanning 5 to 14 hpf at 18°C (underlying data can be found in Bernadskaya et al. 2024). Left: annotated cell clusters (CNS: central nervous system, PGCs: primordial germ cells), right: *Nova* expression mapped onto cell clusters, showing enrichment in a subset of posterior mesenchyme cells that also express *Hlx* (Imai et al. 2004, Cao et al. 2019), and in the CNS, as confirmed by *in situ* hybridization (see **Figure 2**). **B**) *Nova* mRNA *in situ* hybridization coupled to immunostaining-based detection of Fgf8/17/18>H2B::mCherry expression, revealing identity of *Nova*+ MN2 cells adjacent to the *Fgf8/17/18* reporter-expressing cells.
