# Supplementary material for: Neuron-specific Agrin splicing by Nova RNA-binding proteins regulates conserved neuromuscular junction development in chordates
Source: PLoS Biol. 2025 Sep 12;23(9):e3003392. doi: 10.1371/journal.pbio.3003392 (PMC12445529; doi:10.1371/journal.pbio.3003392)
Supplement: S6 Fig — All Nova proteins were fused to GFP (“enhanced GFP”, or EGFP) at the N-terminus. Anti-GFP primary antibody was used to detect GFP::Nova isoform expression. Untagged GFP expression was monitored as a positive control. Red bands: molecular weight marker in kilodaltons (KD). See materials and methods for details. (B) Both “MMM” and “MLN” isoforms of Ciona Nova can splice Ciona Agrin minigenes in mammalian cells. “MEY” isoform is the same as “MLN” but using a hypothetical alternate start codon four amino acid residues after the MLN start codon. This was tested even though there is no evidence to suggest the MEY isoform is a naturally occurring one. M: DNA molecular weight marker in kilobase pairs. H2O: using water instead of cDNA template for PCR. −RT: no reverse transcriptase added. (PDF) [file pbio.3003392.s006.pdf]

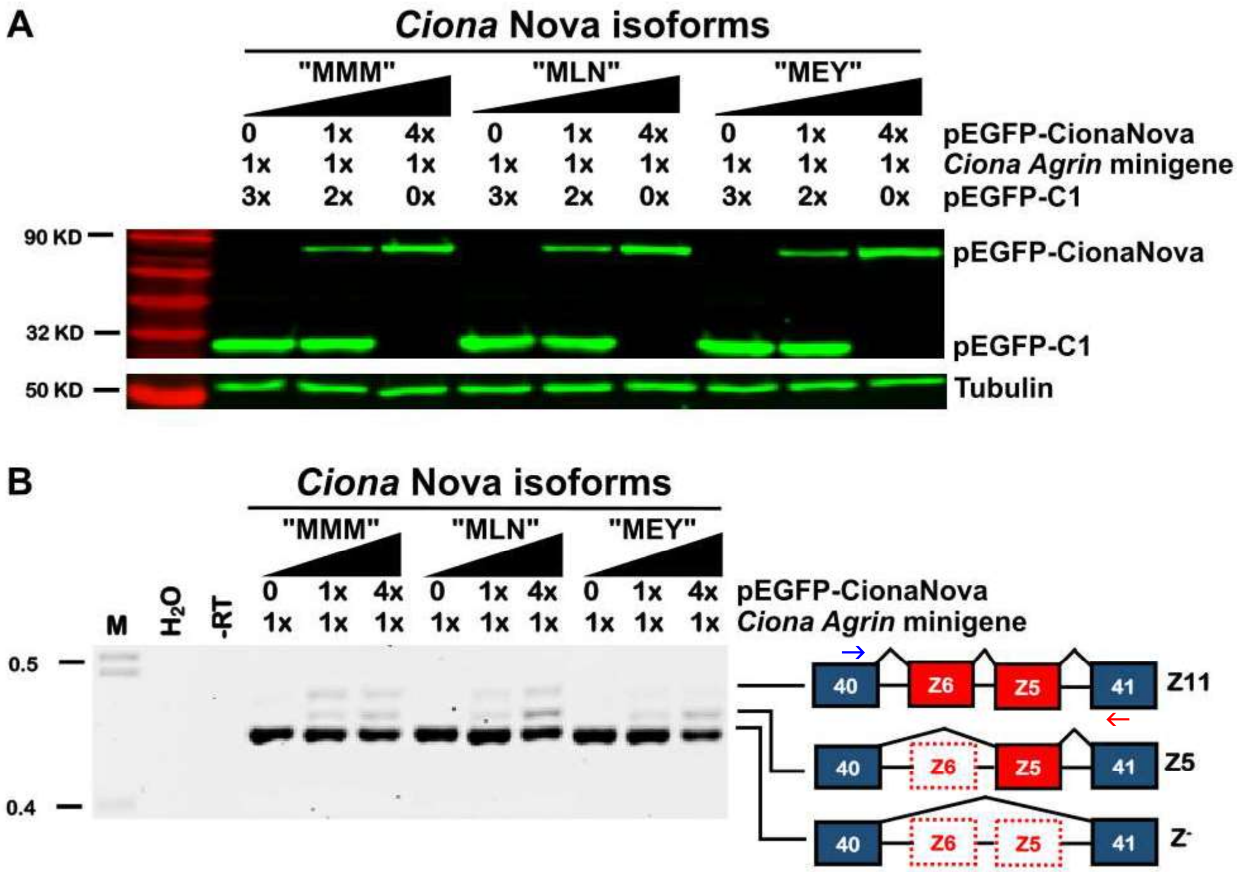

Figure S6. A) Western blot indicating comparable expression of all three *Ciona* Nova isoforms tested, in mammalian cells. All Nova proteins were fused to GFP ("enhanced GFP", or EGFP) at the N-terminus. Anti-GFP primary antibody was used to detect GFP::Nova isoform expression. Untagged GFP expression was monitored as a positive control. Red bands: molecular weight marker in kilodaltons (KD). See materials and methods for details. **B)** Both "MMM" and "MLN" isoforms of *Ciona* Nova can splice *Ciona Agrin* minigenes in mammalian cells. "MEY" isoform is the same as "MLN" but using a hypothetical alternate start codon four amino acid residues after the MLN start codon. This was tested even though there is no evidence to suggest the MEY isoform is a naturally occurring one. M: DNA molecular weight marker in kilobase pairs. H_2O: using water instead of cDNA template for PCR. -RT: no reverse transcriptase added.
