# Supplementary material for: Neuron-specific Agrin splicing by Nova RNA-binding proteins regulates conserved neuromuscular junction development in chordates
Source: PLoS Biol. 2025 Sep 12;23(9):e3003392. doi: 10.1371/journal.pbio.3003392 (PMC12445529; doi:10.1371/journal.pbio.3003392)
Supplement: S7 Fig — All Nova proteins were fused to GFP (“enhanced GFP”, or EGFP) at the N-terminus. Different Ciona Nova isoforms (“MLN”, “MMM”, “MEY”) differed by their N-termini, thus N-terminal deletions (ΔNter) were identical regardless of isoform. (PDF) [file pbio.3003392.s007.pdf]

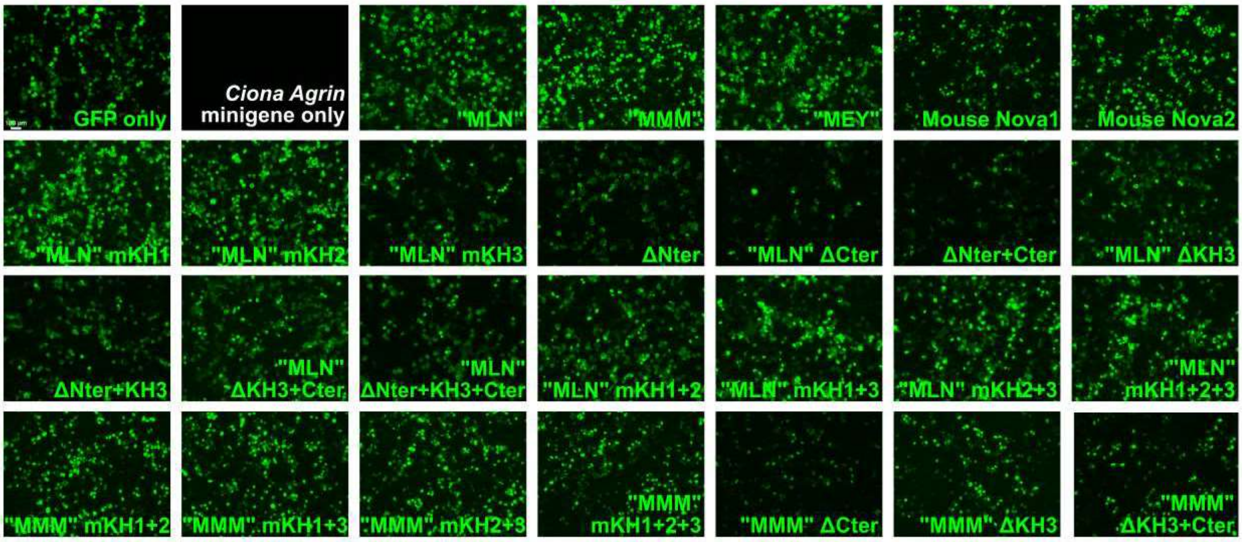

Figure S7. GFP fluorescence images of transfected mammalian cells to monitor *Ciona* and mouse Nova expression and localization in the minigene assays. All Nova proteins were fused to GFP ("enhanced GFP", or EGFP) at the N-terminus. Different *Ciona* Nova isoforms ("MLN", "MMM", "MEY") differed by their N-termini, thus N-terminal deletions (ΔNter) were identical regardless of isoform.
