# Supplementary material for: Neuron-specific Agrin splicing by Nova RNA-binding proteins regulates conserved neuromuscular junction development in chordates
Source: PLoS Biol. 2025 Sep 12;23(9):e3003392. doi: 10.1371/journal.pbio.3003392 (PMC12445529; doi:10.1371/journal.pbio.3003392)
Supplement: S8 Fig — (B) Same minigene assay using different combinations of GDDG mutant KH domains in Ciona Nova “MLN” isoform. (C) Replicate of minigene assay using different Ciona Nova (MLN) deletion mutants (see Fig 3). M: DNA molecular weight marker in kilobase pairs. H2O: using water instead of cDNA template for PCR. no RT: no reverse transcriptase added. (PDF) [file pbio.3003392.s008.pdf]

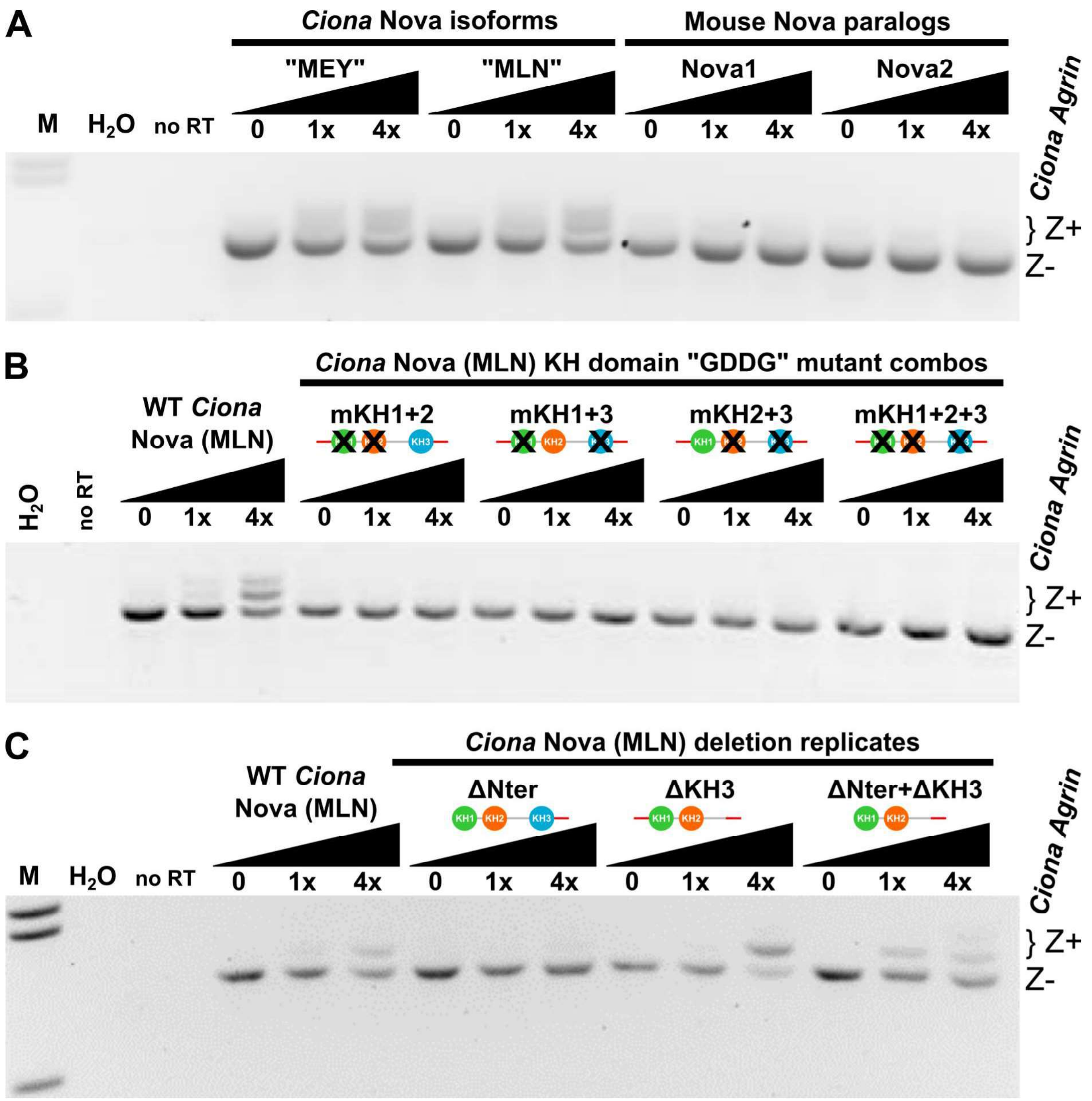

Figure S8. A) Replicate data showing repeated *Ciona Agrin* minigene Z exon inclusion by *Ciona* Nova proteins (MEY and MLN putative isoforms), but not by mouse Nova1 or Nova2. **B)** Same minigene assay using different combinations of GDDG mutant KH domains in *Ciona* Nova "MLN" isoform. **C)** Replicate of minigene assay using different *Ciona* Nova (MLN) deletion mutants (see main figure 3). M: DNA molecular weight marker in kilobase pairs. H_2O: using water instead of cDNA template for PCR. no RT: no reverse transcriptase added.
