# Supplementary material for: Neuron-specific Agrin splicing by Nova RNA-binding proteins regulates conserved neuromuscular junction development in chordates
Source: PLoS Biol. 2025 Sep 12;23(9):e3003392. doi: 10.1371/journal.pbio.3003392 (PMC12445529; doi:10.1371/journal.pbio.3003392)
Supplement: S9 Fig — Note: all “MMM” versions lacking the N-terminus are the same constructs as for the “MLN” versions, due to the only difference between these isoforms is the N-terminus. See Supplemental Sequences file for detailed sequence information. M: DNA molecular weight marker in kilobase pairs. H2O: using water instead of cDNA template for PCR. no RT: no reverse transcriptase added. (PDF) [file pbio.3003392.s009.pdf]

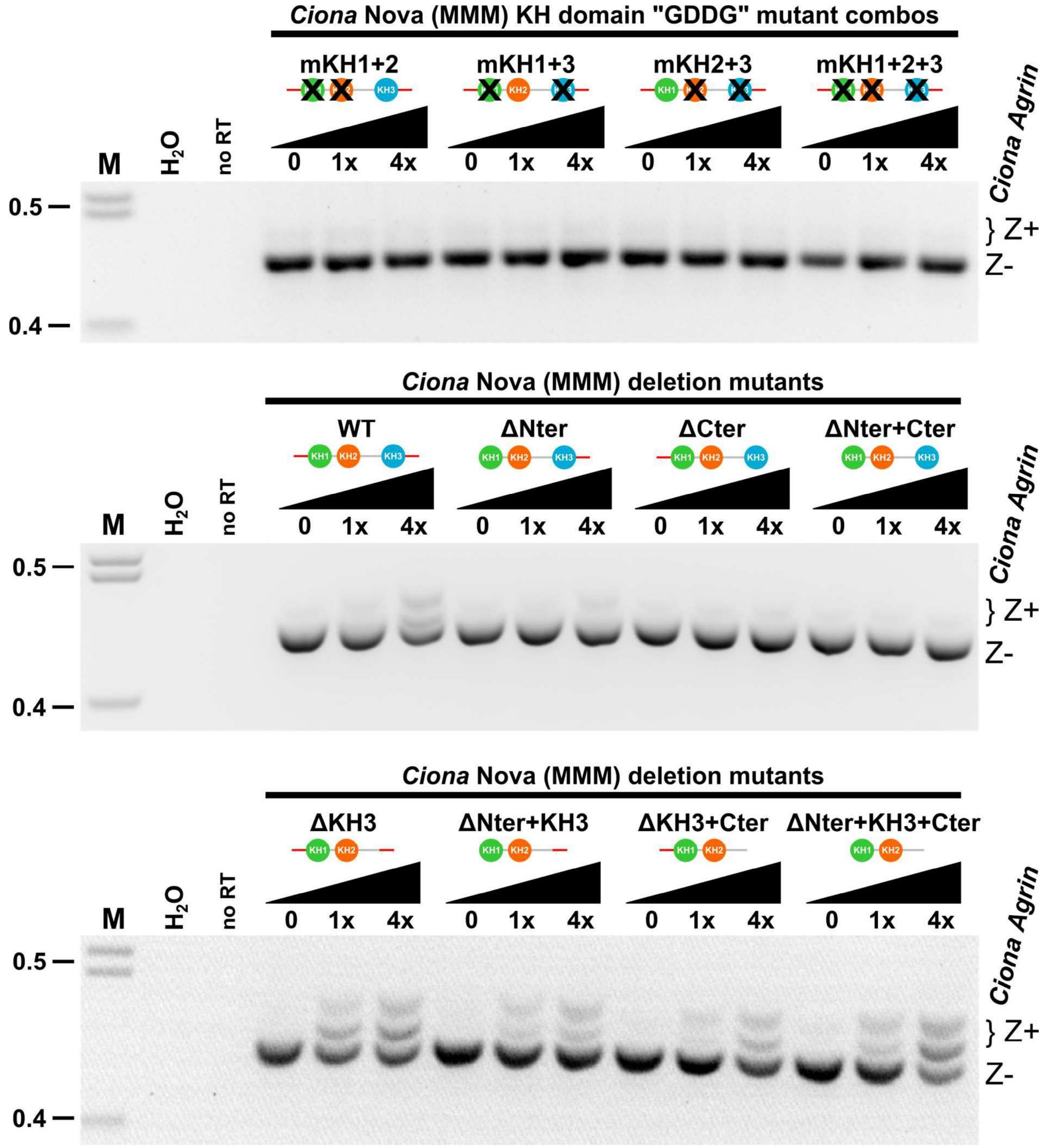

Figure S9. *Ciona Agrin* minigene assay repeated using "MMM" isoform versions of the Nova KH domain "GDDG" mutant and deletions, replicating the effects observed with the "MLN" versions (see main figures and **Figure S8**). Note: all "MMM" versions lacking the N-terminus are the same constructs as for the "MLN" versions, due to the only difference between these isoforms is the N-terminus. See **Supplemental Sequences** file for detailed sequence information. M: DNA molecular weight marker in kilobase pairs. H_2O: using water instead of cDNA template for PCR. no RT: no reverse transcriptase added.
