# Supplementary material for: Neuron-specific Agrin splicing by Nova RNA-binding proteins regulates conserved neuromuscular junction development in chordates
Source: PLoS Biol. 2025 Sep 12;23(9):e3003392. doi: 10.1371/journal.pbio.3003392 (PMC12445529; doi:10.1371/journal.pbio.3003392)
Supplement: S10 Fig — Experiment performed, assayed, and presented as in Fig 4. Smaller products seen with exonic YCAY > YAAY mutations likely represent aberrantly-running products. M: DNA molecular weight marker in kilobase pairs. H2O: using water instead of cDNA template for PCR. no RT: no reverse transcriptase added. (PDF) [file pbio.3003392.s010.pdf]

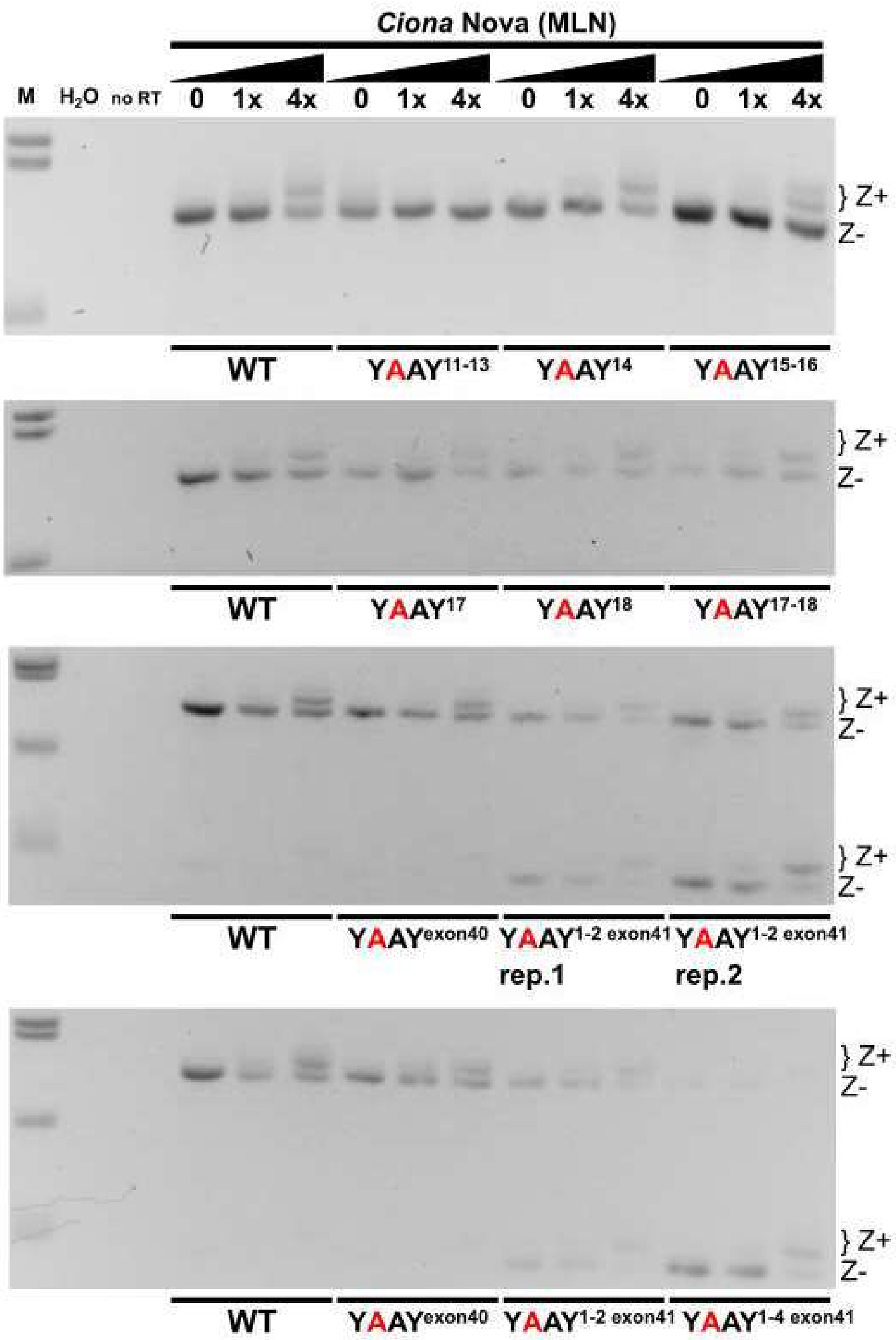

Figure S10. Additional candidate YCAY site mutagenesis experiments.
Experiment performed, assayed, and presented as in main Figure 4. Smaller products seen with exonic YCAY>YAAY mutations likely represent aberrantly-running products. M: DNA molecular weight marker in kilobase pairs. H_2O: using water instead of cDNA template for PCR. no RT: no reverse transcriptase added.
