# Supplementary material for: Neuron-specific Agrin splicing by Nova RNA-binding proteins regulates conserved neuromuscular junction development in chordates
Source: PLoS Biol. 2025 Sep 12;23(9):e3003392. doi: 10.1371/journal.pbio.3003392 (PMC12445529; doi:10.1371/journal.pbio.3003392)
Supplement: S11 Fig — Underlying data can be found in S1 Data file. (PDF) [file pbio.3003392.s011.pdf]

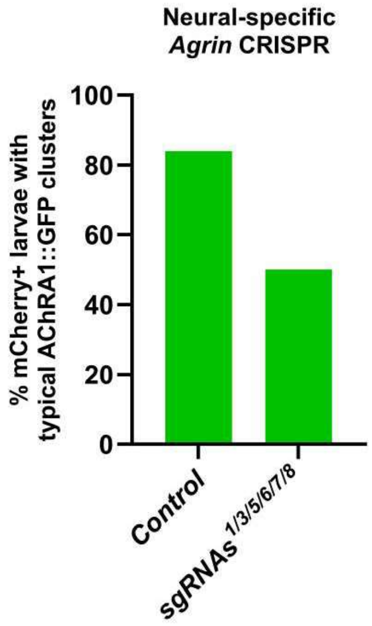

Figure S11. Scoring of AChRA1::GFP clustering upon initial neural-specific CRISPR-based disruption of *Agrin* using 6 different sgRNA expression cassettes in the form of PCR products.

Underlying data can be found in S1 Data file.
