# Supplementary material for: Neuron-specific Agrin splicing by Nova RNA-binding proteins regulates conserved neuromuscular junction development in chordates
Source: PLoS Biol. 2025 Sep 12;23(9):e3003392. doi: 10.1371/journal.pbio.3003392 (PMC12445529; doi:10.1371/journal.pbio.3003392)
Supplement: S12 Fig — Red arrows indicate indel plots showing estimated sgRNA efficacy. Red asterisks indicate naturally-occurring indels. Agrin sgRNAs 2 and 4 were not validated due to their inclusion in the seemingly least effective sgRNA combination, combo #2 (see Fig 5). All underlying NGS data from Azenta/Genewiz can be found at https://osf.io/xdc7t/. (PDF) [file pbio.3003392.s012.pdf]

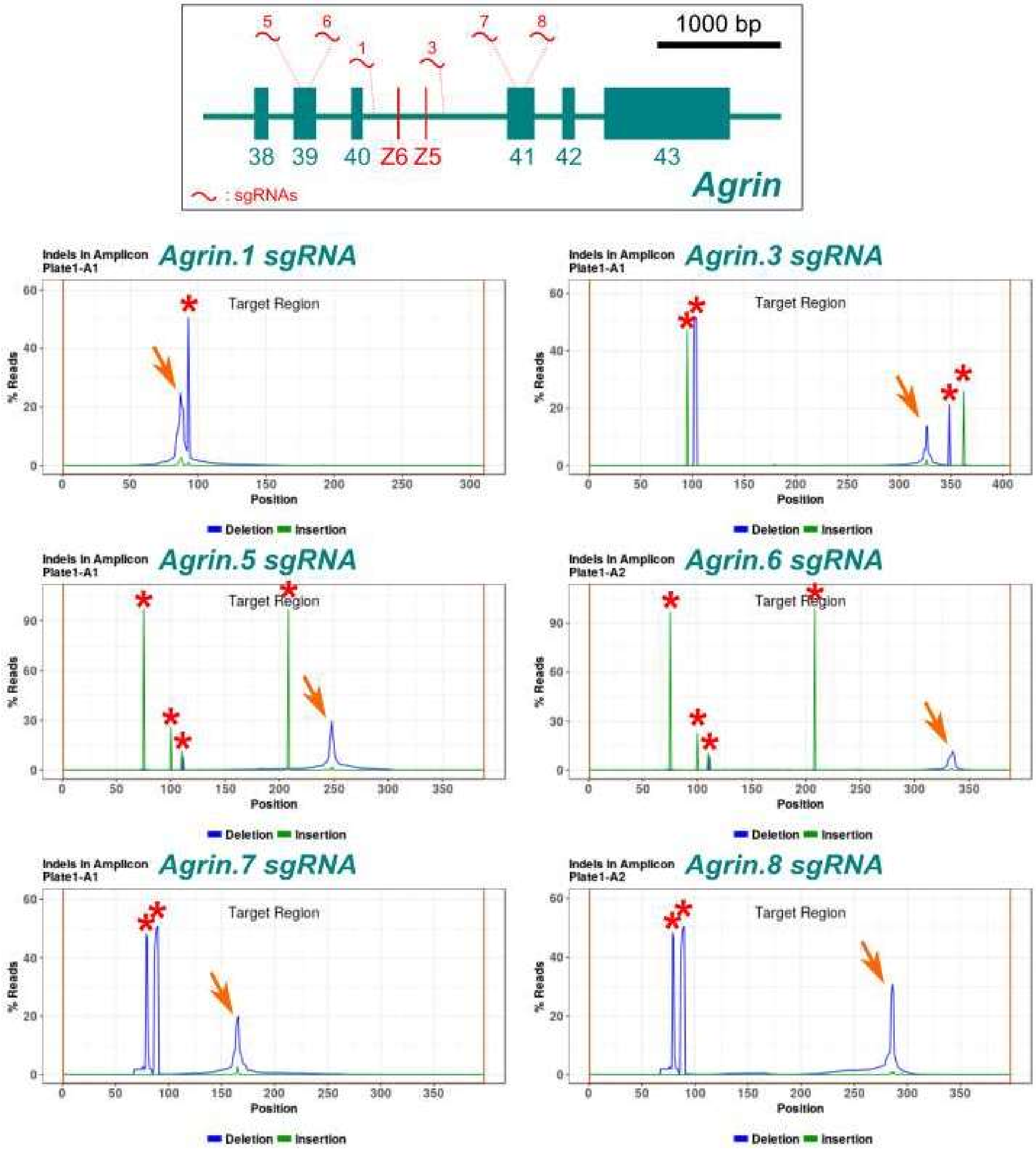

Figure S12. Illumina sequencing-based validation of selected *Agrin* sgRNAs. Red arrows indicate indel plots showing estimated sgRNA efficacy. Red asterisks indicate naturally-occurring indels. *Agrin* sgRNAs 2 and 4 were not validated due to their inclusion in the seemingly least effective sgRNA combination, combo #2 (see Figure 5). All underlying NGS data from Azenta/Genewiz can be found at https://osf.io/xdc7t/
