# Supplementary material for: Neuron-specific Agrin splicing by Nova RNA-binding proteins regulates conserved neuromuscular junction development in chordates
Source: PLoS Biol. 2025 Sep 12;23(9):e3003392. doi: 10.1371/journal.pbio.3003392 (PMC12445529; doi:10.1371/journal.pbio.3003392)
Supplement: S13 Fig — Underlying Sanger sequencing data can be found in S1 Data file. (PDF) [file pbio.3003392.s013.pdf]

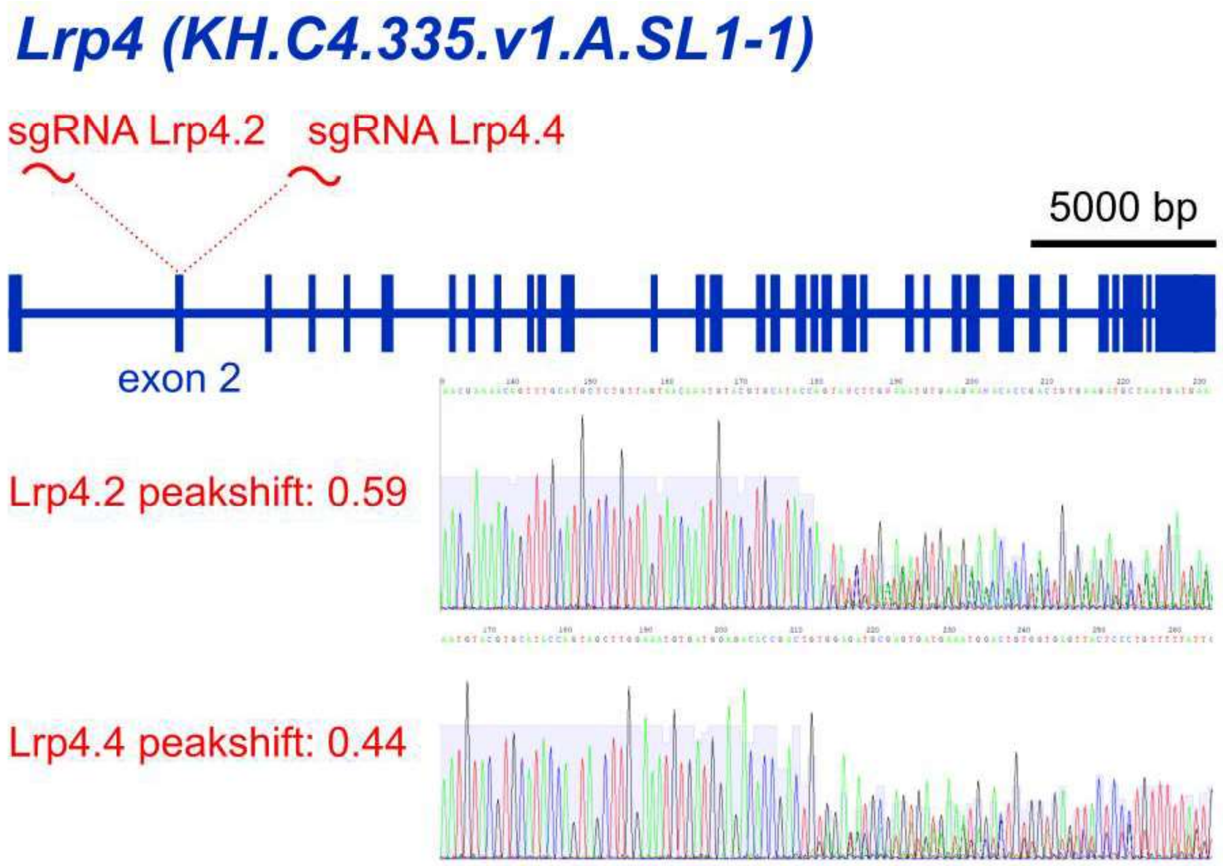

Figure S13. "Peakshift" (Sanger sequencing-based) validation of *Lrp4* targeting sgRNAs.

Underlying Sanger sequencing data can be found in S1 Data file.
