# Supplementary material for: Neuron-specific Agrin splicing by Nova RNA-binding proteins regulates conserved neuromuscular junction development in chordates
Source: PLoS Biol. 2025 Sep 12;23(9):e3003392. doi: 10.1371/journal.pbio.3003392 (PMC12445529; doi:10.1371/journal.pbio.3003392)
Supplement: S14 Fig — Underlying Sanger sequencing data can be found in S1 Data file. (PDF) [file pbio.3003392.s014.pdf]

EXON intron Nova.1.2 Nova.2.1 Nova.2.3 PAM

Exon 1b ATAATGCTAAATGCAATGGAGTATGAATGCCAGTACAATGCTGGCTACAGCATTGTGTCTAACGGTAACG
AATACGGTCTCATACAGGCCTACACGGCACACGGtgagaaactgtatattaaaagggatttaaaaacgag
acagaaacgagtgggaaatatgcagcctgtaatggaatctgacaaagcacagaatgtatctctaaaaaga
tctgaaaacccaataatatcgattctaatacaatataagctgcaatcttgtatatattaccactatgtag
tttttttcacaattttcggtttaatgacacttacgttattgttatacttgcagATTACCCCCTTGAAAAC
GGAGTGACGTTTTCAGCACCTCCGCCGGGCCAGCTCATTCTTAAAGTTCTAATACCGGGGTACGCTGCGG
Exon 2 GGGCGGTGATCGGGAAAGGCGGTCAGATTATTGTACAACTTCAGAAAGATTCAGGGGCCATTATTAAGCT
GTCAAAAGCGAAGGACTTTTACCCCG

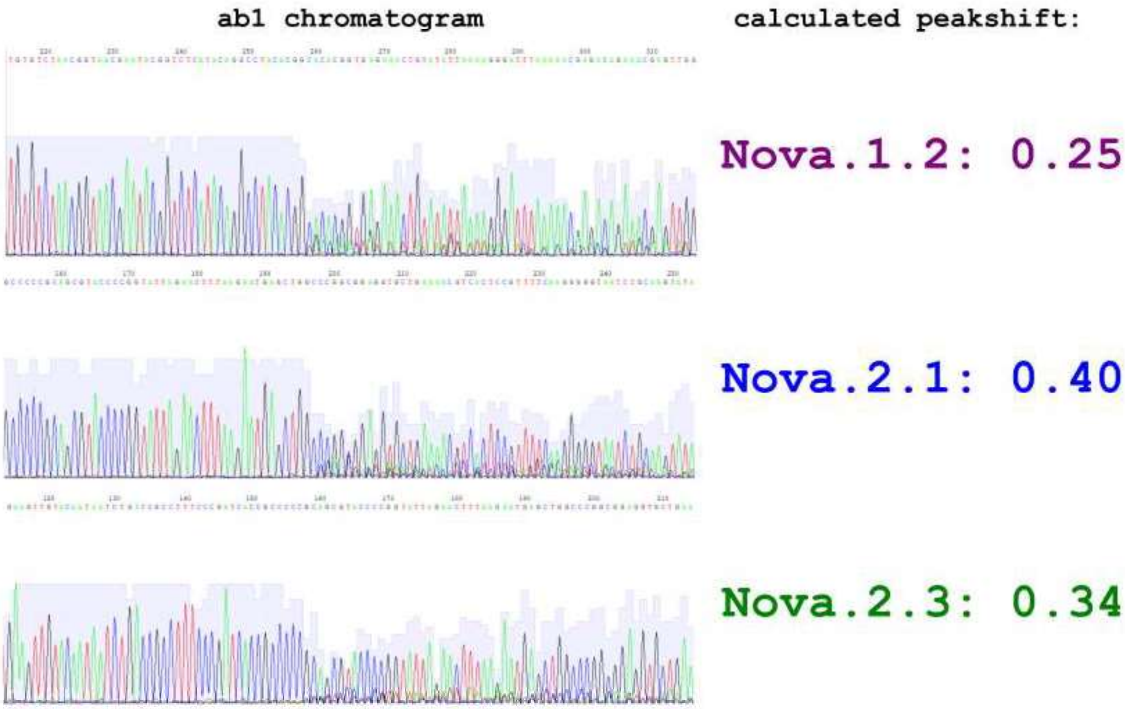

Figure S14. "Peakshift" validation of *Nova*-targeting sgRNAs.
Underlying Sanger sequencing data can be found in S1 Data file.
