# Supplementary material for: Neuron-specific Agrin splicing by Nova RNA-binding proteins regulates conserved neuromuscular junction development in chordates
Source: PLoS Biol. 2025 Sep 12;23(9):e3003392. doi: 10.1371/journal.pbio.3003392 (PMC12445529; doi:10.1371/journal.pbio.3003392)
Supplement: S15 Fig — Larva electroporated with Nova[1a] − 922/-1 > GFP, showing fluorescence in mesenchyme and epidermis, but not in neurons. (PDF) [file pbio.3003392.s015.pdf]

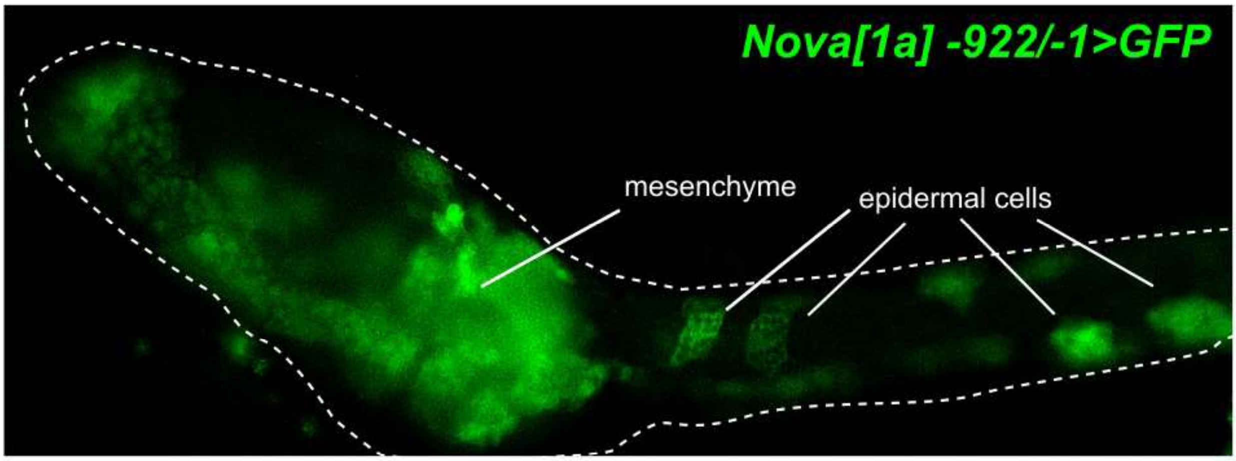

Figure S15. Smaller fragment upstream of the “MMM” isoform start (exon 1a) does not drive neuronal expression.

Larva electroporated with *Nova[1a] -922/-1>GFP,* showing fluorescence in mesenchyme and epidermis, but not in neurons.
