# Supplementary material for: Neuron-specific Agrin splicing by Nova RNA-binding proteins regulates conserved neuromuscular junction development in chordates
Source: PLoS Biol. 2025 Sep 12;23(9):e3003392. doi: 10.1371/journal.pbio.3003392 (PMC12445529; doi:10.1371/journal.pbio.3003392)
Supplement: S1 Raw Images — (PDF) [file pbio.3003392.s017.pdf]

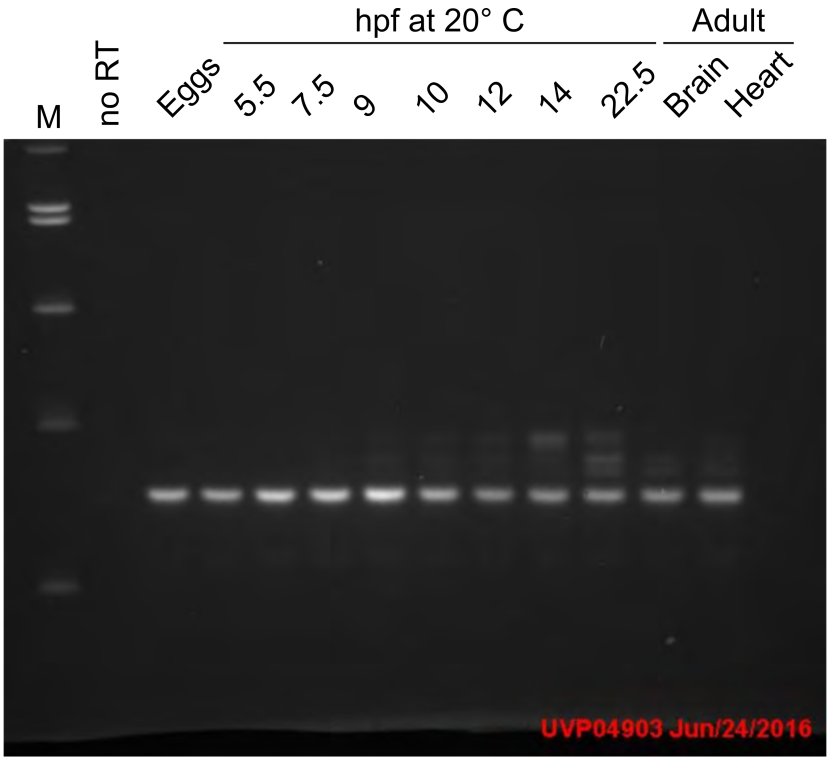

Figure 1D top panel

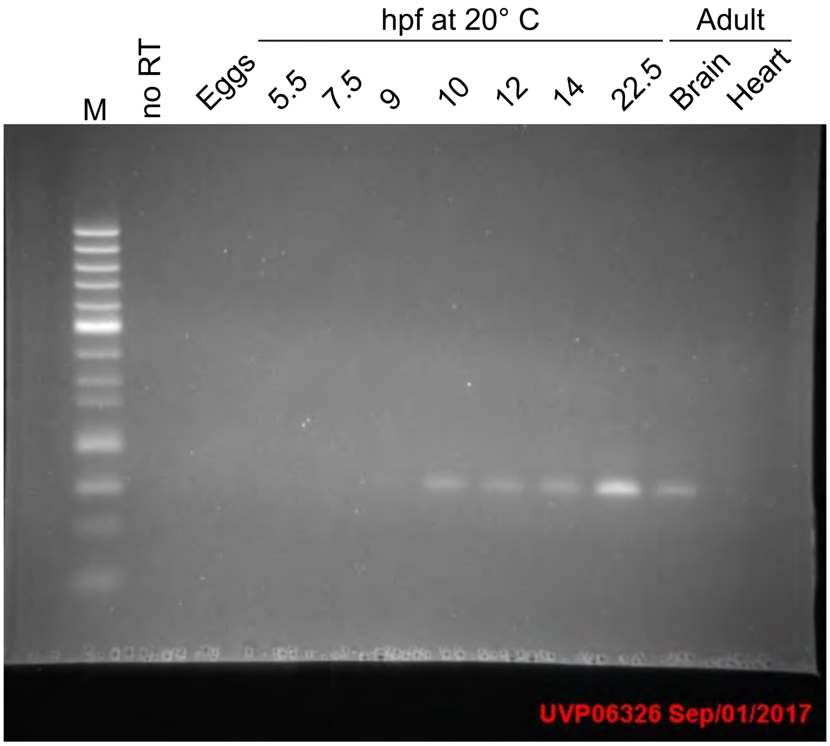

Figure 1D bottom panel

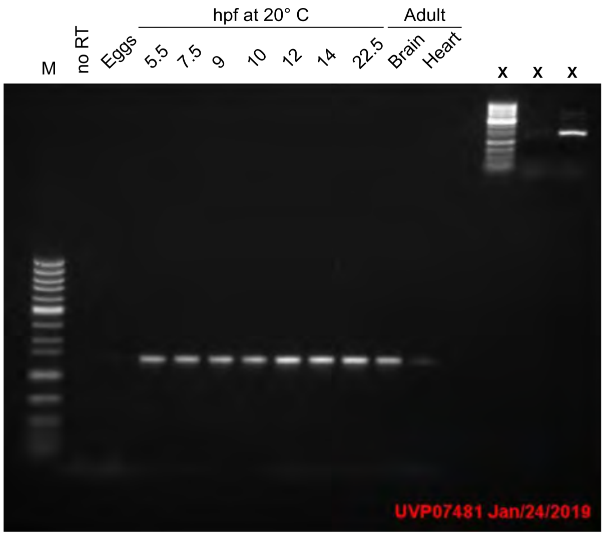

Figure 1G top panel

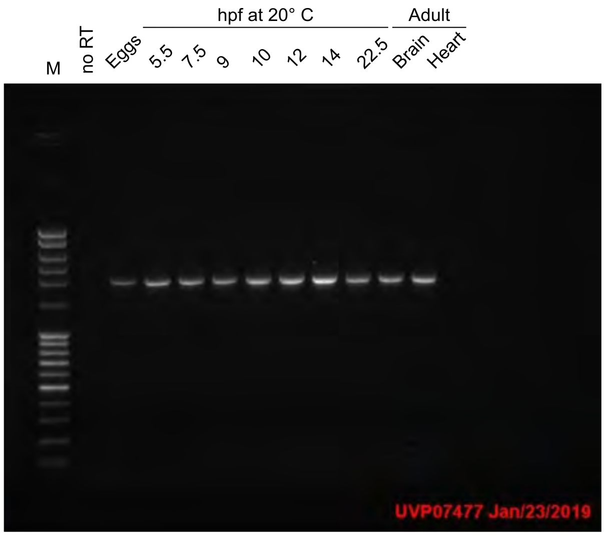

Figure 1G second panel (from top to bottom)

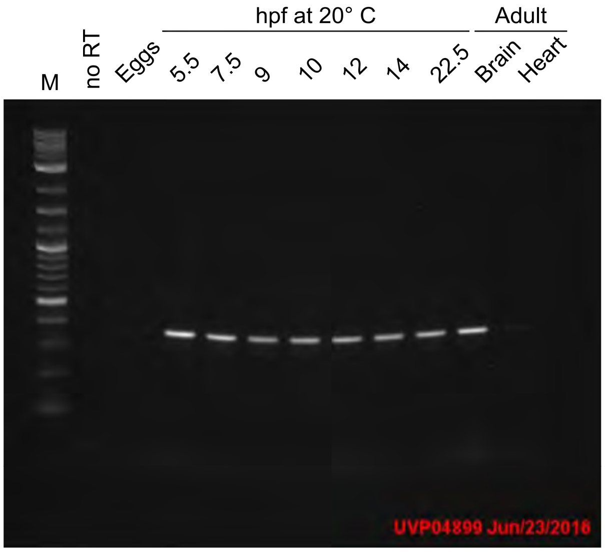

Figure 1G third panel (from top to bottom)

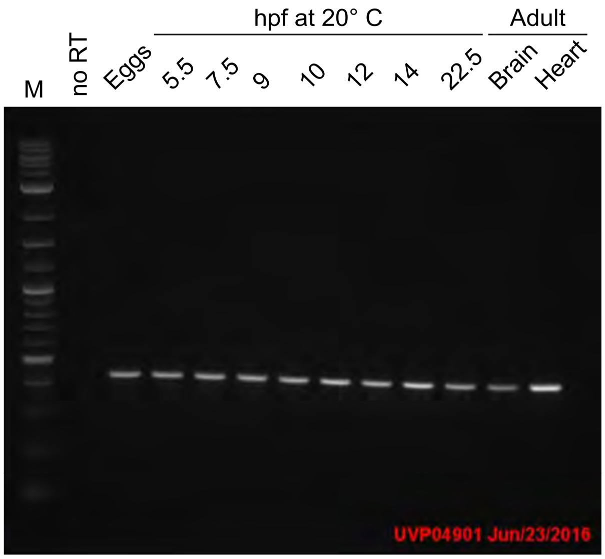

Figure 1G bottom panel

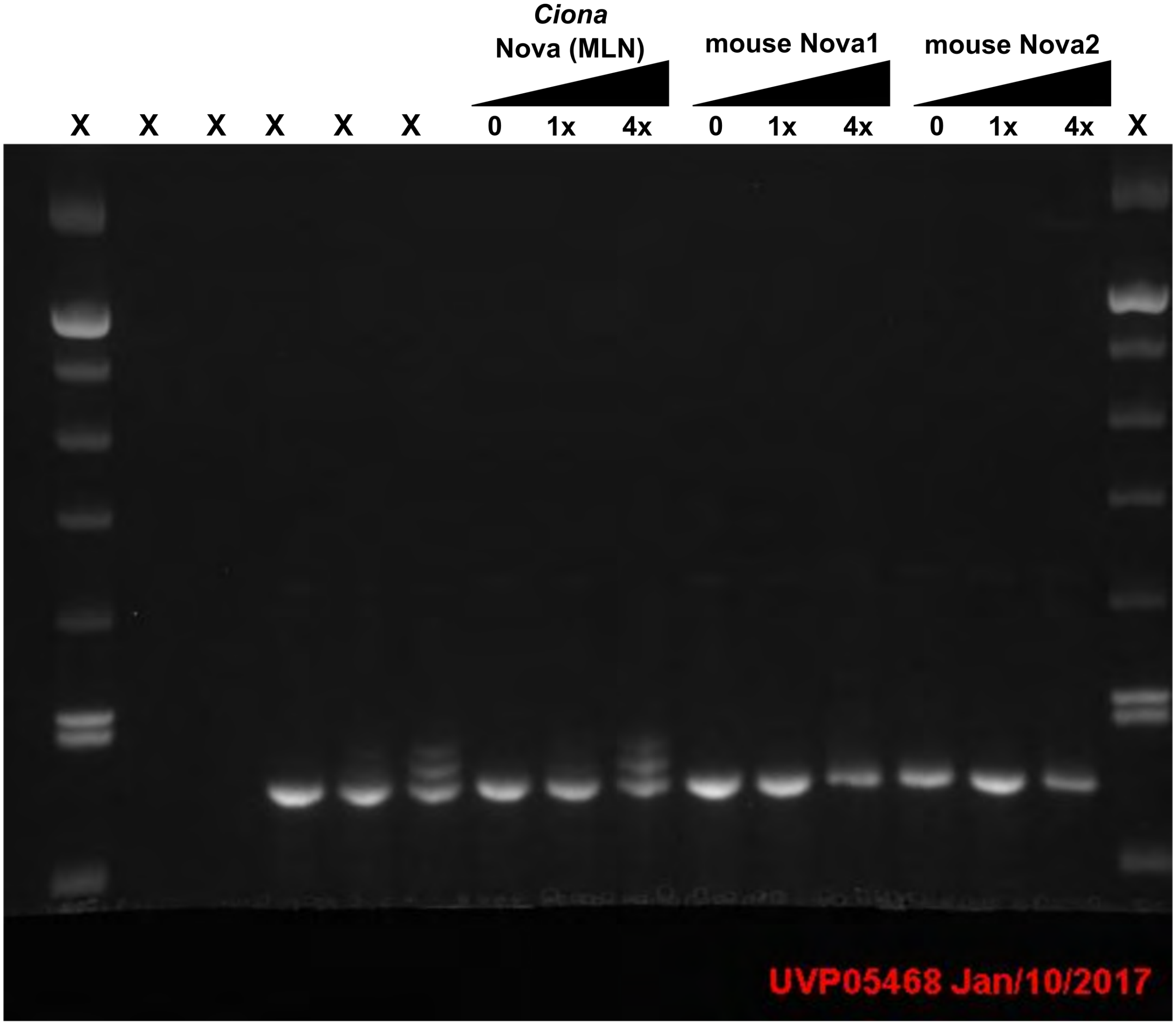

Ciona
Nova (MLN)
mouse Nova1
mouse Nova2
X X X X X X 0 1x 4x 0 1x 4x 0 1x 4x X
UVP05468 Jan/10/2017

Figure 3B

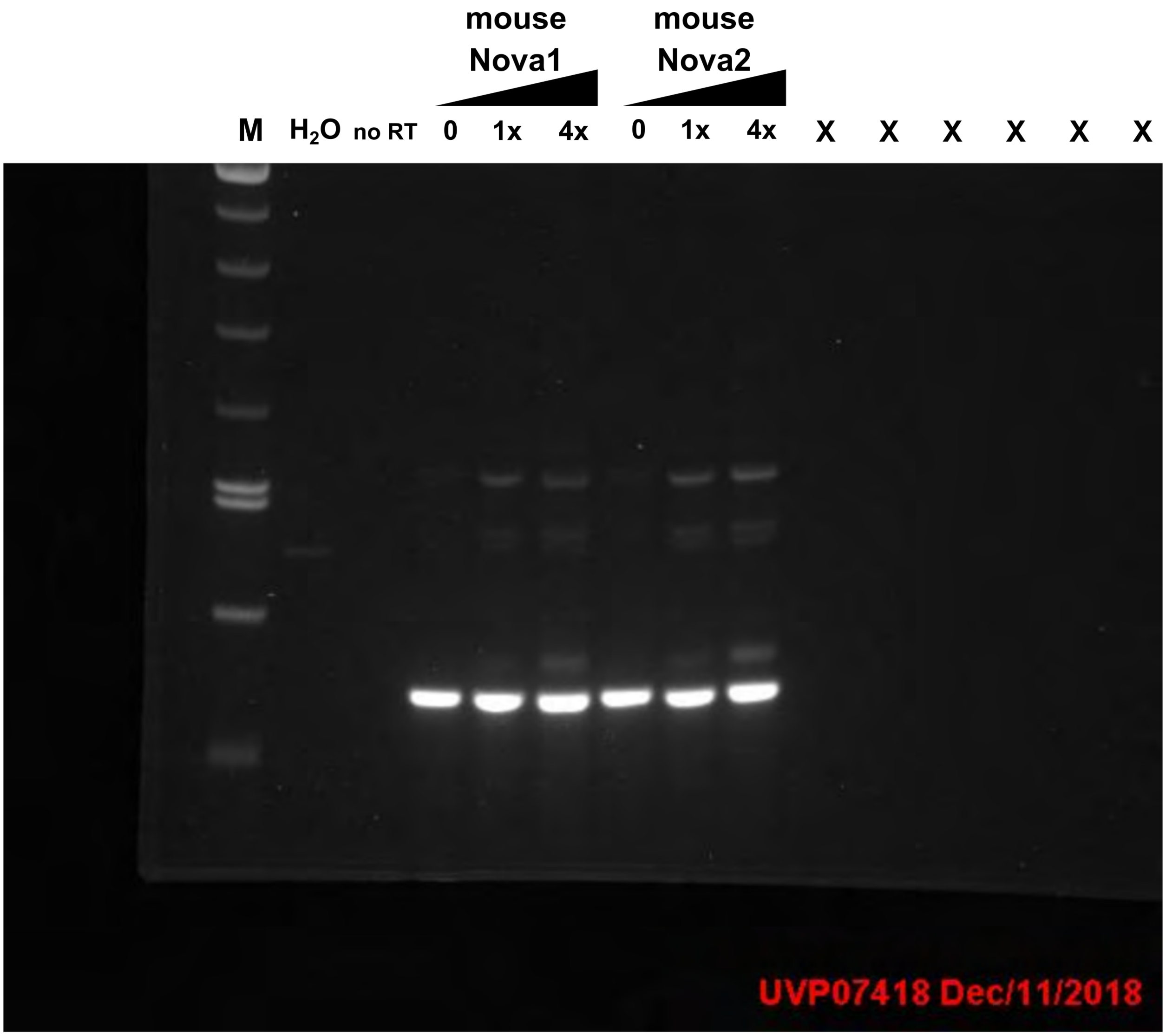
mouse
Nova1
mouse
Nova2
M
H_2O
no RT
0
1x
4x
0
1x
4x
X
X
X
X
X
X
UVP07418 Dec/11/2018

Figure 3C

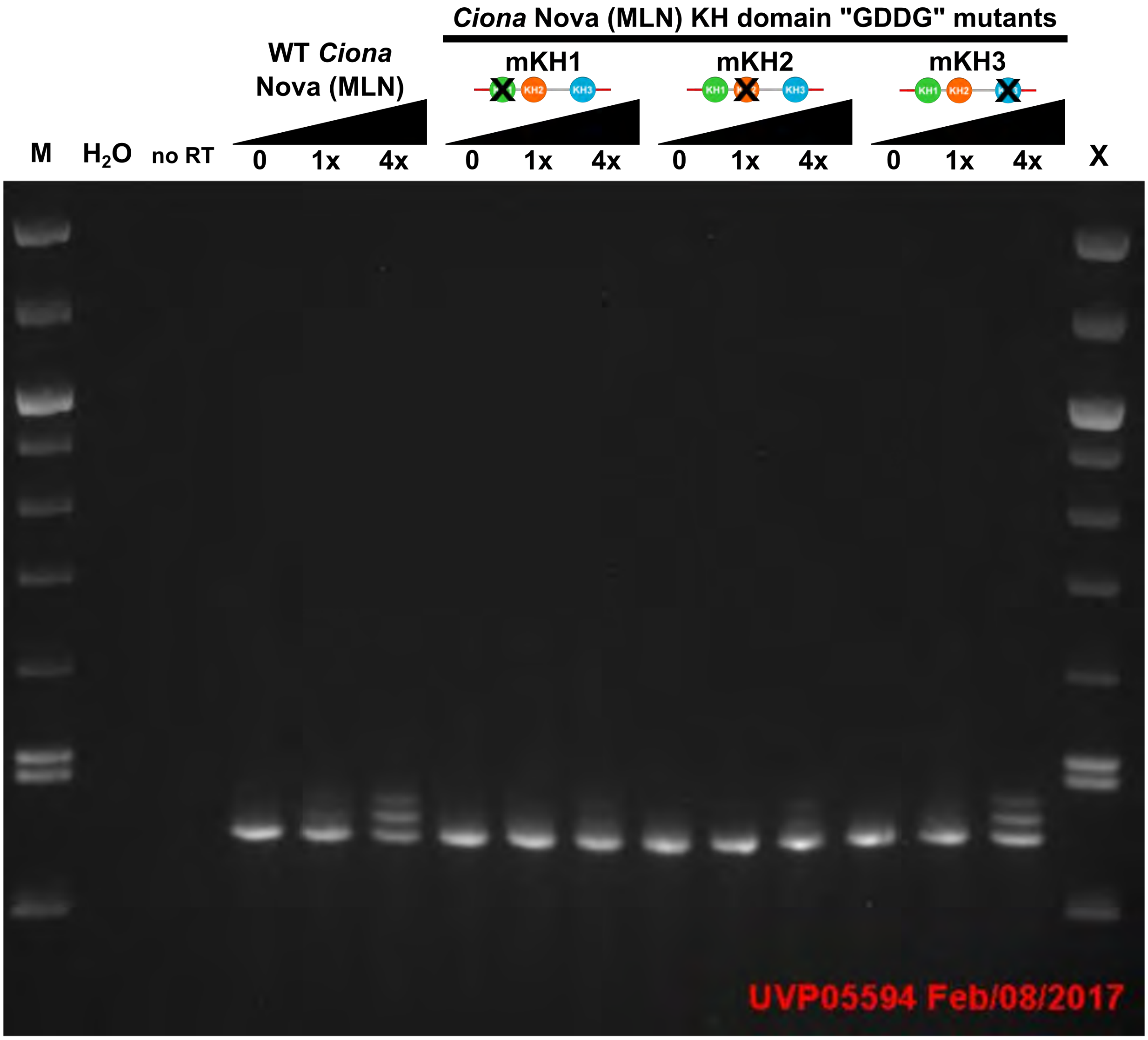
Ciona Nova (MLN) KH domain "GDDG" mutants
WT Ciona
Nova (MLN)
mKH1
mKH2
mKH3
KH1
KH2
KH3
M
H2O
no RT
0
1x
4x
0
1x
4x
0
1x
4x
0
1x
4x
X
UVP05594 Feb/08/2017

Figure 3D

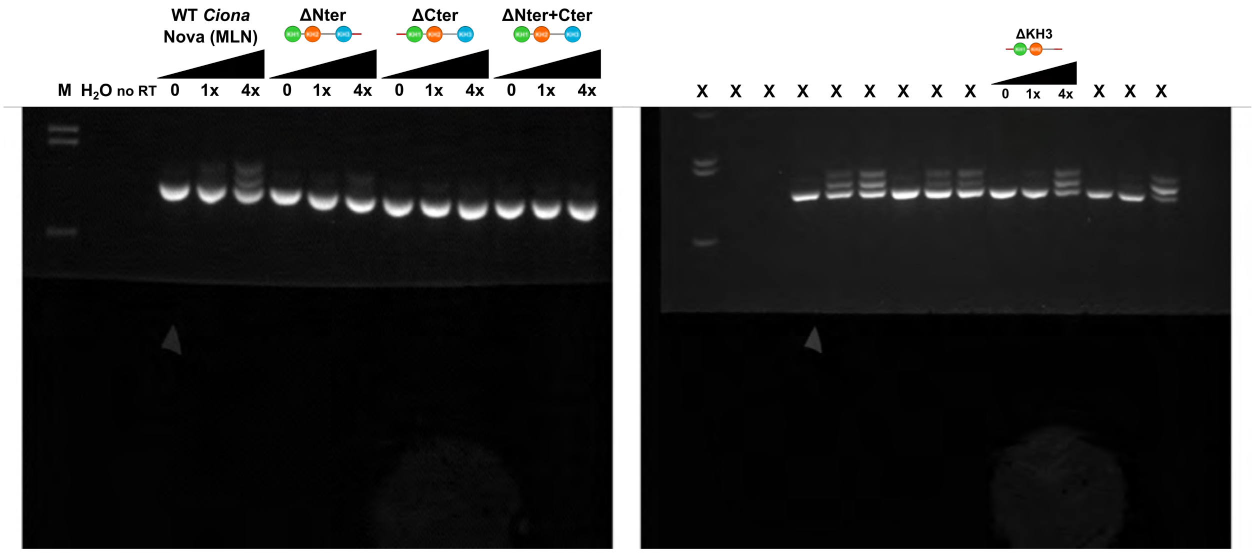

Figure 3E (composite)

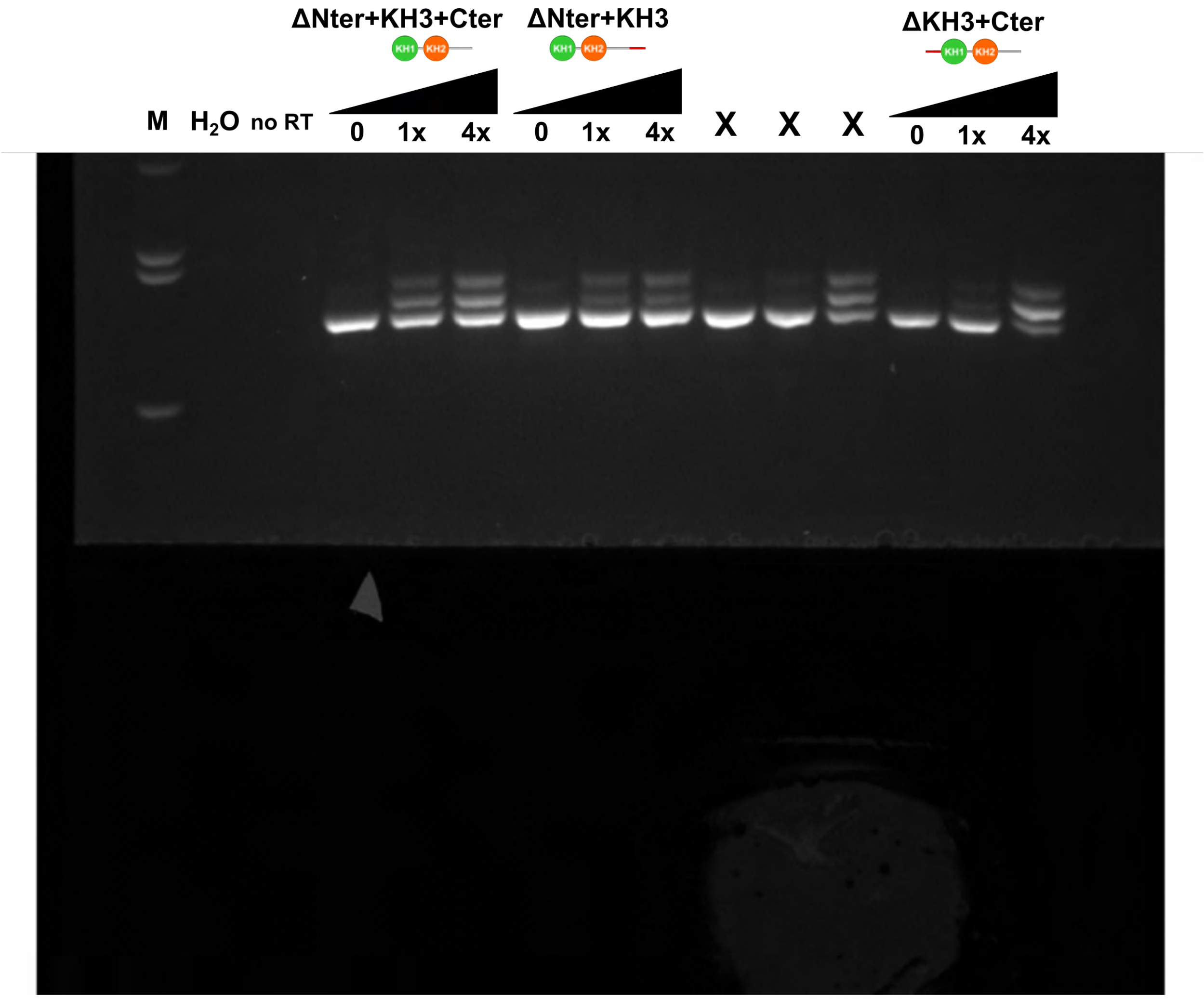
ΔNter+KH3+Cter
KH1
KH2
ΔNter+KH3
KH1
KH2
ΔKH3+Cter
KH1
KH2
M
H2O
no RT
0
1x
4x
0
1x
4x
X
X
X
0
1x
4x

Figure 3F

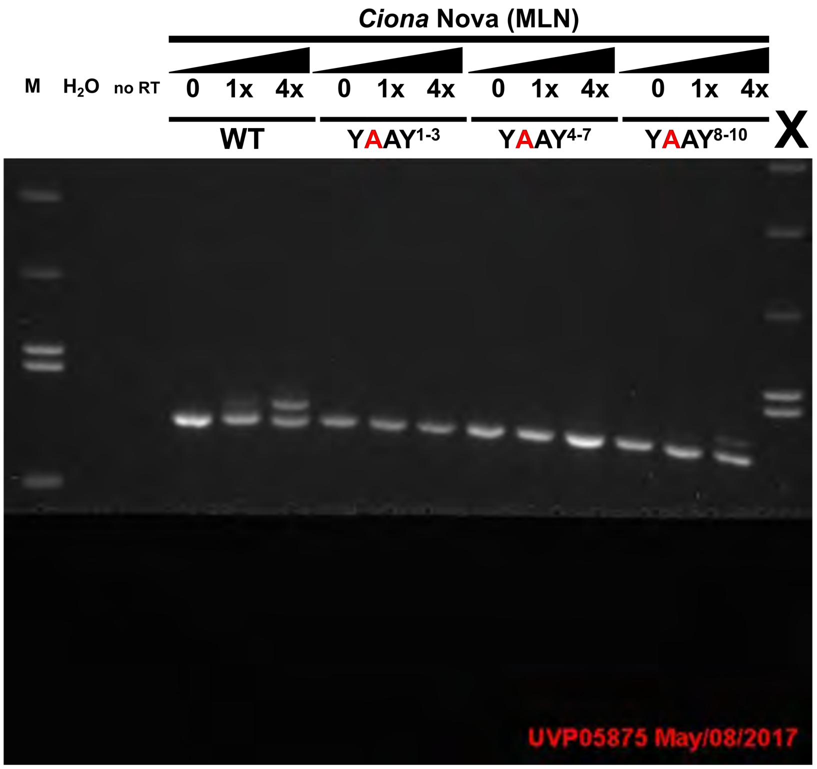

Figure 4B top panel

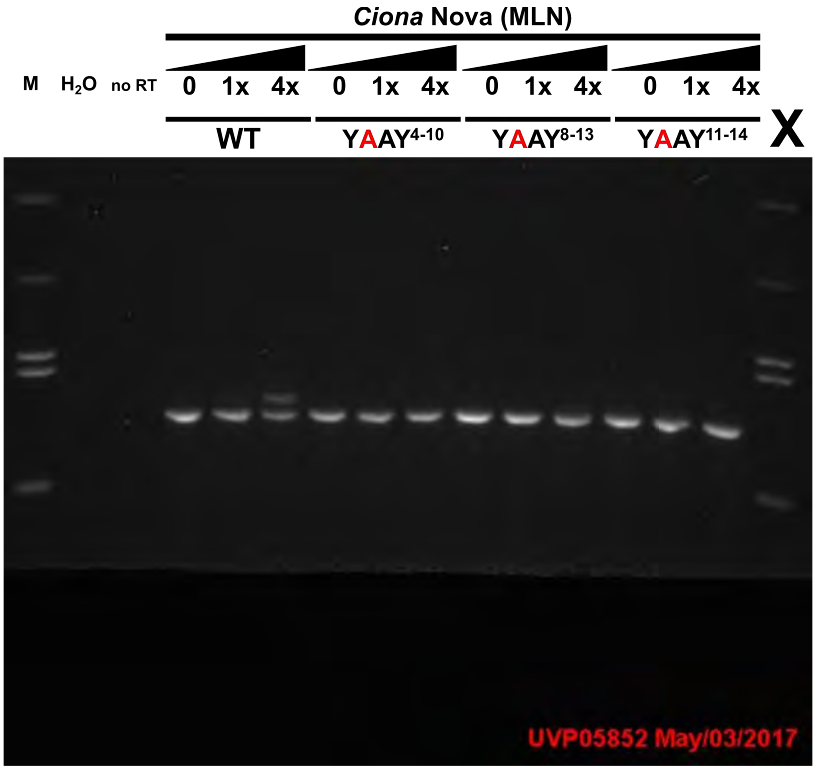

Figure 4B bottom panel

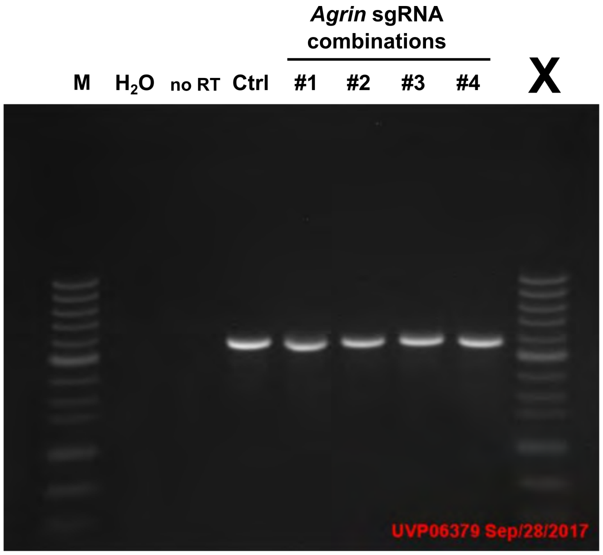

Figure 5B top panel

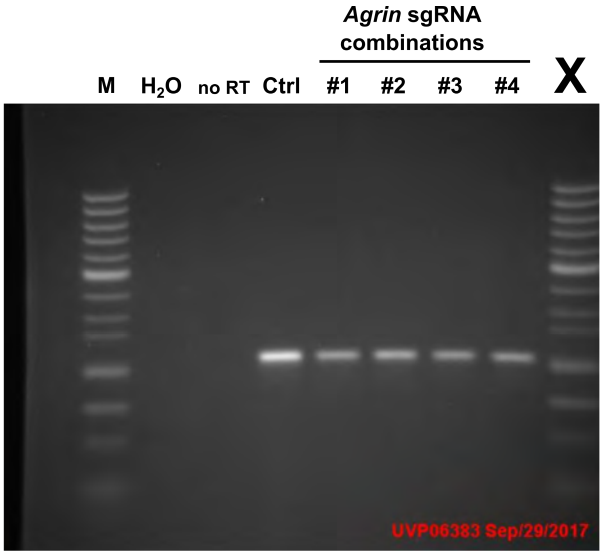

Figure 5B middle panel

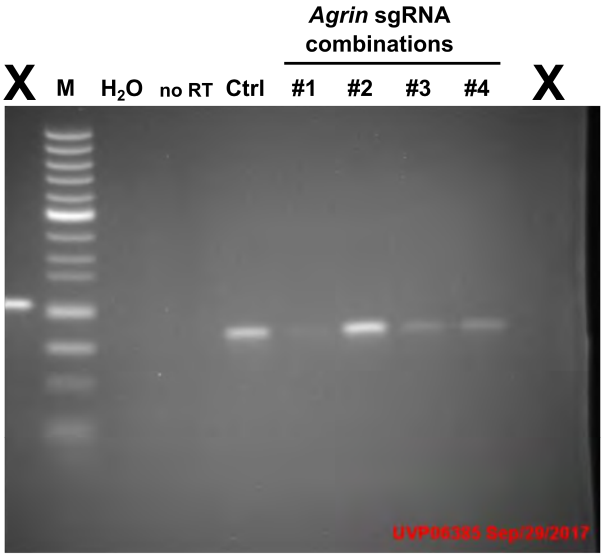

Figure 5B bottom panel

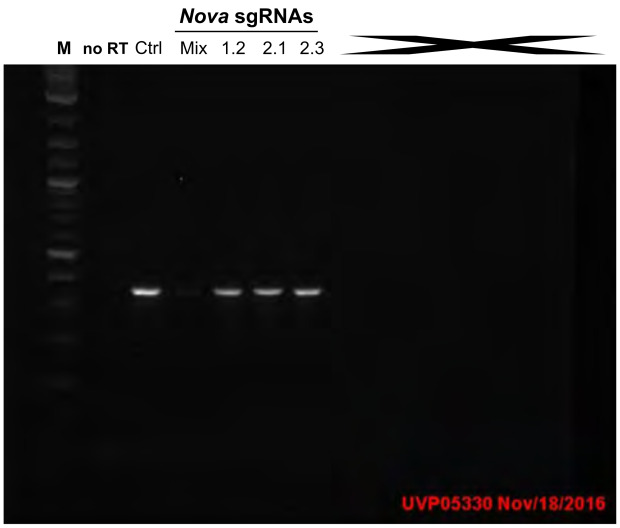

Figure 6B top panel

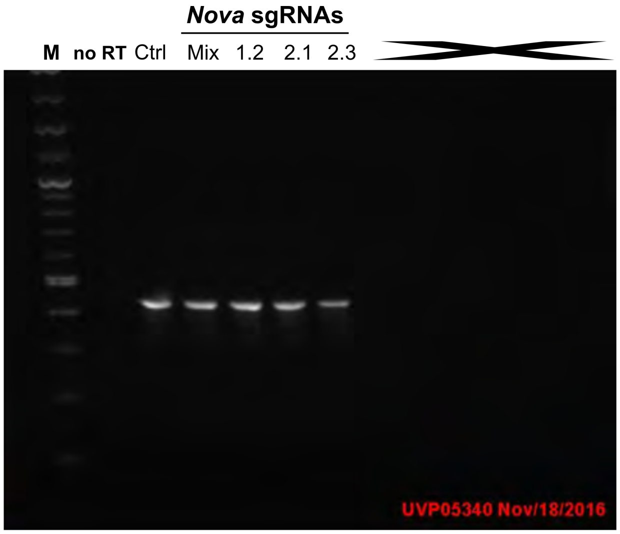

Figure 6B middle panel

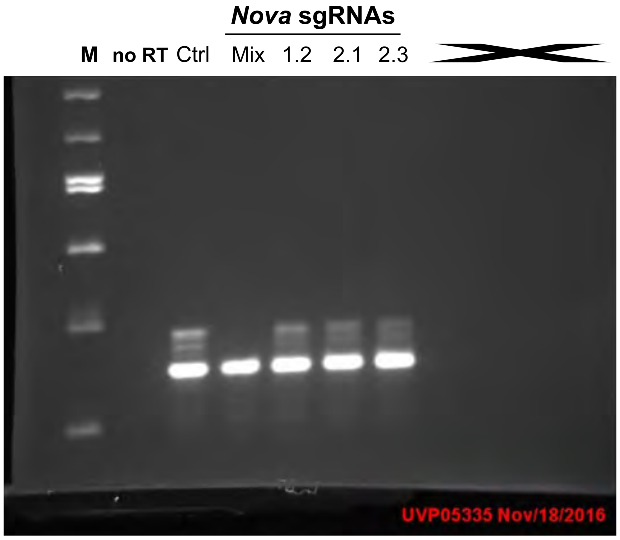

Figure 6B bottom panel

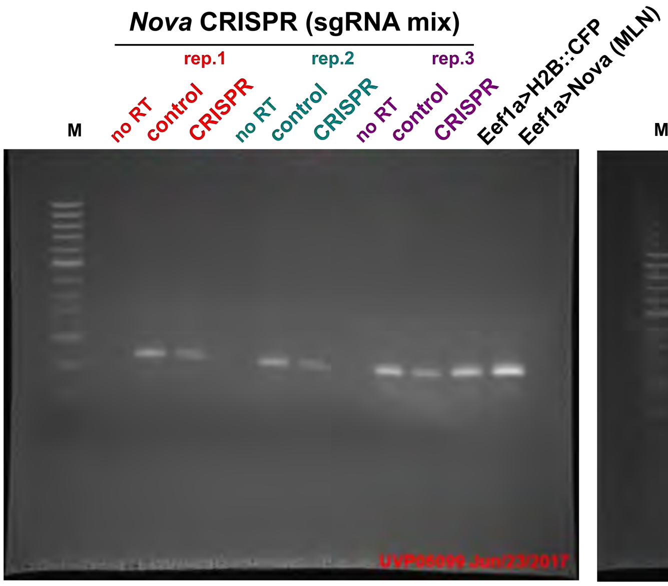

Figure 6C top panel

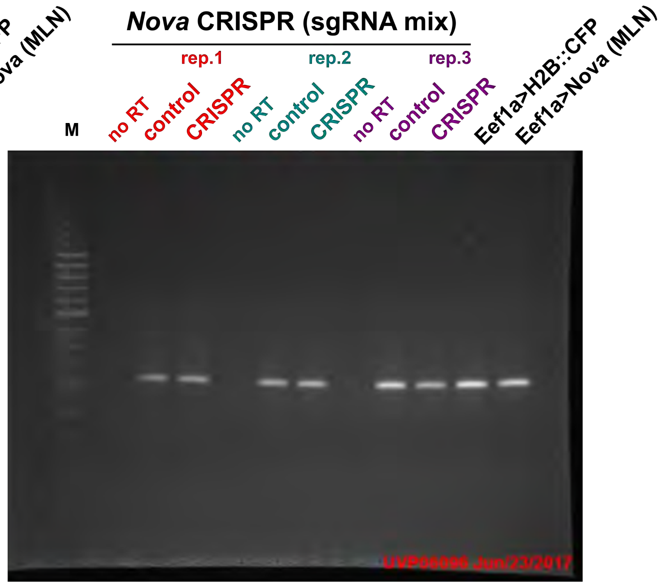

Figure 6C middle panel

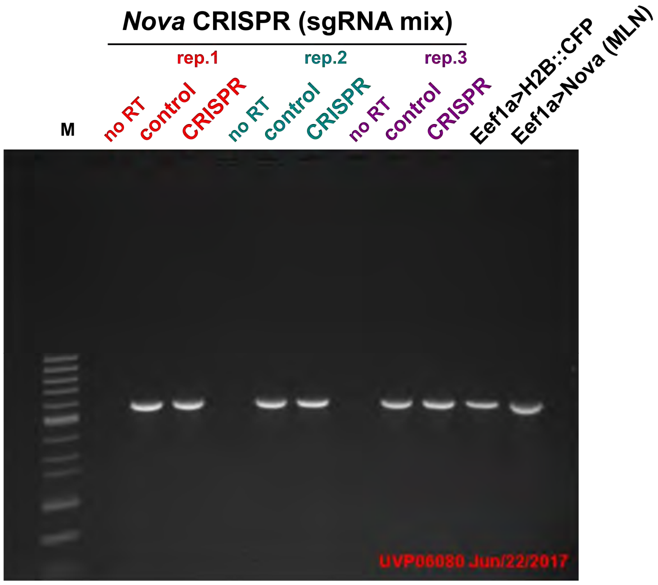

Figure 6C bottom panel

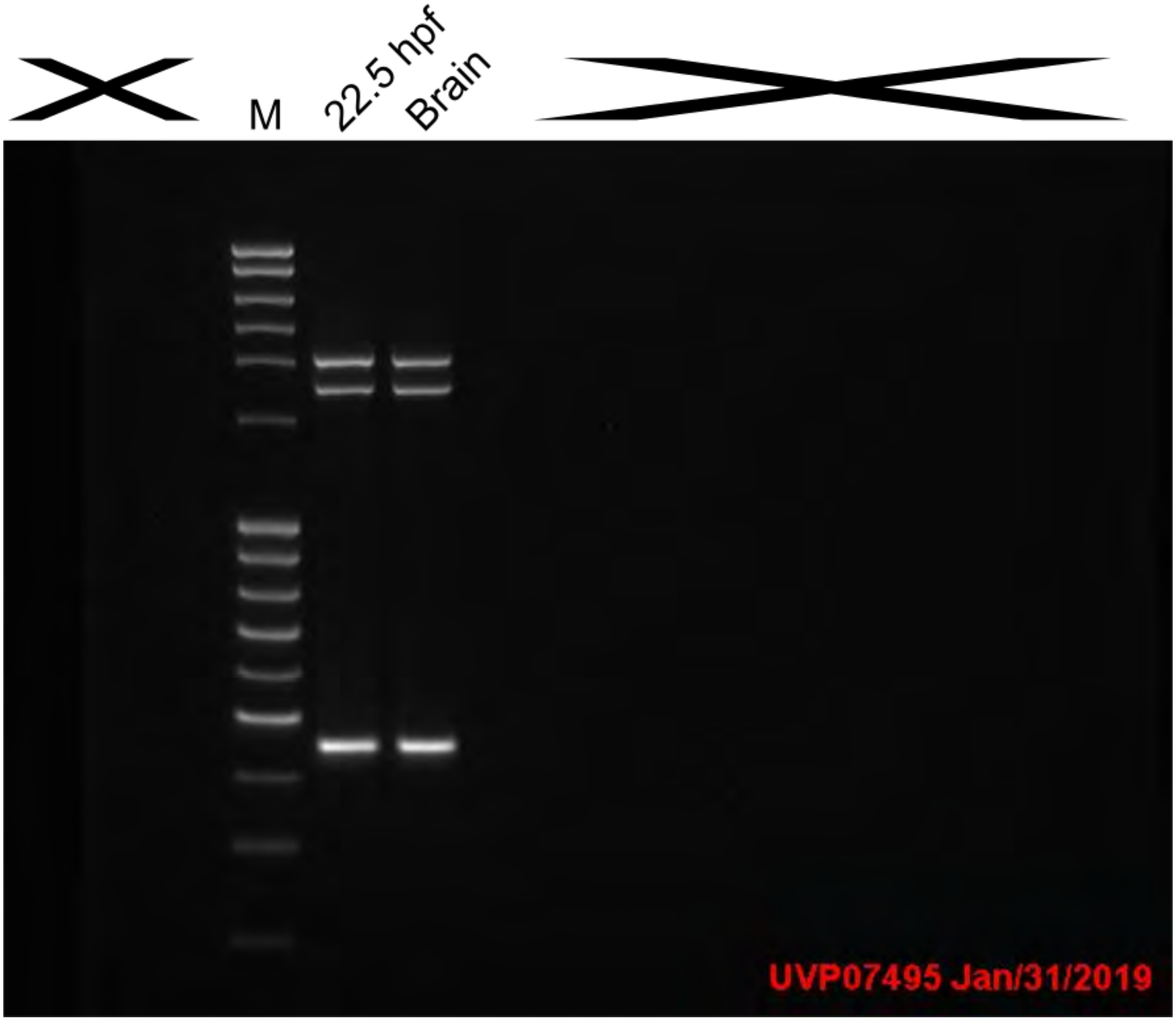

Figure S4

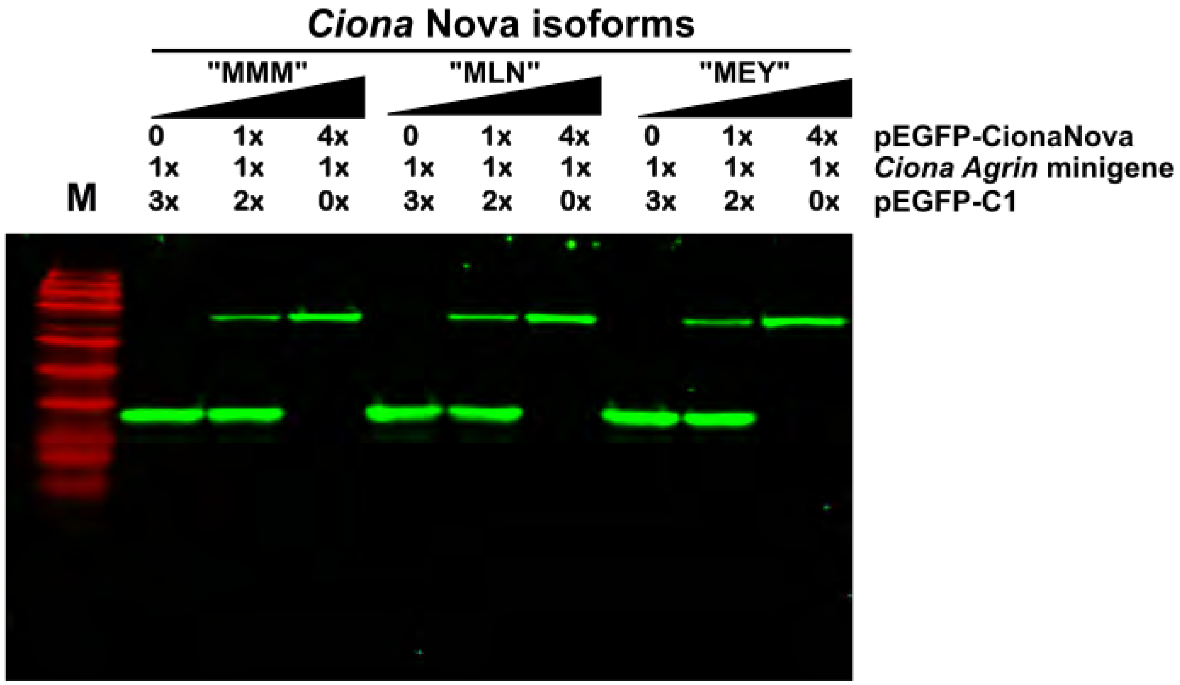

Figure S6A top panel

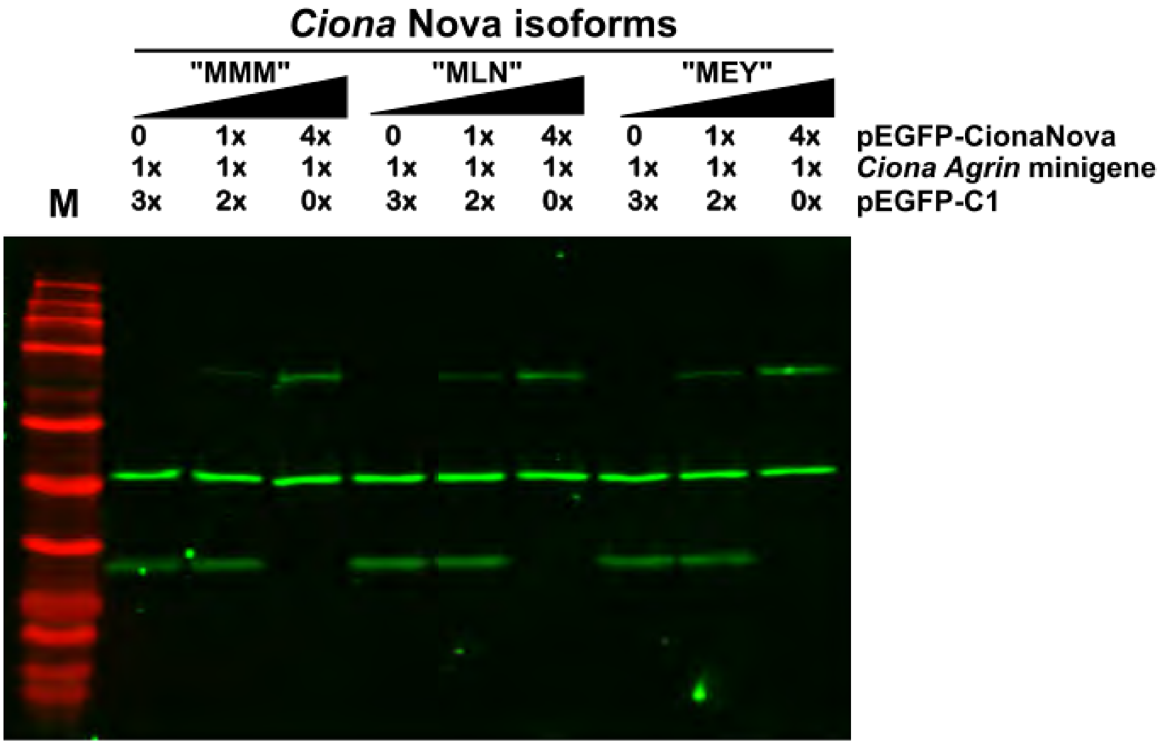

Figure S6A bottom panel

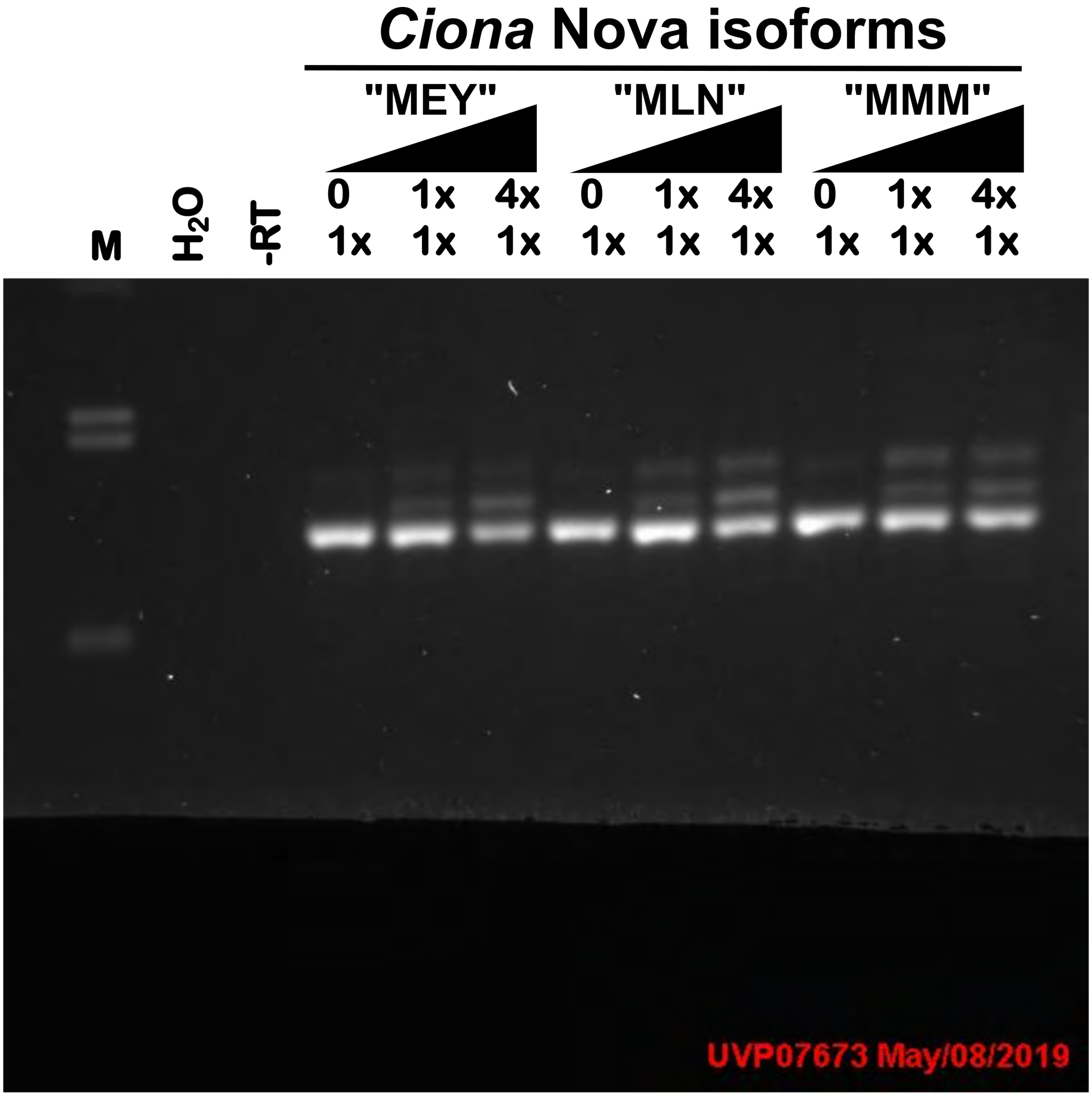

Figure S6B (“MEY” and “MMM” series positions re-ordered in final figure)

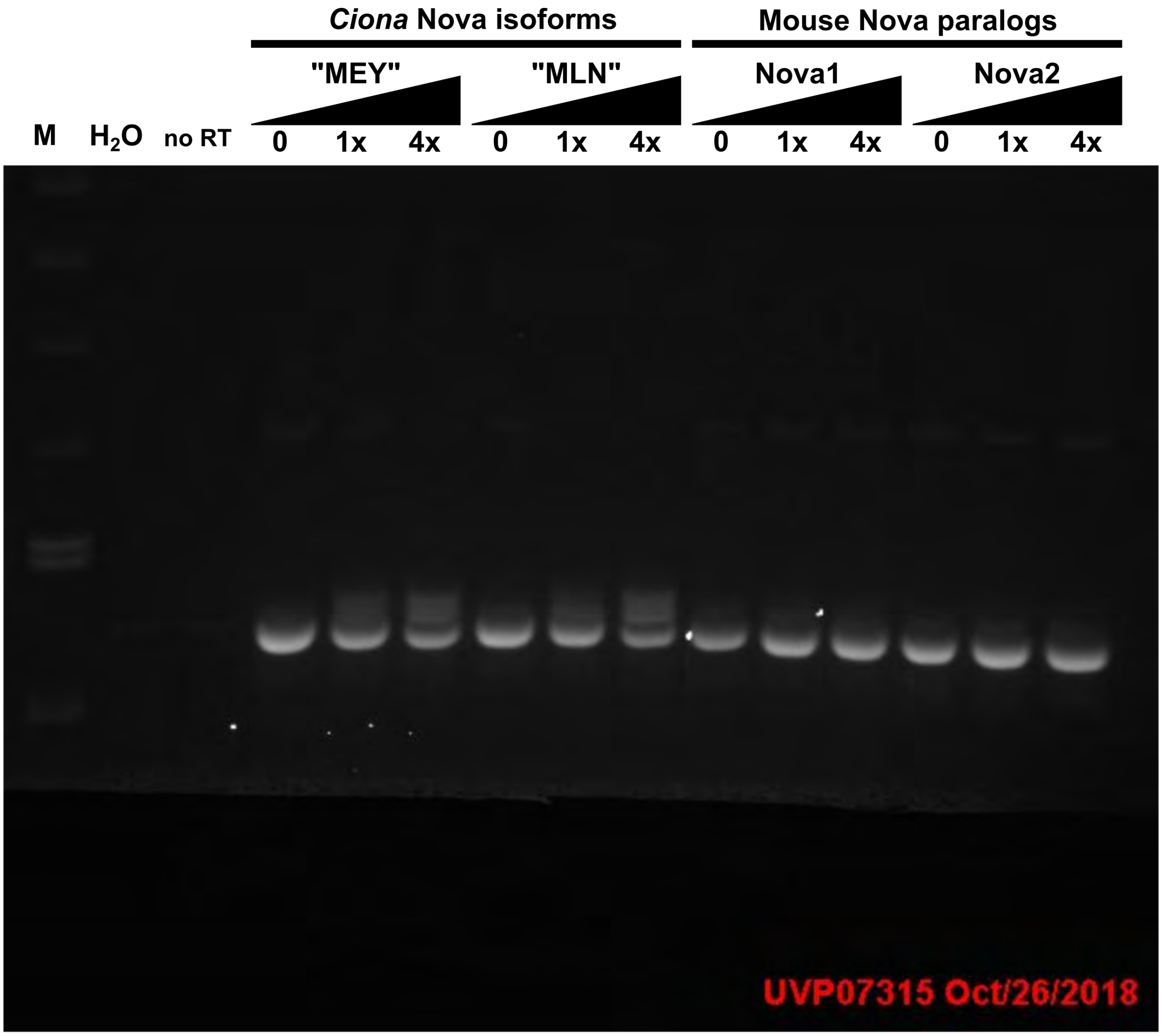

Ciona Nova isoforms
Mouse Nova paralogs
"MEY"
"MLN"
Nova1
Nova2
M
H_2O
no RT
0
1x
4x
0
1x
4x
0
1x
4x
0
1x
4x
UVP07315 Oct/26/2018

Figure S8A

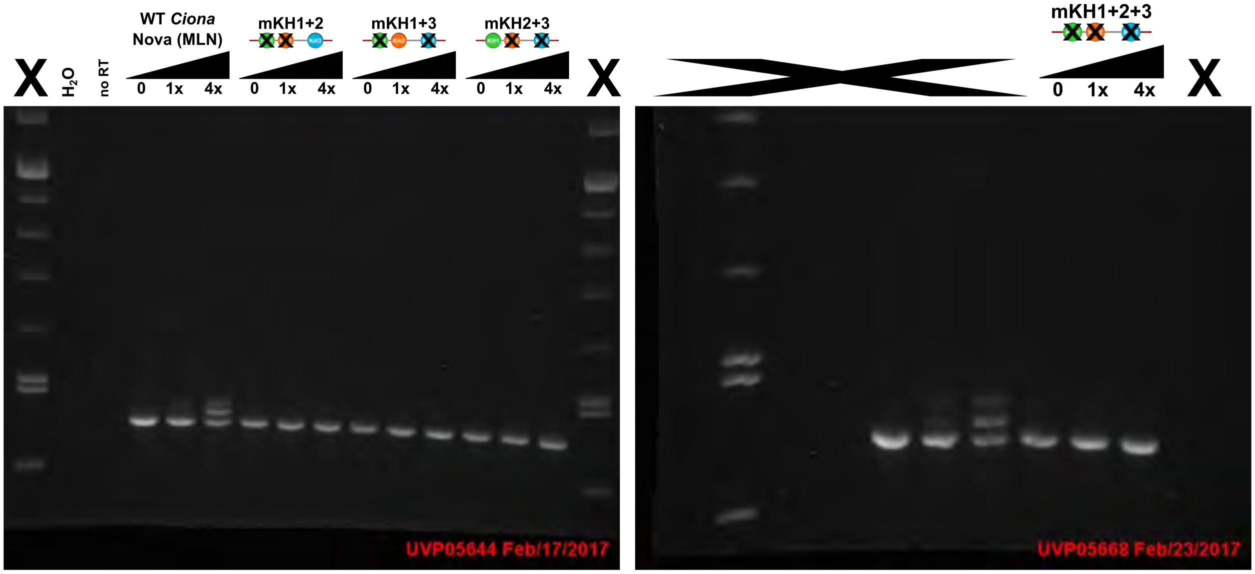

Figure S8B (composite)

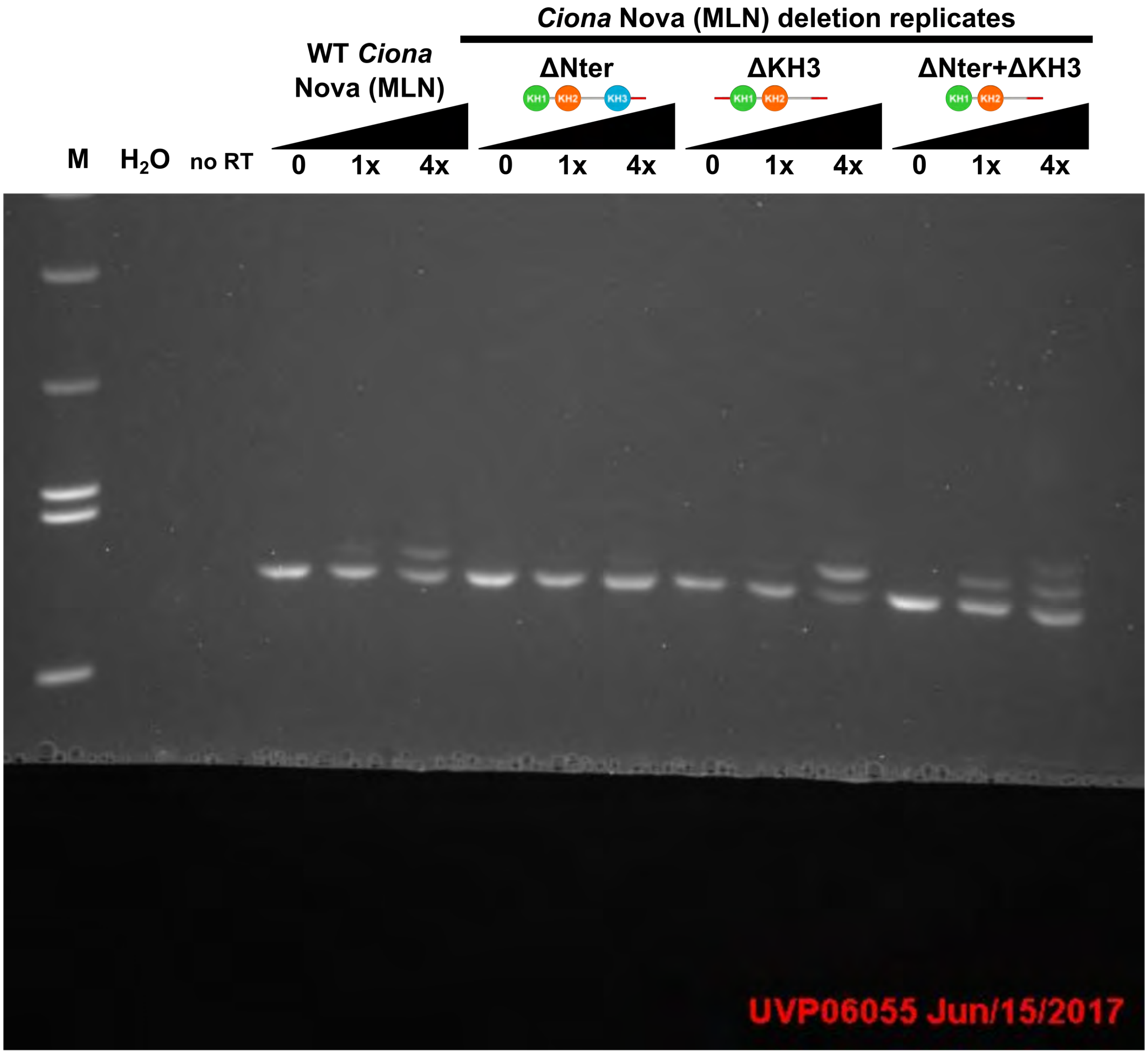

Ciona Nova (MLN) deletion replicates
WT *Ciona* Nova (MLN)
ΔNter
ΔKH3
ΔNter+ΔKH3
KH1
KH2
KH3
M
H_2O
no RT
0
1x
4x
0
1x
4x
0
1x
4x
0
1x
4x
UVP06055 Jun/15/2017

Figure S8C

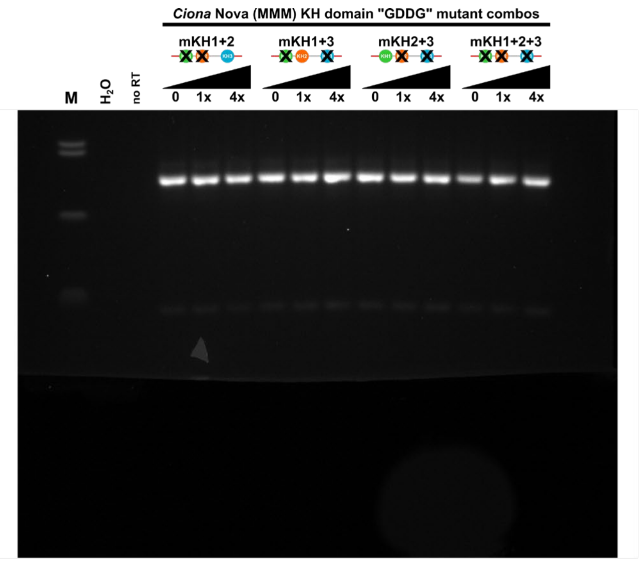

Figure S9 top panel

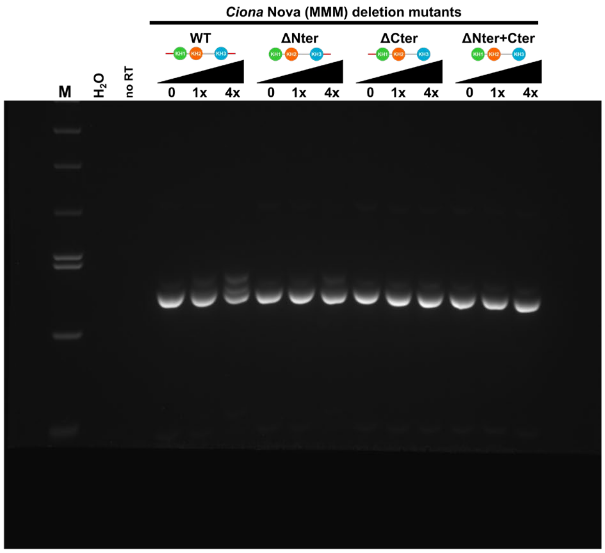

Figure S9 middle panel

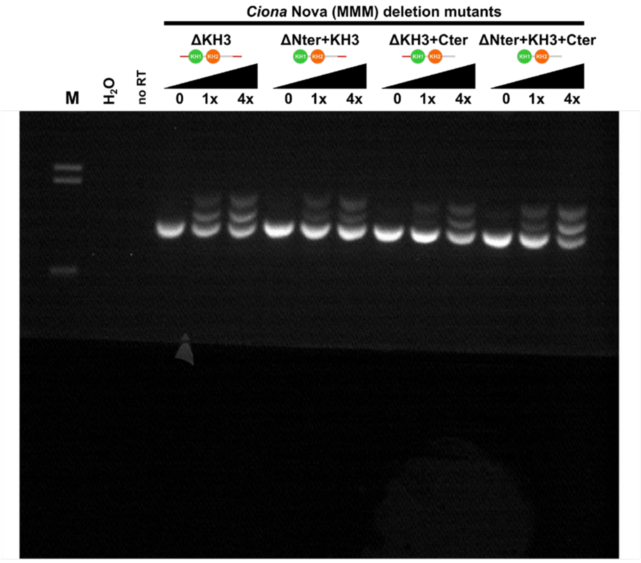

Figure S9 bottom panel

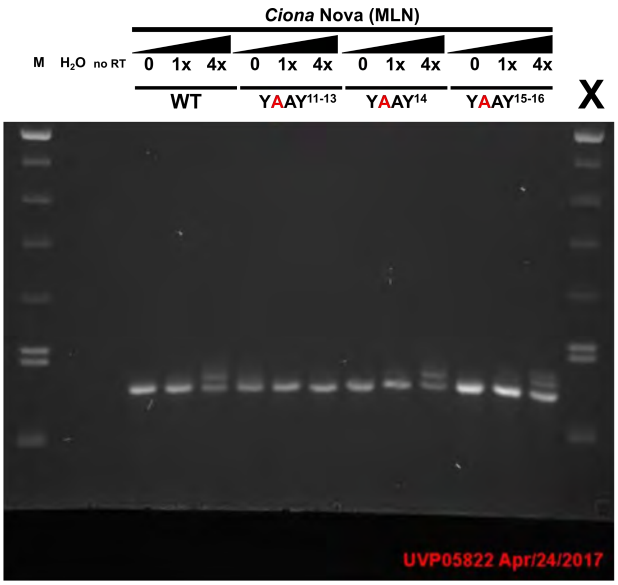

Figure S10 top panel

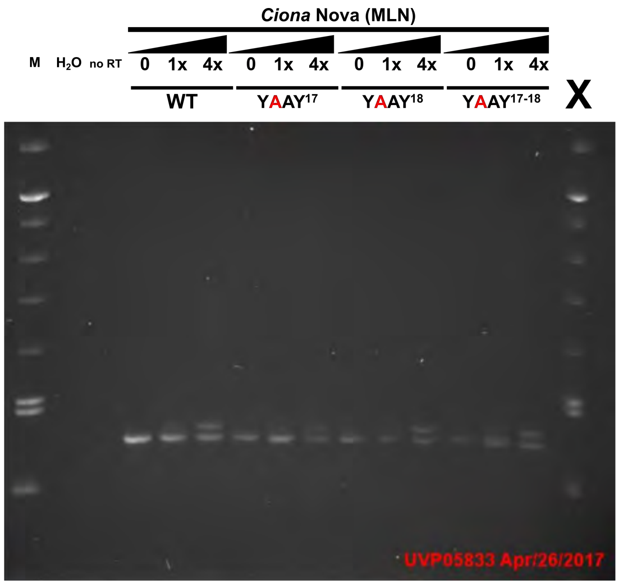

Figure S10 second panel (from top to bottom)

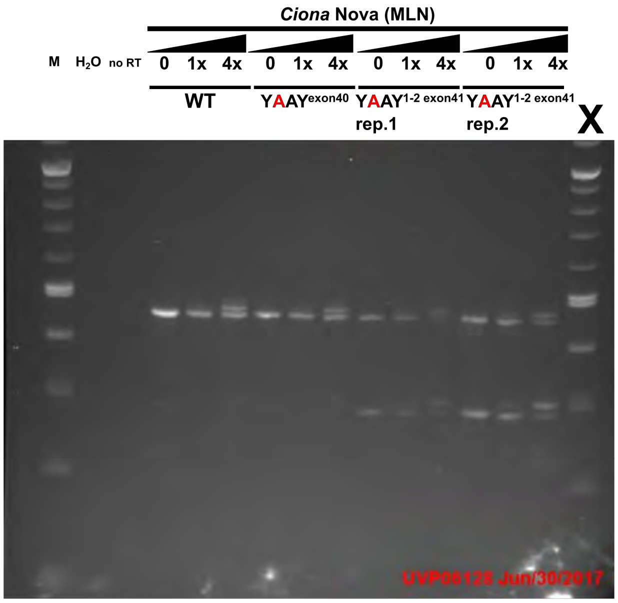

Figure S10 third panel (from top to bottom)

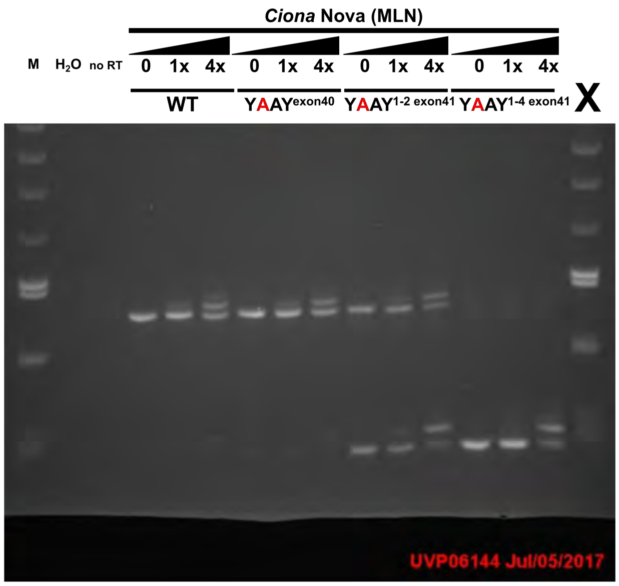

Figure S10 bottom panel
