# Supplementary material for: Neuron-specific Agrin splicing by Nova RNA-binding proteins regulates conserved neuromuscular junction development in chordates
Source: PLoS Biol. 2025 Sep 12;23(9):e3003392. doi: 10.1371/journal.pbio.3003392 (PMC12445529; doi:10.1371/journal.pbio.3003392)
Supplement: S1 File — (DOCX) [file pbio.3003392.s018.docx]

**Supplemental Sequences Files**

***Ciona* and mouse *Agrin* Minigene Products**

RT-PCR products from *Ciona* (pCiAgr 40-41) and Mouse *Agrin* minigene experiments were gel purified, cloned into pCR 2.1 TA cloning vector (ThermoFisher Scientific), and sequenced with T7 primer. The TA cloning site is flanked by EcoRI restriction sites (underlined).

*>Ciona* minigene RT-PCR product #1 (Z5)

GAATTCGCCCTTGTGTCCACTCCCAGTTCAATTACAGCTCTTAAGGCTAGAGTACTTAATACGACTCACTATAGGCTAGCCTCGAGAACATTCTACTGACTTGCTACAAGATGAACAAGCGACTGCCATTTATTTGGATGGAACAACTAAGATAATGTATAGGAATGCAGTGAAAGCAATGCAACTAAGTGACAATTCTCGTGCACGAACACACAACAATTATGAAATCGTGTTCCGCACTACTGCTCGTCACGGTCTACTGCTCATGGTGGGCAAAGCGAGGGAGGGGGTCGATTATATTGCACTCGCAATTCATGACGGTCGTCTTCATCTTCGTTTTGACCGTGGTTCTGGTCCAGCGCACGTAATATCCGACCAGCAAATCAACAACGGAGAATGGACAACTGTAAAAGTTAACAGTCTAGAGTCGACCCGGGCGGCCGCTTCCCTTTAGTGAGGGTTAATGCTTCGAGCAGACAAAGGGCGAATTC

Forward RT-PCR primer pCI_RT_F

Reverse RT-PCR primer pCI_RT_R

Exon 40

Exon Z5

Exon 41

*>Ciona* minigene RT-PCR product #2 (Z-)

GAATTCGCCCTTGTGTCCACTCCCAGTTCAATTACAGCTCTTAAGGCTAGAGTACTTAATACGACTCACTATAGGCTAGCCTCGAGAACATTCTACTGACTTGCTACAAGATGAACAAGCGACTGCCATTTATTTGGATGGAACAACTAAGATAATGTATAGGAATGCAGTGAAAGCAATTTCTCGTGCACGAACACACAACAATTATGAAATCGTGTTCCGCACTACTGCTCGTCACGGTCTACTGCTCATGGTGGGCAAAGCGAGGGAGGGGGTCGATTATATTGCACTCGCAATTCATGACGGTCGTCTTCATCTTCGTTTTGACCTTGGTTCTGGTCCAGCGCACGTAATATCCGACCAGCAAATCAACAACGGAGAATGGACAACTGTAAAAGTTAACAGTCTAGAGTCGACCCGGGCGGCCGCTTCCCTTTAGTGAGGGTTAATGCTTCGAGCAGACAAAGGGCGAATTC

Forward RT-PCR primer pCI_RT_F

Reverse RT-PCR primer pCI_RT_R

Exon 40

Exon 41

>Mouse minigene RT-PCR product #1 (Z8)

GAATTCGCCCTTGATAGTTGAGAAGTCAGTGGGGGACCTAGAAACACTGGCCTTTGATGGGCGGACCTACATCGAGTACCTCAATGCTGTGACTGAGAGCGAGCTGACCAATGAGATCCCAGCTGAGAAAGCGCTGCAGAGCAACCACTTTGAGCTGAGCTTACGCACTGAGGCCACGCAGGGGCTGGTGCTGTGGATTGGAAAGGTTGGAGAACGTGCAGACTACATGGCTCTGGCCATTGTGGATGGGCACCTACAACTGAGCTATGACCTAGGCTCCCAGCCAGTTGTGCTGCGCTCCACTGTGAAGGTCAACACCAACCGCTGGCTTCGAGTCAGGGCTCACAGCCTCGAGTCTAGAGACTACAAAGACCACGACGGCAAGGGCGAATTC

Forward RT-PCR primer mouse Agrin E31 F1

Reverse RT-PCR primer 3xFlag R

Exon 31

Exon 32 (Z8)

Exon 34

>Mouse minigene RT-PCR product #2 (Z11)

GAATTCGCCCTTGATAGTTGAGAAGTCAGTGGGGGACCTAGAAACACTGGCCTTTGATGGGCGGACCTACATCGAGTACCTCAATGCTGTGACTGAGAGCCCCGAAACTCTGGATTCCCGGGCCCTTTTCAGTGAGAAAGCGCTGCAGAGCAACCACTTTGAGCTGAGCTTACGCACTGAGGCCACGCAGGGGCTGGTGCTGTGGATTGGAAAGGTTGGAGAACGTGCAGACTACATGGCTCTGGCCATTGTGGATGGGCACCTACAACTGAGCTATGACCTAGGCTCCCAGCCAGTTGTGCTGCGCTCCACTGTGAAGGTCAACACCAACCGCTGGCTTCGAGTCAGGGCTCACAGCCTCGAGTCTAGAGACTACAAAGACCACGACGGCAAGGGCGAATTC

Forward RT-PCR primer mouse Agrin E31 F1

Reverse RT-PCR primer 3xFlag R

Exon 31

Exon 33 (Z11)

Exon 34

>Mouse minigene RT-PCR product #3 (Z-)

GAATTCGCCCTTGATAGTTGAGAAGTCAGTGGGGGACCTAGAAACACTGGCCTTTGATGGGCGGACCTACATCGAGTACCTCAATGCTGTGACTGAGAGTGAGAAAGCGCTGCAGAGCAACCACTTTGAGCTGAGCTTACGCACTGAGGCCACGCAGGGGCTGGTGCTGTGGATTGGAAAGGTTGGAGAACGTGCAGACTACATGGCTCTGGCCATTGTGGATGGGCACCTACAACTGAGCTATGACCTAGGCTCCCAGCCAGTTGTGCTGCGCCCCACTGTGAAGGTCAACACCAACCGCTGGCTTCGAGTCAGGGCTCACAGCCTCGAGTCTAGAGACTACAAAGACCACGACGGCAAGGGCGAATTC

Forward RT-PCR primer mouse Agrin E31 F1

Reverse RT-PCR primer 3xFlag R

Exon 31

Exon 34

**Endogenous Ciona Agrin Exon 40-41 RT-PCR Products**

RT-PCR products from Ciona at 22.5 hpf were gel purified, cloned into pCR 2.1 TA cloning vector (ThermoFisher Scientific),and sequenced with M13F primer. The TA cloning site is flanked by EcoRI restriction sites (underlined).

>RT-PCR product #1 (Z0):

GAATTCGCCCTTCAAGATGAACAAGCGACTGCCATTTATTTGGATGGAACAACTAAGATAATGTATAGGAATGCAGTGAAAGCAATTTCTCGTGCACGAACACACAACAATTATGAAATCGTGTTCCGCACTACTGCTCGTCACGGTCTACTGCTCATGGTGGGCAAAGCGCGGGAGGGGGTCGATTATATTGCGCTCGCAATCCATGACGGTCGTCTTCATCTTCGTTTTGACCTTGGTTCTGGTCCAAGGGCGAATTC

Forward RT-PCR primer

Reverse RT-PCR primer

Part of exon 40

Part of exon 41

>RT-PCR product #2 (Z6):

GAATTCGCCCTTCAAGATGAACAAGCGACTGCCATTTATTTGGATGGAACAACTAAGATAATGTATAGGAATGCAGTGAAAGCAATGCCAAACGATTTTCGAGATTCTCGTGCACAAACACACAACAATTATGAAATCGTGTTCCGCACTACTGCTCGTCACGGTCTACTGCTCATGGTGGGCAAAGCGAGGAAGGGGGTCGATTATATTGCGCTCGCAATCCATGACGGTCGTCTTCATCTTCGTTTTGACCTTGGTTCTGGTCCAGCGCACGTAATATCAAGGGCGAATTC

Forward RT-PCR primer

Reverse RT-PCR primer

Part of exon 40

Exon Z6

Part of exon 41

>RT-PCR product #3 (Z5):

GAATTCGCCCTTCAAGATGAACAAGCGACTGCCATTTATTTGGATGGAACAACTAAGATAATGTATAGGAATGCAGTGAAAGCAATGCAACTCAGTGACAATTCTCGTGCACAAACACACAACAATTATGAAATCGTGTTCCGCACTACTGCTCGTCACGGTCTACTGCTCATGGTGGGCAAAGCGAGGAAGGGGGTCGATTATATTGCGCTCGCAATCCATGACGGTCGTCTTCATCTTCGTTTTGACCTTGGTTCTGGTCCAAGGGCGAATTC

Forward RT-PCR primer

Reverse RT-PCR primer

Part of exon 40

Exon Z5

Part of exon 41

>RT-PCR product #4 (Z11):

GAATTCGCCCTTCAAGATGAACAAGCGACTGCCATTTATTTGGATGGAACAACTAAGATAATGTATAGGAATGCAGTGAAAGCAATGCCAAACGATTTTCGAGAGCAACTCAGTGACAATTCTCGTGCACAAACACACAACAATTATGAAATCGTGTTCCGCACTACTGCTCGTCACGGTCTACTGCTCATGGTGGGCAAAGCGAGGAAGGGGGTCGATTATATTGCGCTCGCAATCCATGACGGTCGTCTTCATCTTCGTTTTGACCTTGGTTCTGGTCCAAGGGCGAATTC

Forward RT-PCR primer

Reverse RT-PCR primer

Part of exon 40

Exon Z6

Exon Z5

Part of exon 41

**Published *in situ* probe templates:**

*Islet -* Cirobu.g00011396 (Satou et al. 2002)

**New *in situ* probe templates:**

*Nova (C. robusta)* *in situ* probe template

ATGGAGTATGAATGCCAGTACAATGCTGGCTACAGCATTGTGTCTAACGGTAACGAATACGGTCTCATACAGGCCTACACGGCACACGATTACCCCCTTGAAAACGGAGTGACGTTTTCAGCACCTCCGCCGGGCCAGCTCATTCTTAAAGTTCTAATACCGGGGTACGCTGCGGGGGCGGTGATCGGGAAAGGCGGTCAGATTATTGTACAACTTCAGAAAGATTCAGGGGCCATTATTAAGCTGTCAAAAGCGAAGGACTTTTACCCCGGAACCCAAGACCGAGTCGTTTTGATCCAAGGAACCGCCGAAGGCTTGATGAAGGTGCAAAATACCATTATAGAGAAGGTGTACGAGTTCCCTGTGCCCAAAGATTTAGCTGCGATCATCGGAGACCGACCGAAACAGGTGAAAATCATCGTACCCAACACAACTGCGGGACTGGTAATAGGAAAGGCCGGCGCAACGATAAAGACCATTATGGAAGAGAGTGGATCGAAGGTTCAACTCTCGCAAAAGCCAGACGGGGTAAACGTCCAAGAACGAGTCATCACAATCAAAGGAGAGAAGCACCAACTCATGACAGCATCTAATATTATTATTGATAAAATTAAAGACGACCCTCAAAGCGCCAGTTGCCCTCACATAAGTTACTCTGGCATCGCTGGCCCGATCGCTAACGCGAATCCCACCGGATCGCCCTACGCTGCTGGCTCGGCTGCATTAGTTGACGCTTCGCACCCATCCGTGGCCGCTATGTTGGGACATTATGTTATCCCAGGCCAACAGGTGCTGCAGACAGCAATGCCACTCTCCCATCACCCGCACCAGTCCGCGTTGTCCAGCGGCTCAGTGACACCGGCGCCTGAACTGACGACCATAAACCACGCCATGACAACGTTAGCGAACTATGGCTACACCTTAGGAGGCGTAAACTATGGTACCTTGGGTGTAATGCCTAGTGTACATCCAAGTGTACACCCTGGCATCGCTACCTCGGTCGGGATGATCTCTGCAGGCTCCCTAGCAGGAAGTCCAATCCCTTCAGCTACCCCCTTGCTCTCTGCCACTGCTCTACCGACGGAATCCAGTATTCCGACGGCTGTTCCCACTGCCCAAGCCATTTCAATGCAGAGCAATTACCTTGCAAACTTGGCTAATGCTGGTTACCTGACTACCGGTCACCCACAGTTGCTTGGAGCGACGTCAGGCCTCGGCGGTCTCACCACAGTGTCCCAGCACCCGCCACCAGCGGCGACACCAACGAGTTTTTCCGTCGCTTCTACCCCTTCTACCCCTGGTCTGCCGGTTTCATTTAGCCCCCATTCAACCGTGAGTATCCTAAGCATCGAAAAGTCAAGCGACGGACAAAAAGAAACAATTGAACTGGCAATTCCCGAAAACCTCATCGGAGCAGTCCTCGGAAAAGCGGGAAGGACACTGGTTGAGTATCAGGATGTATCAGGGGCGAAAATTCAAATTTCTAAAAAGGGTGATTACGTCGCCGGGACCAGGAACAGGAGGGTTACGATTACGGGGAAGCCCCCATGCCCACAGACTGCGCAGTTTCTTATTACGCAACGTGTCGCCTCTGCGCAGAACGCAAGGGCACAGCAGGCTAAGTTACTGTAGGTCAGGAACCACGCCAGTCTTCCACAAATGTGCTGCCGCATATTGTTTTACCTTAGTAGTTTGTACTTTTAGTAAGGTTGAATTTTTCGCACTGAGGAGCGCTAAGTACGTTTCTTAAAAAGTCTCTTCGCCAAATCAAGTCTTCCGGGCCATTCGCCAAAATCGCATCTAAATTCGATCATCTTAAACAGCGTACACGACTTTGCTTTTTTCGGCGTTCACGAAACGCGCTCATACATTATTCAAGTCAATTACGAAATCTCTGCGAGTTTGTGACGCACTACTGTGCACTACCATCGATCCCGCTTTTCCGTAT

*Agrin (C. robusta)* *in situ* probe template

CGTTGGTTTCAGTGCGTTTAGGATTGAGTTTCGCTTCTTAAAGAGAAAAATACAGCGCAAAAAAAGATAAAACAATACAGTGACGGGGTAAAAACTAAGAAGCGAAAATTTGTTCGGTCCCCAGGAAAATAAAAAATAAATAGAGCATGCTACGGTATGGTACAACATGTAGAATTATCTAAAAAGTTCAAATAAGAATATGGAATGAAAGGAGACCGAGAGAAATCGACAGTACGTGCGGTATACACCCTCGCAAAAAATCAAAACTTTTTTGTCGTTTTTAAAATCGAAAATTATAAATGTAAAGGCTTCGTAACCTTAATAAAGTGATTGTTCGAAACGATGCAAAACACGAAAGTGTGAAAATTGAAATAAAATGCTAAAGGTGAAAATATGGTAAATGTATTGAAATCTGATACCCATGCTTAGACAACAGTAAAAACTAAGCGCCTAAATTTGGTGGCAATATTGTAAAGAAATTTTGCGCCAGGTATTTAACCAGTTCAAATGAAAATGTATTCACTTGTTACTATATTAGAAAATTTAAAAGACATTTTGTTTTTTTTCATGGTGATGCAAAATTGAAAAATTACCTAAAATGATTTCCGTTTCGAATACGTTAGTGGGTGTGGATAATAAAGTGTGAAAGAAATTGTTTTGATGGTTGTGCTGTGCTCGAACCTACGGCGCTGTCAGCTTTAAAATCTGTGGCTTGCGTGTATCCATCATCGACCATTACACGAACGCACAACGGGAGAATTAAGCGCGTCAGCCCGTAAATCAATTGATTCGTCGTGGATCTTTGCTGAGGCCACGCAGCCGACGAACGGGACAAAGAAAGATTTGTAAAGACCAATACCTCGCGGTCGGTAGTCAACGCCACCTAACCACAACATTCCATCAGAGTTCAGGTGATTAGTTAACCCTGGTGAGGTCGCTGTCTTTATTAACCCGTTGTTTACTTGCAAACTTCCTATGTTCATTTTCCTGTTAACTTTTACAGTTGTCCATTCTCCGTTGTTGATTTGCTGGTCGGATATTACGTGCGCTGGACCAGAACCAAGGTCAAAACGAAGATGAAGACGACCGTCATGGATTGCGAGCGCAATATAATCGACCCCCTCCCTCGCTTTGCCCACCATGAGCAGTAGACCGTGACGAGCAGTAGTGCGGAACACGATTTCATAATTGTTGTGTGTTCGTGCACGAGAATTGTCACTGAGTTGCTCTCGAAAATCGTTTGGCATTGCTTTCACTGCATTCCTATACATTATCTTAGTTGTTCCATCCAAATAAATGGCAGTCGCTTGTTCATCTTGTAGCAAGTCAGTAGAATGTTCTTGCTCACAGTTATCTCCCGTGTAATAAGGTAAGCACACGCACATATATTCTGCTCCACGTGGATGGCATACACCACCATTATCGCATGGGTTGCGATAACATGTATGGGCGTTGAATGCAATTACATTGAAGTAAGAGACACTACCAGCTGGGGAAATGGGCAGGTTCACACCGTTCACTTGGAACTTCTGTAATGCACCACTCAGTCCAGTTGTCACTCCAGCTTCTGGATTAAATTTGACACCATCAGGGAACCCACCAACATACATAGGTTGCTTAAGGTCTAAGAAGGAGTGTTGGCTCGGTGATGTCCCATACACTGGATCAAAATTATCCAATGAAAGGTCTCCAGTTCTCATGGCACGAGATAACACCACAATATGCCATTCATTCAAACTTACAGGGTTGGCACTCCTGATATTTGCTGCCCCTTGTCCAAGGTTGTATTTAAACTCAAGGAATCCATTCTTTAGATTGAGGGAAACAAAATCTCCTTTGCCAGATTTTTTCTGCCCGTTGTAGAAAATTAAACCGTCAGGTTGATTTGAATAAAATAAGATTTCTATTGACATGATTGACCGAACATCCTTTCCTAAAGATGGAAGTTCCAAATATGAATCGCCGGCAAATGCAG

**Predicted Agrin protein sequences from other invertebrates:**

**Putative Z exon in bolds** **Predicted Z exon-encoded NxI/V/F motif**

>Ciona robusta (Z5+Z6)

MPGSTKISFNLEMCRGRTNMTMYIRTLILLLSFLYLINTGAECYRIKRAVMLSMDPQPAVECLDRTTTEKNSTSTVILTGTVQYCDVIRAGSYKCNIKIWRVMKGNSLAMNLITIQKQGILTSMYKKKYVDVYGLGNSAFCKSNVERADTRIFFLNKLNNRLVVSASLDRITLKNLENTQKVIEGKRIKEEPKKPRDPCQEVLCGYGASCQAVDEDTTTCVCPPVSCSASPTTEDILAPICGSDGVTYPNECRLRAAECMAQHRIKIKATTSCEPVDDRLCSTVTCDFNAACVVRPSDDGSLAPECECPTCENVAVEPVCGSDQRSHRSSCDLRRASCVEGRVITVVRNSACNPCDIFNIESGIFLPMCSGSCTLDGNGEPQCCETLKCAANETELVCGSDGNTYENECSLRRSACKTAKSITVIKQGSCPTKKDVCMDLQCEHGAMCKITKSGSTLTPSCSCENVGAAESLPVTHACKQATDGNGVHRIFMNEAEARGESCTSNQILDFKDICQGGCSGVGEACIVMNGVGSCQCITCGEEYQPVCTNDGQTFRSHCEVRRHNCLSKTNLIILSIGSCESQCDDIKCWYGAQCEVIAQGTTCVCPTVCVKTYLPVCGSDGQTYSNECEMVVAACPQKLEVTVAHAGPCDEPTHEGSGSGAGDECGCLFGASCDTTIDDEDANCVCNFNCEAIGVAVCGSDGKTYPNMCELEKAQCNQQTPISLVSKGICKGRIGCVQSRYGCCEDGTTPAKGVARSGCPEKCLCNEHGSYGNACNPTTGQCVCRPGVGGLRCDRCRPGYWNFRALAEKLFTGCMSCRCHEFGSTRDDCDQMTGRCSCKVGVTGLKCTDCVSDGSWVTSSVCDNAAAPSAVSCEDLVCRVGQECVEINNAFECQCPSLTSCDNMDEAVVCGTNGITYADRCQLKVLACKVGVNVTVAHEGACMSKINPEPTPNAKPEPTPKSEPEPEPTSKPEPEPEPTSNPEPEPTPNAKPEPTSNPEPEPERTTKTPLVPKSEPETLATSKATPATTVLPVPTTTLSVTKTPKTTAGKYLSKYSSIFICCSTDEVECMYGDDEDCGGSGSGEGVSTFYSIDGNSLRHSKSLDVPGSEEFINYSNLVEAEIMSLISNPPLLDSVRAVRISSFRSASIFWGGVIVTFELHLTSGSDAGAIQDALNAAIPIAFFCHQLIAWLLAFFNLPSLIAHILFPLFWSGLCVSCVTVTSALDITWQTRLTLSTLCFIYPKLTAKACRNRFVYVGCLGKQNMFVHDAPYEPVFESVWFDCFSCGCFYFGLAFFTFVKRLKTCSFFIYYIPDGPWTVPHFSGASYAEFRKVNAFSEITIQLKFRSADPEGILFYSGQLNNGRDFISLAINNGYVEFRFDMGSGMLKLRSKRPINSTQWHTIVARRIRRDGMLQVDADPPVQGTSPGHASGLNLDSNFFVGGFNTYLEDQYQKQTGVDKGLTGCIEEVRINENQLNITSNSDHCVSTFRLAECGASPCIPNPCQHSANCFITMQANIFTNKCECKDNYEGKNCLENTGLS

VKKKPNNPCSPNPCQGGAKCIEMPGEEEFTCKCPPGRSGSLCMTNQSAALQGPSFMPAFAGDSYLELPSLGKDVRSIMSIEILFYSNQPDGLIFYNGQKKSGKGDFVSLNLKNGFLEFKYNLGQGAANIRSANPVSLNEWHIVVLSRAMRTGDLSLDNFDPVYGTSPSQHSFLDLKQPMYVGGFPDGVKFNPEAGVTTGLSGALQKFQVNGVNLPISPAGSVSYFNVIAFNAHTCYRNPCDNGGVCHPRGAEYMCVCLPYYTGDNCEQEHSTDLLQDEQATAIYLDGTTKIMYRNAVKAM**PNDFREQLSDN**SRARTHNNYEIVFRTTARHGLLLMVGKAREGVDYIALAIHDGRLHLRFDLGSGPAHVISDQQINNGEWTTVKVNRKMNIGSLQVNNGLIKTATSPGLTNHLNSDGMLWLGGVDYRPRGIGLYKSFFVPFVGCVASAKIHDESIDLRADAFNSPVVRSCNGR

>g13824.t1_prot length=2425 - Branchiostoma lanceolatum (Amphioxus)

MTTTRSGRTIRPPQRLREAESAFDEEFQRVEDEISELRASLAATSTPVVRLTPLHTSVSQEEALERERVALLEEIRTLELAKEVESLRRRKDNLLVQSDARSGGVVTPLPKTANPVADSQHLAKNISPDVQHLAAEPTIQDQRRDQASAAEIEKSSPLPKTANPVADSQHLPAKNTAPDLQHFATEPAILRRDQASVTEINRILCDLNLTSGGRPSASASPRAYGKKISGFFRTPRSSSRITKHVIWPQELLPAFSTESAKLTYDMLTYDQFIAGYMRLFNSDLLQPVELSERLKLVQKCAEYTLRYPWDSVRDYHGEVMRAVEEGQFDWGDSDYNTHVEEMFKMYVQPFLQSSTPARRVQSLSQLQQTIKYCKLFQSRSCPQAELIHDDVQFGEVHHNVYNVHSVNSCGHDMFNVNYDNSHDHDVNDPSPMLCLTDNRTLCDVHYCYDMLYNDSLCVNSMNECSVMLHNDSLCVNSMNECSDILHNDSLCVNSRNECSVMLHNDSLCVASMNKCSDILHNDSLCVNSMNECSVMLHNDSLCVNSMNECSDILHNDSLCVNSMNECSDMLHNDSLCVNSMNECSDILHNDSLCVNSMNECSVMLHNDSLCVNSMNECSDILHNDSLCVNSMNKCSDMLHNDNLCVNTMTECADMLHNDSLCVNSMNECSDILHNDSLLCVNSMNECSDILHNDGFCVNSMNECSDMLHNDSLFSGDSSPNLRLGLDIDLSEGEPFTTREPPAPKDPCIAVFCGFGATCDPTQISDAGKGTCICEQTCSSGVIAPVCGSDDVTYSSKCNLELKSCQTQSRITVKKEGKCIQDPDSNPCYGKDCRYGSSCVVDGSTAKCVCPTCEEQSESTVCGDDGNDYKNECEMNKAGCSKKEEITLRFRGKCNPCDGFSCDRGVCILDDNRNPQCSCKQECSAPDPLARICASNGKTYPSDCHMSKEACEMGKDLEVVDRSQGCEELYKEDKGKAVEGTFALSELDRKPAEFQAALEDPTSPEHKDLKDKLENALEASLGPGREVKIIGFKDGSIKVIFQIVLPPRSRDNPEDVKEKLMEALQDAPDLEIKPGTLMVVEAESCFGQVCEFSSECMIDPRTRDAICSCESITCDESYVPVCGDNMVTYNNDCERKQAECQEQKPIIVKYNGKCAPAREALCENVKCGFGATCTVIDGNAMCECDTVCPAEFQPVCGSDGVSYNNMCYLQAESCEKQEEIRKVDDRLCKDMECGGTRCGPYQECVDDKCQCPTVCPALYDPVCGSDGKTYDSECKLAVEACQDSLSVVVQAPGSCEFEGSGSGSGEEPEGSGDGDEEFDFCDEGTNCRFGGKCMDMDPDSEGEECVCEIKCSGDMKPVCGSDKETYDNECRLKLAACTLQKHIRVMSNGPCPDRVKVFRQQFFSDVPPVPKTDCKETTYKCCPDGKTPAAGAGFAGCPSVCKCNTLGSYARTCDSQSGQCSCKPGVGGKKCDRCEPGFWNFRGMQDGKIGCTPCECSKGGSVRDDCDQMTGKCVCKPGVSGMKCDRCRKGQVMGPTGCREPTQREPEVAESCDQLMCKYGAVCKKTSRDIAQCVCPTECKRGTIETKVCGSDGQTFADECQMRVIACRYQQDIMVAYAGPCKTGRNTTCPTNLTRTPASENATDKPSMPEGTKETVLPTESTRVPPTSASVNLVTPTKKMSTFPPLKTTEKMRPPVVSTPPPLSRTPCSSIPCMHGGTCKKMDNARSFECSCPAGWTGPVCEEEVFFYQPAFGGDSYIAFKTIKVFLSGTIMLDFRYTGKDGLLVYNGQKSGKDFISLAIVNGKLQFQYDLGSGPSEIVHPLPLEPNRWYTVSASRVKRDGYIRIGNEPDTTGASPGKTTGLNLDDDFYVGNIPPEDRERVYQRIGVTGGFKGCIRELKVVDQKGQEKMYDLRPNGKDMMFGVGVSECGSDPCKAKPCQNEGQCEETNDGNFRCVCKQGFHGPLCGHAASDPCDPDPCHPSALCVMKPKGGFLCKCPVGRRGRLCDEEIEDVWTFIPEFSGNSYIVRPGLTSVSKSLSADVVFYATKNNGMLLYNGQKTDGKGDFVSLNLKDGYLVFRFNLGKGPADIRSEDPISLNEWHEVKLDRNLRRGQMTLDGKVVGTGESPGTLSQLNLGLALFIGGGEDYSTFARDADITTGFTGAIQKLVIAGVEVPSLVDGASAAVDIRNFEKHGCTRKDNPCKNGGVCMPMMADYKCQCSVGFTGKRCDKAQADPAPLRQEGVGLDGTTILNYPNAINKR**ESNSIRE**QEPNQDDNHFEVTFRTTSDHGLLLWNHKPGGGDFIALAIVGGKPQLTYKLGKQPFTIESSITVNTGRWVTIVADSGQRLLDDLVAALGNAQPRISARSRKPPERREDNFAAKTEDQNGSTRKKHRELIEEILFHKRDTNSQKPDLVTSERRQQPPSFRVV

>Agrin isoform X1 - Strongylocentrotus purpuratus XP_030843082

MVLCIFGAVCEENEQGRPQCICDRQCPDMMAPVCGSDGTTYLSECFLDKASCEQKKRVYVASQGSCDEQDPCEGVQCSFGSECMIEGDRATCECPNACLSIYSPVCGSDGVSYGNTCEMEAASCRQQKEITLVNEGMCVEVDPCDNVTCNFGASCVVEGAVASCLCPEICLESYNPVCGSDGVDYNNECDLNAAACSQQKSVTVVFQGLCDPCQEVEYENSVCKLDRNREPQPGCDSNCPNSVINPVCGSDGVTYDNDCEINRAACLSNLEDILITFTEGPCEDGTHPCEEFTCDYGQCLVDDAGMPQCVCTPCPEVFTPVCGSDGLTHSSMCHMEEASCMERTDITLAKEGVCDGSNIIGCQTPACNKTEGAVSPVCGTDGNNYPGLCALQEAACEAGIDIQVAINGPCESCETTNCSHGSFCQMTPDGPTCTCSDHCQTINLPVCGSDGETYASECKLNVMACNARKNITVVSYGACEDCVGVTCETERFNQVCYQGMCVCQESCPMSRSDEDMVCGSDQVTYDTVCHLKMSACQAESNLTVEYYGPCDEFSGSGTEFPDYSGSGATPPNEFEFDLCDETSCSFGGICRPLSADTYECICKFNCPAVRLPVCGSDGATYGNECQLKEAACEQQSSIVLEKIGTCEDVEMEPCDGESPLVNEATMEAYTCQEDNGGEDCPSGSYCHIHPLGRFSACCAVTGEQTSQPSCVESVFGCCPDNQTEALGSNGEGCPSVCNCNPLGSYSVYCEPSNLQCPCKPGVGGKQCDRCEPGYYDFLSLERNGIGCKTCGCSSYGSRRDDCDQTSGACRCRRKAIGLKCNMCPEGLTMTPMGCMTEEEQMMTSAITCSNLTCPFSAQCQESTVEGQNATCVCPTSSSCNTDVIQVVCGDNGETYPSRCQLQVFACKEQRNIMVQNEGACETVIVTPNEIRPGCEESQFRCCPDQVTYATGPNNEGCSDASGTPPLAADQNAGQASASSPAPPSATTVALTTDAVVPSTKVSSTTLSVAPFVSTALPTDSVLPTTTIVTIVLGCDSSPCQHGGTCQNDEIAPGFRCICPLGKGGPVCNEVVTFTTPSFAGDSYLAYPEMDAFMEVEIVIEFQPGATEGVLLYEAQTAEGNGDFISLAIVNNQVEFRFLATDQSNGFDLGSAEPVVITSTVDLQLMTWHRLRAYRSRREGSLSVDGEPEVTGTSEGVSGALNLGEDLFIGYAVPPEVGLRLANTNQGFVGCIRYVEINSQELDISSSGSSVEYGANVGECGNDPCQSKEMPCFNNGLCEALNAESYRCICQGDFFGTLCGDVLVDQCEGHMCHEESTCVALPEGGYRCDCPDGRMGDMCTEEINKVVVPGFAGNSYMQLPSLMMPDDSVIDVEFLTSSPDGVIFYNGQTADGRGDFISLNMRDGYLEFRYDLGSSIAEIKSVDRLALNEWHAVRVIRMGKSGEMILNDLPPVKGTSPPGASQLNLRQPLFIGGVRSYGEVSRRAAITDGLNGAVRRFVVNEVDYSTLKDFAEAKVNVEEFREDTMNMIPTAPRKLPEMPVTTPRPPVVATRARPAMTTASVVVINTDDPLRFDGQTSIQYYNGVSKKQRALRTHQVQLSFKTAEPNGALFWNGVGNADFQAVGVSDGYVEYAYNLGRGITRIRTTQKVDNNKWHTVIITRNLIDASLQVDNEEPVMGQSRAGASQLDTDGFLYLGQGVNVPGSMESSYTYYTGCIQDVLLDEVPLHLYENAQGEKPSLFCSEP

>NP_001022152.3 Agrin - Caenorhabditis elegans

MRLHNYFVSIFLFSCIVWTVFSSCLKPRPTRSYIHSLFENSDVIIAGVITGISYRVDSLYIENITVIPRRIYKGREYVEENKNIVIGNILETEPCAHRLLASDVRIFSLTNQQGVFLLDAPMIRVSLPIFDALFTISNKQAHRKRRRTKQAICEQSLCPFGSKCGLKTGVCECKARCRVVTDVVCGSDHVSYSSFCHLSVRSCVLAKNGVRLRVATKGPCKKRNPCEDLRCGPGEDCVVNQINGILLAKCVCPTQCPNYGDSVESSPVCSSHGVDYQSSCHLRHHACESKTNITVKFFGRCDPCHGHKCPNGQTCQLGVDRRPECKCSEQCTMNSAHVCGTDGKTYLNECFLKLAACKEQKDILVWKRGNCDEAGSPCEKMECGFWGSCVVKPDRTAECECPNRCEDVMRPVCATNGETFDNECEMKKKSCETKSMIKVKHQGTCGIGVCATFDSCKKPQVCVVVDGKPKCVCPSCTDEFKEVCGSDGKTYSNECRLQNAACMAQKNIFVKYNSACEACKLKKEKCDFYSACVVGENEKAECKCPDDCPSYEMEEGKEVCGTDGVTYSSECHMKKSACHQSKFVMTAFEGKCDECLHVQCRYGEECRSGVCVCSYNCPANPPLSARICGENGVLYPSLCHLQLASCQKGAPISEMPPSHCHSSKTSFPDSCQCNRVGSFGHTCDETGQCKCRPGVAGIKCDHCLPSFWGIHLIAQGALSCRPCGCSAFGSSRSDCEQTTGKCECKNGALGDKCDLCPNGSMMTAGGCVSPAVYKTPRDCHSLRCFHGAKCVPSPSSFPDCICPQSCNMNHLGIVANMTVCGSDGTTYSNLCELKMFACKHQIDVVPVSMGICDDENFEVLDRLQREKNSNEKRLGSPCTRHEECEKLSAQCITRPGRKSVCDCDDGWKSHLGICIEISKKRKSDQLELTTDINSDWYFSRKDINRFSKLSMHIHLKEKREGILIRMVTEDKKDLQISHENRRIVIKVGKARVDSLQTIAYNVSLELKWKRNEVHFKLNGEVQKYTFEDVLDSSVKRIYVGTDGKWPRNRLNSIVGYLEIDDEPVRIDEIKPFYMAPEISRTLKFKENGFVQFENLQIDVRERTRVEIVFKPYRTNGILFYWSVPSDPHTDFIAFAMIDAKPHFVYELGSGLSYIRGEPIPLNSWHTVRIERLAKDVSMFVNETLVKKHTSQSKNAHLDISKKDALYVGFVPEGIISHKVRKLNVPFEGELQELRINELPINLISGVSKEVMDKVDVMEINEKEISKCDGASFDRKGSVVCRQHENDARGIHINEQDALVFPNNVTFSVGSSKSSNFSFDFRTLKQFGVIWQEGAWSQADEGGDFLLVFIEEGKLYVGVNLGADVHLKPISTNVTVADNHWHSVSFRRKERKCELWVDSKKILHVVASPGDVNLDSNGLVYLGGANPKKHKLLKSLNLSNKFVGCVKNLKIFGAEVNLLVDSLKSIETPKYCYN

**Agrin and Lrp4 partial sequences for AlphaFold predictions:**

>Rat Agrin **Z8 exon** NxI motif

DEKSPCQPNPCHGAAPCRVLSSGGAKCECPLGRSGTFCQTVLETAGSRPFLADFNGFSYLELKGLHTFERDLGEKMALEMVFLARGPSGLLLYNGQKTDGKGDFVSLALHNRHLEFCYDLGKGAAVIRSKEPIALGTWVRVFLERNGRKGALQVGDGPRVLGESPKSRKVPHTMLNLKEPLYIGGAPDFSKLARGAAVSSGFSGVIQLVSLRGHQLLTQEHVLRAVDVSPFADHPCTQALGNPCLNGGSCVPREATYECLCPGGFSGLHCEKGLVEKSVGDLETLAFDGRTYIEYLNAVIES**ELTNEIPA**EKALQSNHFELSLRTEATQGLVLWIGKAAERADYMALAIVDGHLQLSYDLGSQPVVLRSTVKVNTNRWLRIRAHREHREGSLQVGNEAPVTGSSPLGATQLDTDGALWLGGLQKLPVGQALPKAYGTGFVGCLRDVVVGHRQLHLLEDAVTKPELRPCPTP

>Rat Lrp4

VNECAEEGYCSQGCTNSEGAFQCWCEAGYELRPDRRSCKALGPEPVLLFANRIDIRQVLPHRSEYTLLLNNLENAIALDFHHRRELVFWSDVTLDRILRANLNGSNVEEVVSTGLESPGGLAVDWVHDKLYWTDSGTSRIEVANLDGAHRKVLLWQSLEKPRAIALHPMEGTIYWTDWGNTPRIEASSMDGSGRRIIADTHLFWPNGLTIDYAGRRMYWVDAKHHVIERANLDGSHRKAVISQGLPHPFAITVFEDSLYWTDWHTKSINSANKFTGKNQEIIRNKLHFPMDIHTLHPQRQPAGKNRCGDNNGGCTHLCLPSGQNYTCACPTGFRKINSHACA

>Ciona robusta Agrin **Z6 exon** NxF motif

KPNNPCSPNPCQGGAKCIEMPGEEEFTCKCPPGRSGSLCMTNQSAALQGPSFMPAFAGDSYLELPSLGKDVRSIMSIEILFYSNQPDGLIFYNGQKKSGKGDFVSLNLKNGFLEFKYNLGQGAANIRSANPVSLNEWHIVVLSRAMRTGDLSLDNFDPVYGTSPSQHSFLDLKQPMYVGGFPDGVKFNPEAGVTTGLSGALQKFQVNGVNLPISPAGSVSYFNVIAFNAHTCYRNPCDNGGVCHPRGAEYMCVCLPYYTGDNCEQEHSTDLLQDEQATAIYLDGTTKIMYRNAVKAM**PNDFRD**SRARTHNNYEIVFRTTARHGLLLMVGKAREGVDYIALAIHDGRLHLRFDLGSGPAHVISDQQINNGEWTTVKVNRKMNIGSLQVNNGLIKTATSPGLTNHLNSDGMLWLGGVDYRPRGIGLYKSFFVPFVGCVASAKIHDESIDLRADAFNSPVVRSCNGR

>Ciona robusta Lrp4

LNECNEEASCSQVCNNTIGSFVCSCLHGYILRPDSRTCKASGPEPALLFTNRIDIRKILPDRSEFRSILRDLENAIGLDYHIDKGLMFWTDVTLDRIMRSYVNGSDVQEVVSSGLESPGGVAVDWIHNLLYWTDSGTSRLEVSLLDGSLRKVLVWERLEKPRALALHPLHSLMFWTDWGDTPRLESAAMDGSQRHTVASRNLHWPNGLAIDYTRNHIYWADAKYHIIEKANLDGGDRRAVISRGLLHPFGVTIFEDKLYWTDWQTKSVNSANKFTGRGVKTIRSRLHFPMDIQTYHPLRQPNGHNRCEQAHCSHLCLPRPDGITCACPTGFRRVNITHCA

**Validated sgRNAs used in this study (N19):**

Nova1.2: TACAGGCCTACACGGCACA (Vitrinel et al. 2023)

Nova2.1: TAAGAATGAGCTGGCCCGG (Vitrinel et al. 2023)

Nova2.3: ATACCGGGGTACGCTGCGG (Vitrinel et al. 2023)

Agrin.1: AGGTGGCTTGTACTAACAG

Agrin.3: GTCCATAGGAATGTGCGCA

Agrin.5: TAAGAGACACTACCAGCTG

Agrin.6: GTGGTGTATGCCATCCACG

Agrin.7: CACGGTCTACTGCTCATGG

Agrin.8: CCGACCAGCAAATCAACAA

Lrp4.2: TACGTGCATACCAGTAGCT

Lrp4.4: TCACTCGCATCTCCACAGT

Ebf.C: AGACTGTGCCAAGACACCC (Gandhi et al. 2017)

**Negative control sgRNAs:**

Control: CTTTGCTACGATCTACATT (Stolfi et al. 2014)

DenhT2: CGAAATTGCTCGACGCGCT (alternate negative control sgRNA)

**Other sgRNAs designed but not used or validated:**

Nova1.1: GCTACAGCATTGTGTCTAACGG (no discernible peakshift)

Nova2.2: CTAATACCGGGGTACGCTGCGG (no discernible peakshift)

Nova2.4: CAACTTCAGAAAGATTCAGGGG (no discernible peakshift)

Nova3.1: TGATCCAAGGAACCGCCGAAGG (no discernible peakshift)

Nova3.2: ACCTTCATCAAGCCTTCGGCGG (no discernible peakshift)

Lrp4.1: GCCAAGCAGAAAATAATCCAGG (no discernible peakshift)

Lrp4.3: CAGTAGCTTGGAAATGTGATGG (peakshift 0.43)

Lrp4.5: TTGTCGCATTGAAACGAAGAGG (peakshift 0.04)

Lrp4.6: TGGCAGAATGGAAATGCGATGG (peakshift 0.15)

Agrin.2: GTGGCTTGTACTAACAGTGGGG (untested)

Agrin.4: TCCATAGGAATGTGCGCACGGG (untested)

**Peakshift PCR primers used in this study:**

Nova Exons 1b+2 Forward: TCTATGAACTGAGGTCGCAAG

Nova Exons 1b+2 Reverse: CACCTACCTAAATGGACCAAC

Lrp4 Exon 2 Forward: GTGATGTTGTCGTTTCTCATGC

Lrp4 Exon 2 Reverse: TGTTCGCACTTATTTGTAAACCG

**NGS PCR primers used in this study:**

Agrin.1+2.NGS.F: TCTAGCACAAAAGCATATCAC

Agrin.1+2.NGS.R: TCTGCTTCACAAAATTTCAGT

Agrin.3+4.NGS.F: TTCGAGAGTAAGTGGTTATGC

Agrin.3+4.NGS.R: TTTCATACAGCACTATTGACA

Agrin.5+6.NGS.F: CAACTGGACTGAGTGGTGCAT

Agrin.5+6.NGS.R: TAAGGTCTCACCTTGCTCACA

Agrin.7+8.NGS.F: GAAGCACACACACCCTAAATC

Agrin.7+8.NGS.R: TTATAGCAGGGTTTAAGCCGA

**RT-PCR primers used:**

Z11 Agrin isoform:

Ciona Agrin Z6+Z5 Forward 1: TTCGAGAGCAACTAAGTGACAA

Ciona Agrin exon41 Reverse 1: ACGAAGATGAAGACGACCGT

All Agrin isoforms:

Ciona Agrin exon 40 Forward 2: CAAGATGAACAAGCGACTGCCATT

Ciona Agrin exon 41 Reverse 2: GATATTACGTGCGCTGGACCAGAA

*Ciona Agrin* minigene assay primers:

pCI Agrin RT F: GTGTCCACTCCCAGTTCAATTACAG

pCI Agrin RT R: TGTCTGCTCGAAGCATTAACCC

Mouse *Agrin* minigene assay primers:

Mouse Agrin 31 F1: GATAGTTGAGAAGTCAGTGGGGGACC

3XFlag R: GCCGTCGTGGTCTTTGTAGTCTCTA

**Other published plasmids:**

*Fgf8/17/18>H2B::mCherry* (Stolfi and Levine 2011)

*Islet -7216/-3950 + bpFOG>Unc-76::mCherry* (Stolfi et al. 2010)

*Eef1a>Cas9* (Stolfi et al. 2014)

*Sox1/2/3>Cas9* (Stolfi et al. 2014)

*VAChT -4315/+15>Unc-76::mCherry* (Popsuj et al. 2021)

**GFP::*Ciona* Nova isoforms and GDDG/deletion mutants:**

>Wildtype “MLN” GFP Nter KH1 KH2 KH3 Cter

MVSKGEELFTGVVPILVELDGDVNGHKFSVSGEGEGDATYGKLTLKFICTTGKLPVPWPTLVTTLTYGVQCFSRYPDHMKQHDFFKSAMPEGYVQERTIFFKDDGNYKTRAEVKFEGDTLVNRIELKGIDFKEDGNILGHKLEYNYNSHNVYIMADKQKNGIKVNFKIRHNIEDGSVQLADHYQQNTPIGDGPVLLPDNHYLSTQSALSKDPNEKRDHMVLLEFVTAAGITLGMDELYKSGLRSRAQASNSMLNAMEYECQYNAGYSIVSNGNEYGLIQAYTAHDYPLENGVTFSAPPPGQLILKVLIPGYAAGAVIGKGGQIIVQLQKDSGAIIKLSKAKDFYPGTQDRVVLIQGTAEGLMKVQNTIIEKVYEFPVPKDLAAIIGDRPKQVKIIVPNTTAGLVIGKAGATIKTIMEESGSKVQLSQKPDGVNVQERVITIKGEKHQLMTASNIIIDKIKDDPQSASCPHISYSGIAGPIANANPTGSPYAAGSAALVDASHPSVAAMLGHYVIPGQQVLQTAMPLSHHPHQSALSSGSVTPAPELTTINHAMTTLANYGYTLGGVNYGTLGVMPSVHPSVHPGIATSVGMISAGSLAGSPIPSATPLLSATALPTESSIPTAVPTAQAISMQSNYLANLANAGYLTTGHPQLLGATSGLGGLTTVSQHPPPAATPTSFSVASTPSTPGLPVSFSPHSTVSILSIEKSSDGQKETIELAIPENLIGAVLGKAGRTLVEYQDVSGAKIQISKKGDYVAGTRNRRVTITGKPPCPQTAQFLITQRVASAQNARAQQAKLL*

>Wildtype “MMM” GFP Nter KH1 KH2 KH3 Cter

MVSKGEELFTGVVPILVELDGDVNGHKFSVSGEGEGDATYGKLTLKFICTTGKLPVPWPTLVTTLTYGVQCFSRYPDHMKQHDFFKSAMPEGYVQERTIFFKDDGNYKTRAEVKFEGDTLVNRIELKGIDFKEDGNILGHKLEYNYNSHNVYIMADKQKNGIKVNFKIRHNIEDGSVQLADHYQQNTPIGDGPVLLPDNHYLSTQSALSKDPNEKRDHMVLLEFVTAAGITLGMDELYKSGLRSRAQASNSMMMTAVVPMPNGTYLIESRKRPLEEPIELVDFKRERSEMEDYPLENGVTFSAPPPGQLILKVLIPGYAAGAVIGKGGQIIVQLQKDSGAIIKLSKAKDFYPGTQDRVVLIQGTAEGLMKVQNTIIEKVYEFPVPKDLAAIIGDRPKQVKIIVPNTTAGLVIGKAGATIKTIMEESGSKVQLSQKPDGVNVQERVITIKGEKHQLMTASNIIIDKIKDDPQSASCPHISYSGIAGPIANANPTGSPYAAGSAALVDASHPSVAAMLGHYVIPGQQVLQTAMPLSHHPHQSALSSGSVTPAPELTTINHAMTTLANYGYTLGGVNYGTLGVMPSVHPSVHPGIATSVGMISAGSLAGSPIPSATPLLSATALPTESSIPTAVPTAQAISMQSNYLANLANAGYLTTGHPQLLGATSGLGGLTTVSQHPPPAATPTSFSVASTPSTPGLPVSFSPHSTVSILSIEKSSDGQKETIELAIPENLIGAVLGKAGRTLVEYQDVSGAKIQISKKGDYVAGTRNRRVTITGKPPCPQTAQFLITQRVASAQNARAQQAKLL*

>Wildtype “MEY” GFP Nter KH1 KH2 KH3 Cter

MVSKGEELFTGVVPILVELDGDVNGHKFSVSGEGEGDATYGKLTLKFICTTGKLPVPWPTLVTTLTYGVQCFSRYPDHMKQHDFFKSAMPEGYVQERTIFFKDDGNYKTRAEVKFEGDTLVNRIELKGIDFKEDGNILGHKLEYNYNSHNVYIMADKQKNGIKVNFKIRHNIEDGSVQLADHYQQNTPIGDGPVLLPDNHYLSTQSALSKDPNEKRDHMVLLEFVTAAGITLGMDELYKSGLRSRAQASNSMEYECQYNAGYSIVSNGNEYGLIQAYTAHDYPLENGVTFSAPPPGQLILKVLIPGYAAGAVIGKGGQIIVQLQKDSGAIIKLSKAKDFYPGTQDRVVLIQGTAEGLMKVQNTIIEKVYEFPVPKDLAAIIGDRPKQVKIIVPNTTAGLVIGKAGATIKTIMEESGSKVQLSQKPDGVNVQERVITIKGEKHQLMTASNIIIDKIKDDPQSASCPHISYSGIAGPIANANPTGSPYAAGSAALVDASHPSVAAMLGHYVIPGQQVLQTAMPLSHHPHQSALSSGSVTPAPELTTINHAMTTLANYGYTLGGVNYGTLGVMPSVHPSVHPGIATSVGMISAGSLAGSPIPSATPLLSATALPTESSIPTAVPTAQAISMQSNYLANLANAGYLTTGHPQLLGATSGLGGLTTVSQHPPPAATPTSFSVASTPSTPGLPVSFSPHSTVSILSIEKSSDGQKETIELAIPENLIGAVLGKAGRTLVEYQDVSGAKIQISKKGDYVAGTRNRRVTITGKPPCPQTAQFLITQRVASAQNARAQQAKLL*

>”MLN” mKH1 **GDDG** GFP Nter KH1 KH2 KH3 Cter

MVSKGEELFTGVVPILVELDGDVNGHKFSVSGEGEGDATYGKLTLKFICTTGKLPVPWPTLVTTLTYGVQCFSRYPDHMKQHDFFKSAMPEGYVQERTIFFKDDGNYKTRAEVKFEGDTLVNRIELKGIDFKEDGNILGHKLEYNYNSHNVYIMADKQKNGIKVNFKIRHNIEDGSVQLADHYQQNTPIGDGPVLLPDNHYLSTQSALSKDPNEKRDHMVLLEFVTAAGITLGMDELYKSGLRSRAQASNSMLNAMEYECQYNAGYSIVSNGNEYGLIQAYTAHDYPLENGVTFSAPPPGQLILKVLIPGYAAGAVI**GDDG**QIIVQLQKDSGAIIKLSKAKDFYPGTQDRVVLIQGTAEGLMKVQNTIIEKVYEFPVPKDLAAIIGDRPKQVKIIVPNTTAGLVIGKAGATIKTIMEESGSKVQLSQKPDGVNVQERVITIKGEKHQLMTASNIIIDKIKDDPQSASCPHISYSGIAGPIANANPTGSPYAAGSAALVDASHPSVAAMLGHYVIPGQQVLQTAMPLSHHPHQSALSSGSVTPAPELTTINHAMTTLANYGYTLGGVNYGTLGVMPSVHPSVHPGIATSVGMISAGSLAGSPIPSATPLLSATALPTESSIPTAVPTAQAISMQSNYLANLANAGYLTTGHPQLLGATSGLGGLTTVSQHPPPAATPTSFSVASTPSTPGLPVSFSPHSTVSILSIEKSSDGQKETIELAIPENLIGAVLGKAGRTLVEYQDVSGAKIQISKKGDYVAGTRNRRVTITGKPPCPQTAQFLITQRVASAQNARAQQAKLL*

>”MLN” mKH2 **GDDG** GFP Nter KH1 KH2 KH3 Cter

MVSKGEELFTGVVPILVELDGDVNGHKFSVSGEGEGDATYGKLTLKFICTTGKLPVPWPTLVTTLTYGVQCFSRYPDHMKQHDFFKSAMPEGYVQERTIFFKDDGNYKTRAEVKFEGDTLVNRIELKGIDFKEDGNILGHKLEYNYNSHNVYIMADKQKNGIKVNFKIRHNIEDGSVQLADHYQQNTPIGDGPVLLPDNHYLSTQSALSKDPNEKRDHMVLLEFVTAAGITLGMDELYKSGLRSRAQASNSMLNAMEYECQYNAGYSIVSNGNEYGLIQAYTAHDYPLENGVTFSAPPPGQLILKVLIPGYAAGAVIGKGGQIIVQLQKDSGAIIKLSKAKDFYPGTQDRVVLIQGTAEGLMKVQNTIIEKVYEFPVPKDLAAIIGDRPKQVKIIVPNTTAGLVI**GDDG**ATIKTIMEESGSKVQLSQKPDGVNVQERVITIKGEKHQLMTASNIIIDKIKDDPQSASCPHISYSGIAGPIANANPTGSPYAAGSAALVDASHPSVAAMLGHYVIPGQQVLQTAMPLSHHPHQSALSSGSVTPAPELTTINHAMTTLANYGYTLGGVNYGTLGVMPSVHPSVHPGIATSVGMISAGSLAGSPIPSATPLLSATALPTESSIPTAVPTAQAISMQSNYLANLANAGYLTTGHPQLLGATSGLGGLTTVSQHPPPAATPTSFSVASTPSTPGLPVSFSPHSTVSILSIEKSSDGQKETIELAIPENLIGAVLGKAGRTLVEYQDVSGAKIQISKKGDYVAGTRNRRVTITGKPPCPQTAQFLITQRVASAQNARAQQAKLL*

>“MLN” mKH3 **GDDG** GFP Nter KH1 KH2 KH3 Cter

MVSKGEELFTGVVPILVELDGDVNGHKFSVSGEGEGDATYGKLTLKFICTTGKLPVPWPTLVTTLTYGVQCFSRYPDHMKQHDFFKSAMPEGYVQERTIFFKDDGNYKTRAEVKFEGDTLVNRIELKGIDFKEDGNILGHKLEYNYNSHNVYIMADKQKNGIKVNFKIRHNIEDGSVQLADHYQQNTPIGDGPVLLPDNHYLSTQSALSKDPNEKRDHMVLLEFVTAAGITLGMDELYKSGLRSRAQASNSMLNAMEYECQYNAGYSIVSNGNEYGLIQAYTAHDYPLENGVTFSAPPPGQLILKVLIPGYAAGAVIGKGGQIIVQLQKDSGAIIKLSKAKDFYPGTQDRVVLIQGTAEGLMKVQNTIIEKVYEFPVPKDLAAIIGDRPKQVKIIVPNTTAGLVIGKAGATIKTIMEESGSKVQLSQKPDGVNVQERVITIKGEKHQLMTASNIIIDKIKDDPQSASCPHISYSGIAGPIANANPTGSPYAAGSAALVDASHPSVAAMLGHYVIPGQQVLQTAMPLSHHPHQSALSSGSVTPAPELTTINHAMTTLANYGYTLGGVNYGTLGVMPSVHPSVHPGIATSVGMISAGSLAGSPIPSATPLLSATALPTESSIPTAVPTAQAISMQSNYLANLANAGYLTTGHPQLLGATSGLGGLTTVSQHPPPAATPTSFSVASTPSTPGLPVSFSPHSTVSILSIEKSSDGQKETIELAIPENLIGAVL**GDDG**RTLVEYQDVSGAKIQISKKGDYVAGTRNRRVTITGKPPCPQTAQFLITQRVASAQNARAQQAKLL*

>“MLN” Delta Nter GFP KH1 KH2 KH3 Cter

MVSKGEELFTGVVPILVELDGDVNGHKFSVSGEGEGDATYGKLTLKFICTTGKLPVPWPTLVTTLTYGVQCFSRYPDHMKQHDFFKSAMPEGYVQERTIFFKDDGNYKTRAEVKFEGDTLVNRIELKGIDFKEDGNILGHKLEYNYNSHNVYIMADKQKNGIKVNFKIRHNIEDGSVQLADHYQQNTPIGDGPVLLPDNHYLSTQSALSKDPNEKRDHMVLLEFVTAAGITLGMDELYKSGLRSRAQASNSMILKVLIPGYAAGAVIGKGGQIIVQLQKDSGAIIKLSKAKDFYPGTQDRVVLIQGTAEGLMKVQNTIIEKVYEFPVPKDLAAIIGDRPKQVKIIVPNTTAGLVIGKAGATIKTIMEESGSKVQLSQKPDGVNVQERVITIKGEKHQLMTASNIIIDKIKDDPQSASCPHISYSGIAGPIANANPTGSPYAAGSAALVDASHPSVAAMLGHYVIPGQQVLQTAMPLSHHPHQSALSSGSVTPAPELTTINHAMTTLANYGYTLGGVNYGTLGVMPSVHPSVHPGIATSVGMISAGSLAGSPIPSATPLLSATALPTESSIPTAVPTAQAISMQSNYLANLANAGYLTTGHPQLLGATSGLGGLTTVSQHPPPAATPTSFSVASTPSTPGLPVSFSPHSTVSILSIEKSSDGQKETIELAIPENLIGAVLGKAGRTLVEYQDVSGAKIQISKKGDYVAGTRNRRVTITGKPPCPQTAQFLITQRVASAQNARAQQAKLL*

>“MLN” Delta Cter GFP Nter KH1 KH2 KH3

MVSKGEELFTGVVPILVELDGDVNGHKFSVSGEGEGDATYGKLTLKFICTTGKLPVPWPTLVTTLTYGVQCFSRYPDHMKQHDFFKSAMPEGYVQERTIFFKDDGNYKTRAEVKFEGDTLVNRIELKGIDFKEDGNILGHKLEYNYNSHNVYIMADKQKNGIKVNFKIRHNIEDGSVQLADHYQQNTPIGDGPVLLPDNHYLSTQSALSKDPNEKRDHMVLLEFVTAAGITLGMDELYKSGLRSRAQASNSMLNAMEYECQYNAGYSIVSNGNEYGLIQAYTAHDYPLENGVTFSAPPPGQLILKVLIPGYAAGAVIGKGGQIIVQLQKDSGAIIKLSKAKDFYPGTQDRVVLIQGTAEGLMKVQNTIIEKVYEFPVPKDLAAIIGDRPKQVKIIVPNTTAGLVIGKAGATIKTIMEESGSKVQLSQKPDGVNVQERVITIKGEKHQLMTASNIIIDKIKDDPQSASCPHISYSGIAGPIANANPTGSPYAAGSAALVDASHPSVAAMLGHYVIPGQQVLQTAMPLSHHPHQSALSSGSVTPAPELTTINHAMTTLANYGYTLGGVNYGTLGVMPSVHPSVHPGIATSVGMISAGSLAGSPIPSATPLLSATALPTESSIPTAVPTAQAISMQSNYLANLANAGYLTTGHPQLLGATSGLGGLTTVSQHPPPAATPTSFSVASTPSTPGLPVSFSPHSTVSILSIEKSSDGQKETIELAIPENLIGAVLGKAGRTLVEYQDVSGAKIQISKKGDYVAGTRNRRVTITGKPPCPQTAQFLIT*

>“MLN” Delta Nter+Cter GFP KH1 KH2 KH3

MVSKGEELFTGVVPILVELDGDVNGHKFSVSGEGEGDATYGKLTLKFICTTGKLPVPWPTLVTTLTYGVQCFSRYPDHMKQHDFFKSAMPEGYVQERTIFFKDDGNYKTRAEVKFEGDTLVNRIELKGIDFKEDGNILGHKLEYNYNSHNVYIMADKQKNGIKVNFKIRHNIEDGSVQLADHYQQNTPIGDGPVLLPDNHYLSTQSALSKDPNEKRDHMVLLEFVTAAGITLGMDELYKSGLRSRAQASNSMILKVLIPGYAAGAVIGKGGQIIVQLQKDSGAIIKLSKAKDFYPGTQDRVVLIQGTAEGLMKVQNTIIEKVYEFPVPKDLAAIIGDRPKQVKIIVPNTTAGLVIGKAGATIKTIMEESGSKVQLSQKPDGVNVQERVITIKGEKHQLMTASNIIIDKIKDDPQSASCPHISYSGIAGPIANANPTGSPYAAGSAALVDASHPSVAAMLGHYVIPGQQVLQTAMPLSHHPHQSALSSGSVTPAPELTTINHAMTTLANYGYTLGGVNYGTLGVMPSVHPSVHPGIATSVGMISAGSLAGSPIPSATPLLSATALPTESSIPTAVPTAQAISMQSNYLANLANAGYLTTGHPQLLGATSGLGGLTTVSQHPPPAATPTSFSVASTPSTPGLPVSFSPHSTVSILSIEKSSDGQKETIELAIPENLIGAVLGKAGRTLVEYQDVSGAKIQISKKGDYVAGTRNRRVTITGKPPCPQTAQFLIT*

>”MLN” Delta KH3 GFP Nter KH1 KH2 Cter

MVSKGEELFTGVVPILVELDGDVNGHKFSVSGEGEGDATYGKLTLKFICTTGKLPVPWPTLVTTLTYGVQCFSRYPDHMKQHDFFKSAMPEGYVQERTIFFKDDGNYKTRAEVKFEGDTLVNRIELKGIDFKEDGNILGHKLEYNYNSHNVYIMADKQKNGIKVNFKIRHNIEDGSVQLADHYQQNTPIGDGPVLLPDNHYLSTQSALSKDPNEKRDHMVLLEFVTAAGITLGMDELYKSGLRSRAQASNSMLNAMEYECQYNAGYSIVSNGNEYGLIQAYTAHDYPLENGVTFSAPPPGQLILKVLIPGYAAGAVIGKGGQIIVQLQKDSGAIIKLSKAKDFYPGTQDRVVLIQGTAEGLMKVQNTIIEKVYEFPVPKDLAAIIGDRPKQVKIIVPNTTAGLVIGKAGATIKTIMEESGSKVQLSQKPDGVNVQERVITIKGEKHQLMTASNIIIDKIKDDPQSASCPHISYSGIAGPIANANPTGSPYAAGSAALVDASHPSVAAMLGHYVIPGQQVLQTAMPLSHHPHQSALSSGSVTPAPELTTINHAMTTLANYGYTLGGVNYGTLGVMPSVHPSVHPGIATSVGMISAGSLAGSPIPSATPLLSATALPTESSIPTAVPTAQAISMQSNYLANLANAGYLTTGHPQLLGATSGLGGLTTVSQHPPPAATPTSFSVASTPSTPGLPVSFSPHSTVSILSIEKSSDGQKEQRVASAQNARAQQAKLL*

>”MLN” Delta Nter+KH3 GFP KH1 KH2 Cter

MVSKGEELFTGVVPILVELDGDVNGHKFSVSGEGEGDATYGKLTLKFICTTGKLPVPWPTLVTTLTYGVQCFSRYPDHMKQHDFFKSAMPEGYVQERTIFFKDDGNYKTRAEVKFEGDTLVNRIELKGIDFKEDGNILGHKLEYNYNSHNVYIMADKQKNGIKVNFKIRHNIEDGSVQLADHYQQNTPIGDGPVLLPDNHYLSTQSALSKDPNEKRDHMVLLEFVTAAGITLGMDELYKSGLRSRAQASNSMILKVLIPGYAAGAVIGKGGQIIVQLQKDSGAIIKLSKAKDFYPGTQDRVVLIQGTAEGLMKVQNTIIEKVYEFPVPKDLAAIIGDRPKQVKIIVPNTTAGLVIGKAGATIKTIMEESGSKVQLSQKPDGVNVQERVITIKGEKHQLMTASNIIIDKIKDDPQSASCPHISYSGIAGPIANANPTGSPYAAGSAALVDASHPSVAAMLGHYVIPGQQVLQTAMPLSHHPHQSALSSGSVTPAPELTTINHAMTTLANYGYTLGGVNYGTLGVMPSVHPSVHPGIATSVGMISAGSLAGSPIPSATPLLSATALPTESSIPTAVPTAQAISMQSNYLANLANAGYLTTGHPQLLGATSGLGGLTTVSQHPPPAATPTSFSVASTPSTPGLPVSFSPHSTVSILSIEKSSDGQKEQRVASAQNARAQQAKLL*

>”MLN” Delta KH3+Cter GFP Nter KH1 KH2

MVSKGEELFTGVVPILVELDGDVNGHKFSVSGEGEGDATYGKLTLKFICTTGKLPVPWPTLVTTLTYGVQCFSRYPDHMKQHDFFKSAMPEGYVQERTIFFKDDGNYKTRAEVKFEGDTLVNRIELKGIDFKEDGNILGHKLEYNYNSHNVYIMADKQKNGIKVNFKIRHNIEDGSVQLADHYQQNTPIGDGPVLLPDNHYLSTQSALSKDPNEKRDHMVLLEFVTAAGITLGMDELYKSGLRSRAQASNSMLNAMEYECQYNAGYSIVSNGNEYGLIQAYTAHDYPLENGVTFSAPPPGQLILKVLIPGYAAGAVIGKGGQIIVQLQKDSGAIIKLSKAKDFYPGTQDRVVLIQGTAEGLMKVQNTIIEKVYEFPVPKDLAAIIGDRPKQVKIIVPNTTAGLVIGKAGATIKTIMEESGSKVQLSQKPDGVNVQERVITIKGEKHQLMTASNIIIDKIKDDPQSASCPHISYSGIAGPIANANPTGSPYAAGSAALVDASHPSVAAMLGHYVIPGQQVLQTAMPLSHHPHQSALSSGSVTPAPELTTINHAMTTLANYGYTLGGVNYGTLGVMPSVHPSVHPGIATSVGMISAGSLAGSPIPSATPLLSATALPTESSIPTAVPTAQAISMQSNYLANLANAGYLTTGHPQLLGATSGLGGLTTVSQHPPPAATPTSFSVASTPSTPGLPVSFSPHSTVSILSIEKSS*

>”MLN” Delta Nter+KH3+Cter GFP KH1 KH2

MVSKGEELFTGVVPILVELDGDVNGHKFSVSGEGEGDATYGKLTLKFICTTGKLPVPWPTLVTTLTYGVQCFSRYPDHMKQHDFFKSAMPEGYVQERTIFFKDDGNYKTRAEVKFEGDTLVNRIELKGIDFKEDGNILGHKLEYNYNSHNVYIMADKQKNGIKVNFKIRHNIEDGSVQLADHYQQNTPIGDGPVLLPDNHYLSTQSALSKDPNEKRDHMVLLEFVTAAGITLGMDELYKSGLRSRAQASNSMILKVLIPGYAAGAVIGKGGQIIVQLQKDSGAIIKLSKAKDFYPGTQDRVVLIQGTAEGLMKVQNTIIEKVYEFPVPKDLAAIIGDRPKQVKIIVPNTTAGLVIGKAGATIKTIMEESGSKVQLSQKPDGVNVQERVITIKGEKHQLMTASNIIIDKIKDDPQSASCPHISYSGIAGPIANANPTGSPYAAGSAALVDASHPSVAAMLGHYVIPGQQVLQTAMPLSHHPHQSALSSGSVTPAPELTTINHAMTTLANYGYTLGGVNYGTLGVMPSVHPSVHPGIATSVGMISAGSLAGSPIPSATPLLSATALPTESSIPTAVPTAQAISMQSNYLANLANAGYLTTGHPQLLGATSGLGGLTTVSQHPPPAATPTSFSVASTPSTPGLPVSFSPHSTVSILSIEKSS*

Note: GDDG/deletion mutant “MMM” constructs carry the same mutations or deletions as the MLN constructs listed above. Different KH domain GDDG mutation permutations can be inferred by combining the individual GDDG mutations. Also, deletion of the N-terminus result in the same construct for both MMM and MLN isoforms, since the isoforms differ only in their N-termini.

>GFP::Mouse Nova1 GFP Nova1 GXGG motifs in each KH domain

MVSKGEELFTGVVPILVELDGDVNGHKFSVSGEGEGDATYGKLTLKFICTTGKLPVPWPTLVTTLTYGVQCFSRYPDHMKQHDFFKSAMPEGYVQERTIFFKDDGNYKTRAEVKFEGDTLVNRIELKGIDFKEDGNILGHKLEYNYNSHNVYIMADKQKNGIKVNFKIRHNIEDGSVQLADHYQQNTPIGDGPVLLPDNHYLSTQSALSKDPNEKRDHMVLLEFVTAAGITLGMDELYKSGLRSRAQASMMAAAPIQQNGTHTGVPIDLDPPDSRKRPLEAPPEAGSTKRTNTGEDGQYFLKVLIPSYAAGSIIGKGGQTIVQLQKETGATIKLSKSKDFYPGTTERVCLIQGTIEALNAVHGFIAEKIREMPQNVAKTEPVSILQPQTTVNPDRIKQTLPSSPTTTKSSPSDPMTTSRANQVKIIVPNSTAGLIIGKGGATVKAIMEQSGAWVQLSQKPDGINLQERVVTVSGEPEQNRKAVELIIQKIQEDPQSGSCLNISYANVTGPVANSNPTGSPYANTAEVLPTAAAAAGLLGHANLAGVAAFPAVLSGFTGNDLVAITSALNTLASYGYNLNTLGLGLSQAAATGALAAAAASANPAAAAANLLATYASEASASGSTAGGTAGTFALGSLAAATAATNGYFGAASPLAASAILGTEKSTDGSKDVVEIAVPENLVGAILGKGGKTLVEYQELTGARIQISKKGEFVPGTRNRKVTITGTPAATQAAQYLITQRITYEQGVRAANPQKVG*

>GFP::MouseNova2 GFP Nova2 GXGG motifs in each KH domain

MVSKGEELFTGVVPILVELDGDVNGHKFSVSGEGEGDATYGKLTLKFICTTGKLPVPWPTLVTTLTYGVQCFSRYPDHMKQHDFFKSAMPEGYVQERTIFFKDDGNYKTRAEVKFEGDTLVNRIELKGIDFKEDGNILGHKLEYNYNSHNVYIMADKQKNGIKVNFKIRHNIEDGSVQLADHYQQNTPIGDGPVLLPDNHYLSTQSALSKDPNEKRDHMVLLEFVTAAGITLGMDELYKSGLRSRAQASNSMEPEAPDSRKRPLETPPEVVCTKRSNTGEEGEYFLKVLIPSYAAGSIIGKGGQTIVQLQKETGATIKLSKSKDFYPGTTERVCLVQGTAEALNAVHSFIAEKVREIPQAMTKPEVVNILQPQTTMNPDRAKQAKLIVPNSTAGLIIGKGGATVKAVMEQSGAWVQLSQKSEGINLQERVVTVSGEPEQVHKAVSAIVQKVQEDPQSSSCLNISYANVAGPVANSNPTGSPYASPADVLPAAAAASAAAASGLLGPAGLAGVGAFPAALPAFSGTDLLAISTALNTLASYGYNTNSLSLGLNSAAASGVLAAVAAGANPAAAAAANLLASYAGDAGAGPGAGAAPPPPPPPGALGSFALAAAANGYLGAGAGGAAGAGGAPLVAAAAAAGAAGGFLTAEKLAAESAKELVEIAVPENLVGAILGKGGKTLVEYQELTGARIQISKKGEFLPGTRNRRVTITGSPAATQAAQYLISQRVTYEQGVRASNPQKVG*

**New construct based on new combinations of published sequences:**

*Tbx6-related.b>AChRA1::GFP*

Tbx6-related.b driver (Christiaen et al. 2009)

AChRA1::GFP (Nishino et al. 2011)

ggcgcgcccaacggagtacgcgtgtcaagtttaatggcgtaattaccgaacaactgttgataagtaatgaggaccccgctgcggtaacctttcgaaattgctggttgcaagcggtgttggtgcgataataaggaaaagcggtaagcgttattttttgatccaccacaaagaccaaacctatattgatacactaaactaaatcaaattcgaacctatccattaaagtgcgaaatatacatagaatgaacaataatccgagctatattatgggcaatattttggctagttttctgcttacaataatacataagaggcctacactaaatacggcggtatataaaactatgcaagacatacccatatattagttttaatcaaattgatttaaaatatttttaaaactattaggcaactttgagtagaataagggtttaacctaaacgtatgtggttttagccatcgatggaaacgttttacaattatcgttacttttttgaagaccttttcgttctatttttaagaagaacattcaaagaaatggaaaaccgttttcttacgaatcctatataccgttgttaattgtttaaaaacacgataaggatattatgttctgaatgtgtcccatcttaccccacagcactatataataaaattttagtttttgtgacagacttatgtcgcattcttaaacggatgatccccaactggttaacaacgtaacaagcttgcaaaagcgaaggtgacattggtaacaacgtacgataactattgtctactaataacaatagacttaacacataacatatttaggaaatggcttcatatggcggactgtcaacttagttgtcaaaattacctttaaatctctataaacgaagctgtttaataaaaaaaactaacaaggctattctacatcaaaccataataatgaaattatcaaacacatttttaaagcgatttcatcaaaccaacgcgccacatgcaagacggtatgcgtcacactgagttttggagtgttctgcatgcgctagacttgaatcagcaggagagttcggaggcttatcaggaagcagttgtccttgttaatgactcgttaagatcaaagtggcatcgaaaacgagtctcgctataaaacgggtttagtttcacagtacctcattccgctttctgttctcattggatatacaccaaactgaaagtacgagttaaatcgaaagagagaggaaattgtaagttttattggaccagacaagactatggcgaatatggcggccgcaaccATGatagttttgcgtttactggttatgggtgcgcttgcgtatgtgagcgtggcaaaaacccggtacgatttatcaggagatataatgcaggggtatgacgctaaagtacgaccgagtgacagctacaataactcggtaaaagtcgtgtttaagcttgtgtttaatcaactactagacgtgagcgaagtcaaccaaaaaatcgaaacgaaactgtgggtttatcacaagtggatggaccctcggttaagctgggtgccagaggattacgaaaacctggagtatatatatctacctactaccaacttgtggctgccggagttggttctgtataacaacgccgatggtgactttgctatttctcaatttacgaaagcaaaagtggattatactggaatggtagaatggaaacctccggcaatttttaaaagcttctgcgagattatggtggcagagtttccatttgacacacaaaactgtacgatgaaaattggcccttggtcgcaaggacaagatttactggacatggtaaattcagactgggaagtcaaagaccacatgtgcgagcccccagatgaaacaatgtacgaggaaagcggagaatggttgattcttaaaactggttgctggaagcattacattaaatacgattgctgtcgagggccgtacgtggacatgacttactacttcatattgcaaagacggcctctatatcttgttatcaacattctctttcctacaatgctgttttcgtatctaacctgcgctgtattctatctgccatcggacgctggcgaaaaaataacactcagtatttcgcttctgctttcactgattgtgttcttgctcgttattgttgaagcgattccctcaaccgctaatggagtcccattgctatgccagtacattctatttactatgatattggtttgcctctctattatgataacagtcggtgtattgaatgtacattaccgtgggcctgcaacacatgtcatgtcggatcggatgaaaaagatattcatggtgtggcttccaaaattcatctatagctcgacaatgaaacgattggatccttacaaggaagaaaaaatgttggctggcagacctcccaaaccttacaaagatatttcagatttatctggtcgGGCCCCCATGGTGAGCAAGGGCGAGGAGCTGTTCACCGGGGTGGTGCCCATCCTGGTCGAGCTGGACGGCGACGTAAACGGCCACAAGTTCAGCGTGTCCGGCGAGGGCGAGGGCGATGCCACCTACGGCAAGCTGACCCTGAAGTTCATCTGCACCACCGGCAAGCTGCCCGTGCCCTGGCCCACCCTCGTGACCACCCTGACCTACGGCGTGCAGTGCTTCAGCCGCTACCCCGACCACATGAAGCAGCACGACTTCTTCAAGTCCGCCATGCCCGAAGGCTACGTCCAGGAGCGCACCATCTTCTTCAAGGACGACGGCAACTACAAGACCCGCGCCGAGGTGAAGTTCGAGGGCGACACCCTGGTGAACCGCATCGAGCTGAAGGGCATCGACTTCAAGGAGGACGGCAACATCCTGGGGCACAAGCTGGAGTACAACTACAACAGCCACAACGTCTATATCATGGCCGACAAGCAGAAGAACGGCATCAAGGTGAACTTCAAGATCCGCCACAACATCGAGGACGGCAGCGTGCAGCTCGCCGACCACTACCAGCAGAACACCCCCATCGGCGACGGCCCCGTGCTGCTGCCCGACAACCACTACCTGAGCACCCAGTCCGCCCTGAGCAAAGACCCCAACGAGAAGCGCGATCACATGGTCCTGCTGGAGTTCGTGACCGCCGCCGGGATCACTCTCGGCATGGACGAGCTGTACAAGCGttccccaaaaccagacgaaccacatttaattggtagcgacgttaaaacagctatggacggagtagattatgtgtcagagtgttataaggaccaacgcgaaggacagcagaaagaagacgaatggaaatacgtcgctatggtgttggaccattttctgttgtatatctttatactggcttgcgtggttggcacagtcggtatatttggcaagcggttgcttgaatttatgtcagaacaagagctttttaaaaacctaggcaacgagtgcattctgaaatgccaaagtgattaAAGCgaattc

*Tbx6-related.b>Cas9*

Tbx6-related.b driver (Christiaen et al. 2009)

Cas9 (Stolfi et al. 2014

ggcgcgcccaacggagtacgcgtgtcaagtttaatggcgtaattaccgaacaactgttgataagtaatgaggaccccgctgcggtaacctttcgaaattgctggttgcaagcggtgttggtgcgataataaggaaaagcggtaagcgttattttttgatccaccacaaagaccaaacctatattgatacactaaactaaatcaaattcgaacctatccattaaagtgcgaaatatacatagaatgaacaataatccgagctatattatgggcaatattttggctagttttctgcttacaataatacataagaggcctacactaaatacggcggtatataaaactatgcaagacatacccatatattagttttaatcaaattgatttaaaatatttttaaaactattaggcaactttgagtagaataagggtttaacctaaacgtatgtggttttagccatcgatggaaacgttttacaattatcgttacttttttgaagaccttttcgttctatttttaagaagaacattcaaagaaatggaaaaccgttttcttacgaatcctatataccgttgttaattgtttaaaaacacgataaggatattatgttctgaatgtgtcccatcttaccccacagcactatataataaaattttagtttttgtgacagacttatgtcgcattcttaaacggatgatccccaactggttaacaacgtaacaagcttgcaaaagcgaaggtgacattggtaacaacgtacgataactattgtctactaataacaatagacttaacacataacatatttaggaaatggcttcatatggcggactgtcaacttagttgtcaaaattacctttaaatctctataaacgaagctgtttaataaaaaaaactaacaaggctattctacatcaaaccataataatgaaattatcaaacacatttttaaagcgatttcatcaaaccaacgcgccacatgcaagacggtatgcgtcacactgagttttggagtgttctgcatgcgctagacttgaatcagcaggagagttcggaggcttatcaggaagcagttgtccttgttaatgactcgttaagatcaaagtggcatcgaaaacgagtctcgctataaaacgggtttagtttcacagtacctcattccgctttctgttctcattggatatacaccaaactgaaagtacgagttaaatcgaaagagagaggaaattgtaagttttattggaccagacaagactatggcgaatatggcggccgcaaccATGGCTAGCCCCAAAAAGAAGAGGAAAGTGGACAAGAAGTATTCTATCGGACTGGACATCGGGACTAATAGCGTCGGGTGGGCCGTGATCACTGACGAGTACAAGGTGCCCTCTAAGAAGTTCAAGGTGCTCGGGAACACCGACCGGCATTCCATCAAGAAAAATCTGATCGGAGCTCTCCTCTTTGATTCAGGGGAGACCGCTGAAGCAACCCGCCTCAAGCGGACTGCTAGACGGCGGTACACCAGGAGGAAGAACCGGATTTGTTACCTTCAAGAGATATTCTCCAACGAAATGGCAAAGGTCGACGACAGCTTCTTCCATAGGCTGGAAGAATCATTCCTCGTGGAAGAGGATAAGAAGCATGAACGGCATCCCATCTTCGGTAATATCGTCGACGAGGTGGCCTATCACGAGAAATACCCAACCATCTACCATCTTCGCAAAAAGCTGGTGGACTCAACCGACAAGGCAGACCTCCGGCTTATCTACCTGGCCCTGGCCCACATGATCAAGTTCAGAGGCCACTTCCTGATCGAGGGCGACCTCAATCCTGACAATAGCGATGTGGATAAACTGTTCATCCAGCTGGTGCAGACTTACAACCAGCTCTTTGAAGAGAACCCCATCAATGCAAGCGGAGTCGATGCCAAGGCCATTCTGTCAGCCCGGCTGTCAAAGAGCCGCAGACTTGAGAATCTTATCGCTCAGCTGCCGGGTGAAAAGAAAAATGGACTGTTCGGGAACCTGATTGCTCTTTCACTTGGGCTGACTCCCAATTTCAAGTCTAATTTCGACCTGGCAGAGGATGCCAAGCTGCAACTGTCCAAGGACACCTATGATGACGATCTCGACAACCTCCTGGCCCAGATCGGTGACCAATACGCCGACCTTTTCCTTGCTGCTAAGAATCTTTCTGACGCCATCCTGCTGTCTGACATTCTCCGCGTGAACACTGAAATCACCAAGGCCCCTCTTTCAGCTTCAATGATTAAGCGGTATGATGAGCACCACCAGGACCTGACCCTGCTTAAGGCACTCGTCCGGCAGCAGCTTCCGGAGAAGTACAAGGAAATCTTCTTTGACCAGTCAAAGAATGGATACGCCGGCTACATCGACGGAGGTGCCTCCCAAGAGGAATTTTATAAGTTTATCAAACCTATCCTTGAGAAGATGGACGGCACCGAAGAGCTCCTCGTGAAACTGAATCGGGAGGATCTGCTGCGGAAGCAGCGCACTTTCGACAATGGGAGCATTCCCCACCAGATCCATCTTGGGGAGCTTCACGCCATCCTTCGGCGCCAAGAGGACTTCTACCCCTTTCTTAAGGACAACAGGGAGAAGATTGAGAAAATTCTCACTTTCCGCATCCCCTACTACGTGGGACCCCTCGCCAGAGGAAATAGCCGGTTTGCTTGGATGACCAGAAAGTCAGAAGAAACTATCACTCCCTGGAACTTCGAAGAGGTGGTGGACAAGGGAGCCAGCGCTCAGTCATTCATCGAACGGATGACTAACTTCGATAAGAACCTCCCCAATGAGAAGGTCCTGCCGAAACATTCCCTGCTCTACGAGTACTTTACCGTGTACAACGAGCTGACCAAGGTGAAATATGTCACCGAAGGGATGAGGAAGCCCGCATTCCTGTCAGGCGAACAAAAGAAGGCAATTGTGGACCTTCTGTTCAAGACCAATAGAAAGGTGACCGTGAAGCAGCTGAAGGAGGACTATTTCAAGAAAATTGAATGCTTCGACTCTGTGGAGATTAGCGGGGTCGAAGATCGGTTCAACGCAAGCCTGGGTACCTACCATGATCTGCTTAAGATCATCAAGGACAAGGATTTTCTGGACAATGAGGAGAACGAGGACATCCTTGAGGACATTGTCCTGACTCTCACTCTGTTCGAGGACCGGGAAATGATCGAGGAGAGGCTTAAGACCTACGCCCATCTGTTCGACGATAAAGTGATGAAGCAACTTAAACGGAGAAGATATACCGGATGGGGACGCCTTAGCCGCAAACTCATCAACGGAATCCGGGACAAACAGAGCGGAAAGACCATTCTTGATTTCCTTAAGAGCGACGGATTCGCTAATCGCAACTTCATGCAACTTATCCATGATGATTCCCTGACCTTTAAGGAGGACATCCAGAAGGCCCAAGTGTCTGGACAAGGTGACTCACTGCACGAGCATATCGCAAATCTGGCTGGTTCACCCGCTATTAAGAAGGGTATTCTCCAGACCGTGAAAGTCGTGGACGAGCTGGTCAAGGTGATGGGTCGCCATAAACCAGAGAACATTGTCATCGAGATGGCCAGGGAAAACCAGACTACCCAGAAGGGACAGAAGAACAGCAGGGAGCGGATGAAAAGAATTGAGGAAGGGATTAAGGAGCTCGGGTCACAGATCCTTAAAGAGCACCCGGTGGAAAACACCCAGCTTCAGAATGAGAAGCTCTATCTGTACTACCTTCAAAATGGACGCGATATGTATGTGGACCAAGAGCTTGATATCAACAGGCTCTCAGACTACGACGTGGACCACATCGTCCCTCAGAGCTTCCTCAAAGACGACTCAATTGACAATAAGGTGCTGACTCGCTCAGACAAGAACCGGGGAAAGTCAGATAACGTGCCCTCAGAGGAAGTCGTGAAAAAGATGAAGAACTATTGGCGCCAGCTTCTGAACGCAAAGCTGATCACTCAGCGGAAGTTCGACAATCTCACTAAGGCTGAGAGGGGCGGACTGAGCGAACTGGACAAAGCAGGATTCATTAAACGGCAACTTGTGGAGACTCGGCAGATTACTAAACATGTCGCCCAAATCCTTGACTCACGCATGAATACCAAGTACGACGAAAACGACAAACTTATCCGCGAGGTGAAGGTGATTACCCTGAAGTCCAAGCTGGTCAGCGATTTCAGAAAGGACTTTCAATTCTACAAAGTGCGGGAGATCAATAACTATCATCATGCTCATGACGCATATCTGAATGCCGTGGTGGGAACCGCCCTGATCAAGAAGTACCCAAAGCTGGAAAGCGAGTTCGTGTACGGAGACTACAAGGTCTACGACGTGCGCAAGATGATTGCCAAATCTGAGCAGGAGATCGGAAAGGCCACCGCAAAGTACTTCTTCTACAGCAACATCATGAATTTCTTCAAGACCGAAATCACCCTTGCAAACGGTGAGATCCGGAAGAGGCCGCTCATCGAGACTAATGGGGAGACTGGCGAAATCGTGTGGGACAAGGGCAGAGATTTCGCTACCGTGCGCAAAGTGCTTTCTATGCCTCAAGTGAACATCGTGAAGAAAACCGAGGTGCAAACCGGAGGCTTTTCTAAGGAATCAATCCTCCCCAAGCGCAACTCCGACAAGCTCATTGCAAGGAAGAAGGATTGGGACCCTAAGAAGTACGGCGGATTCGATTCACCAACTGTGGCTTATTCTGTCCTGGTCGTGGCTAAGGTGGAAAAAGGAAAGTCTAAGAAGCTCAAGAGCGTGAAGGAACTGCTGGGTATCACCATTATGGAGCGCAGCTCCTTCGAGAAGAACCCAATTGACTTTCTCGAAGCCAAAGGTTACAAGGAAGTCAAGAAGGACCTTATCATCAAGCTCCCAAAGTATAGCCTGTTCGAACTGGAGAATGGGCGGAAGCGGATGCTCGCCTCCGCTGGCGAACTTCAGAAGGGTAATGAGCTGGCTCTCCCCTCCAAGTACGTGAATTTCCTCTACCTTGCAAGCCATTACGAGAAGCTGAAGGGGAGCCCCGAGGACAACGAGCAAAAGCAACTGTTTGTGGAGCAGCATAAGCATTATCTGGACGAGATCATTGAGCAGATTTCCGAGTTTTCTAAACGCGTCATTCTCGCTGATGCCAACCTCGATAAAGTCCTTAGCGCATACAATAAGCACAGAGACAAACCAATTCGGGAGCAGGCTGAGAATATCATCCACCTGTTCACCCTCACCAATCTTGGTGCCCCTGCCGCATTCAAGTACTTCGACACCACCATCGACCGGAAACGCTATACCTCCACCAAAGAAGTGCTGGACGCCACCCTCATCCACCAGAGCATCACCGGACTTTACGAAACTCGGATTGACCTCTCACAGCTCGGAGGGGATGAGGGAGCTCCCAAGAAAAAGCGCAAGGTAGGTTAATGAgaattc

*Tbx6-related.b>CD4::mCherry*

Tbx6-related.b driver (Christiaen et al. 2009)

CD4::mCherry, human (Gline et al. 2015)

ggcgcgcccaacggagtacgcgtgtcaagtttaatggcgtaattaccgaacaactgttgataagtaatgaggaccccgctgcggtaacctttcgaaattgctggttgcaagcggtgttggtgcgataataaggaaaagcggtaagcgttattttttgatccaccacaaagaccaaacctatattgatacactaaactaaatcaaattcgaacctatccattaaagtgcgaaatatacatagaatgaacaataatccgagctatattatgggcaatattttggctagttttctgcttacaataatacataagaggcctacactaaatacggcggtatataaaactatgcaagacatacccatatattagttttaatcaaattgatttaaaatatttttaaaactattaggcaactttgagtagaataagggtttaacctaaacgtatgtggttttagccatcgatggaaacgttttacaattatcgttacttttttgaagaccttttcgttctatttttaagaagaacattcaaagaaatggaaaaccgttttcttacgaatcctatataccgttgttaattgtttaaaaacacgataaggatattatgttctgaatgtgtcccatcttaccccacagcactatataataaaattttagtttttgtgacagacttatgtcgcattcttaaacggatgatccccaactggttaacaacgtaacaagcttgcaaaagcgaaggtgacattggtaacaacgtacgataactattgtctactaataacaatagacttaacacataacatatttaggaaatggcttcatatggcggactgtcaacttagttgtcaaaattacctttaaatctctataaacgaagctgtttaataaaaaaaactaacaaggctattctacatcaaaccataataatgaaattatcaaacacatttttaaagcgatttcatcaaaccaacgcgccacatgcaagacggtatgcgtcacactgagttttggagtgttctgcatgcgctagacttgaatcagcaggagagttcggaggcttatcaggaagcagttgtccttgttaatgactcgttaagatcaaagtggcatcgaaaacgagtctcgctataaaacgggtttagtttcacagtacctcattccgctttctgttctcattggatatacaccaaactgaaagtacgagttaaatcgaaagagagaggaaattgtaagttttattggaccagacaagactatggcgaatatggcggccgcaaccATGAACCGGGGAGTCCCTTTTAGGCACTTGCTTCTGGTGCTGCAACTGGCGCTCCTCCCAGCAGCCACTCAGGGAAAGAAAGTGGTGCTGGGCAAAAAAGGGGATACAGTGGAACTGACCTGTACAGCTTCCCAGAAGAAGAGCATACAATTCCACTGGAAAAACTCCAACCAGATAAAGATTCTGGGAAATCAGGGCTCCTTCTTAACTAAAGGTCCATCCAAGCTGAATGATCGCGCTGACTCAAGAAGAAGCCTTTGGGACCAAGGAAACTTCCCCCTGATCATCAAGAATCTTAAGATAGAAGACTCAGATACTTACATCTGTGAAGTGGAGGACCAGAAGGAGGAGGTGCAATTGCTAGTGTTCGGATTGACTGCCAACTCTGACACCCACCTGCTTCAGGGGCAGAGCCTGACCCTGACCTTGGAGAGCCCCCCTGGTAGTAGCCCCTCAGTGCAATGTAGGAGTCCAAGGGGTAAAAACATACAGGGGGGGAAGACCCTCTCCGTGTCTCAGCTGGAGCTCCAGGATAGTGGCACCTGGACATGCACTGTCTTGCAGAACCAGAAGAAGGTGGAGTTCAAAATAGACATCGTGGTGCTAGCTTTCCAGAAGGCCTCCAGCATAGTCTATAAGAAAGAGGGGGAACAGGTGGAGTTCTCCTTCCCACTCGCCTTTACAGTTGAAAAGCTGACGGGCAGTGGCGAGCTGTGGTGGCAGGCGGAGAGGGCTTCCTCCTCCAAGTCTTGGATCACCTTTGACCTGAAGAACAAGGAAGTGTCTGTAAAACGGGTTACCCAGGACCCTAAGCTCCAGATGGGCAAGAAGCTCCCGCTCCACCTCACCCTGCCCCAGGCCTTGCCTCAGTATGCTGGCTCTGGAAACCTCACCCTGGCCCTTGAAGCGAAAACAGGAAAGTTGCATCAGGAAGTGAACCTGGTGGTGATGAGAGCCACTCAGCTCCAGAAAAATTTGACCTGTGAGGTGTGGGGACCCACCTCCCCTAAGCTGATGCTGAGCTTGAAACTGGAGAACAAGGAGGCAAAGGTCTCGAAGCGGGAGAAGGCGGTGTGGGTGCTGAACCCTGAGGCGGGGATGTGGCAGTGTCTGCTGAGTGACTCGGGACAGGTCCTGCTGGAATCCAACATCAAGGTTCTGCCCACATGGTCCACCCCGGTGCAGCCAATGGCCCTGATTGTGCTGGGGGGCGTCGCCGGCCTCCTGCTTTTCATTGGGCTAGGCATCTTCTTCTGTGTCAGGACTAGTGTGAGCAAGGGCGAGGAGGATAACATGGCCATCATCAAGGAGTTCATGCGCTTCAAGGTGCACATGGAGGGCTCCGTGAACGGCCACGAGTTCGAGATCGAGGGCGAGGGCGAGGGCCGCCCCTACGAGGGCACCCAGACCGCCAAGCTGAAGGTGACCAAGGGTGGCCCCCTGCCCTTCGCCTGGGACATCCTGTCCCCTCAGTTCATGTACGGCTCCAAGGCCTACGTGAAGCACCCCGCCGACATCCCCGACTACTTGAAGCTGTCCTTCCCCGAGGGCTTCAAGTGGGAGCGCGTGATGAACTTCGAGGACGGCGGCGTGGTGACCGTGACCCAGGACTCCTCCCTGCAGGACGGCGAGTTCATCTACAAGGTGAAGCTGCGCGGCACCAACTTCCCCTCCGACGGCCCCGTAATGCAGAAGAAGACCATGGGCTGGGAGGCCTCCTCCGAGCGGATGTACCCCGAGGACGGCGCCCTGAAGGGCGAGATCAAGCAGAGGCTGAAGCTGAAGGACGGCGGCCACTACGACGCTGAGGTCAAGACCACCTACAAGGCCAAGAAGCCCGTGCAGCTGCCCGGCGCCTACAACGTCAACATCAAGTTGGACATCACCTCCCACAACGAGGACTACACCATCGTGGAACAGTACGAACGCGCCGAGGGCCGCCACTCCACCGGCGGCATGGACGAGCTGTACAAGTAAgaattc

*Islet -7216/-3950 + bpFOG>tagRFP*

Islet -7216/-3950 (Stolfi et al. 2010)

bpFOG (Rothbächer et al. 2007)

TagRFP (Merzlyak et al. 2007)

ggcgcgccaagtataccacgcgagttagttcggccaggaagcgcgctgtttattatcaagttattatgacgtcatataaaatacgtcacatttgtagcacctggttatctgccttcccacggttaaaacagatgcgacctcggttatcagtggtttgacatggagatcgtaaatcaagcgacgaatatctgtgacacgtcacgtgacgagattgctgacataccaggacacttaacggtatctggttaggggaagtacctgtagcgtcatacacataacctgcataatgctttatagcttcgaatacgatagcgaagatcaaattaattaaaacaacgaagagaaggattcgaagcaaaacgacagtcgttaaaacacactacgggtaaaaattacttggggaaaagcgtggctgttacatccaaatcaaaatgtaagccgtcaatggtttgtgaacaaacggtaactcttgtcacctctaagctaaactcattttttgcagagtttaaaaataccgagtagcaactgccgtctggaacgaataaatgaatctaacgacgcagacgttatgacaggtgtgttcatacaccatgtttcccttttacgggaaagcgtataaaatatatatttacattcttgcctaaagtataaacgaacttttattgaatggtcaaaatgggtcatcttaaattccattataacttttccatttcaaacatttttccgcagcatcgtcataaaacagggattccaatagaaacatcataaaacaccaaaccatgttgtttatggatcaccgtgtttgttgtattaacaagagttataactacaacatgacgtgactagattgacacaacaactatggttggcagtcaaaacacacgaccccaccccataagatggagccacgccaccaaatcaacacagcagaagctccaggtggtttggtaggttctgttatcacaacactacaggccatgagatctatgatctataccgtctgttatatttagtggatttgtgattttaaccgaacaccttcactagtttaaaccaaccgacaactcaccgcgaacaatcagcgaatatttcattaaaccgacaaaaatttccagttaatttaaaatatagtaagttggggtaagatgggacatgtttttattctcatctcgtcccatttggtagtcaacaaagaatatttacagaattataaattgaaatatcttcacgaagagaaaaatattgttatttgttaaaaacacgatcaggaaatatgggatattttgtgctaaccgtattcatatttgcaacccactgtgagttgcttcttataaggtttaacagccacggttttaactaaattttatttattttaaccttagcgtgttataacgactgttatttttctgttaattcccatttcttcgccatgttagttaatatgacttttgttattgtaattatataaaaatatattttttaaaataagtctaaaagtaaatgtctaagttgtatttacaaatttccgcaccagaataggcagcaggcaaccgcggatcgaaacaagtccctgatttatggttttgttgcaactaattatggctgtacagcacacaggtcccaggattgccgtaaaacttaatttgctggaatatgttgaacaacagttgttacattctgttgttttattaagttcagcggccacgcttttttaagccttttattccagtattttcgctcgtagcgtgttgataacgactgttgttttgctgctaattctcttcgttgttttaattaaacttcgctatcgtattcaaaatatgttcatttagttggtaggttatgtgtgtaagtgacatcataacaacttgtatgaggcaaacaaggggttatgacgtaataatagaattattgatgtttcacagggtgaggtgtgacgtaataatagttctgacagttaaagtgattttgaataaaaacaaaaaacagaatgtttaagtcgcgaaaatcgcgtgggcagcgttaaccagagcatcgcatgtgacaagcgaacgaaatcccagcagatctacgacgttggtccgctgagagttcgagcgaattcgggatgtaacgtcgatgaaagacgaaaagcggtccttgcctcgacaagaaaggacgaaccgcgcgatagtagattttatggcgtatggtgttctataacacagctgtgggttgaagtggtttcgaatatttaagcaaggtacgacttgaccggttacgtcactttgaccacgtggtcagctctcatcaatatttattttaaatttgattaaacagcgacctcaggtgagcggcctaattatgcgcctctatgacgtcatagtttaaacttcttttaatagcggaatcgaagataaataggttgcgttaccctgggggtcgagatccaaatttcgttacttcgattgtttaattgattcgacatcgagttaaagcgcgagatttgattacatttccattgttcaatccataattcgctttgtgacgtaacacaaacttaacaaacacgtggtagcgcgcaagagctaattttttaaacgtttcttgattttttttcgattttttttattaaaaaataaaataaattaatattttgttgaaatacgtcgaaaccgcgaaaacgcgatcgacgcaattcaatttgtaaatgaaatatcgaccagtggcgccgatagcggataatcctcacaattcttgtgataaattagtttcttactcggataatctgtgtgccggtttgattgtcgcacccaaggtgattggggggggggggatattacgtcattatgacaaataatttcgattaaaattggtaaaaatataaattaggggagtttttagttttgttttaaaagattttatttaaaaattaaaaaaaaaatcgaaaatttttgaatttgatcgattagctgtaattacgtcatcaatccgctcattatgacgtcatacgtcgccatattgcgtcataatcgggcgtcagaatgtcgtcacaaaccatattgtgttgaattaattggaattatgaaacgccgctcggtcgggggatttgtgacgtcacaaacgtgaatgttacgacacaaacggcgaactcgggcgaacttttagctttgcggataataagggtaagtgacgcaacaatgacgcaacaatgttgtcccttgtgacgtaacagagtgtattgtaaccgttgaattgagtctcgagcagctgaagcttgcatgcctgcaggtcgactctagaggatccggcaaAGCTTCGTGTATTGTACCGGCCCATTGTCAATCATGCAAACTTGATATTATATTGACAAGAGAAGAAGGCAGTTTAAATTAAAACTCTAAAGTAGAGAGACATTAATCTCAGCTGACAAGGCAGGTGGTCACAGTAAGTTCATTTAAATAGTTGGCCAACAATAGCCTTTCCAAGAAAGTATTTTTGTTCCAGGTCTATACAAAAATAACACACATAgcggccgcaaccATGGTGTCTAAGGGCGAAGAGCTGATTAAGGAGAACATGCACATGAAGCTGTACATGGAGGGCACCGTGAACAACCACCACTTCAAGTGCACATCCGAGGGCGAAGGCAAGCCCTACGAGGGCACCCAGACCATGAGAATCAAGGTGGTCGAGGGCGGCCCTCTCCCCTTCGCCTTCGACATCCTGGCTACCAGCTTCATGTACGGCAGCAGAACCTTCATCAACCACACCCAGGGCATCCCCGACTTCTTTAAGCAGTCCTTCCCTGAGGGCTTCACATGGGAGAGAGTCACCACATACGAAGACGGGGGCGTGCTGACCGCTACCCAGGACACCAGCCTCCAGGACGGCTGCCTCATCTACAACGTCAAGATCAGAGGGGTGAACTTCCCATCCAACGGCCCTGTGATGCAGAAGAAAACACTCGGCTGGGAGGCCAACACCGAGATGCTGTACCCCGCTGACGGCGGCCTGGAAGGCAGAAGCGACATGGCCCTGAAGCTCGTGGGCGGGGGCCACCTGATCTGCAACTTCAAGACCACATACAGATCCAAGAAACCCGCTAAGAACCTCAAGATGCCCGGCGTCTACTATGTGGACCACAGACTGGAAAGAATCAAGGAGGCCGACAAAGAGACCTACGTCGAGCAGCACGAGGTGGCTGTGGCCAGATACTGCGACCTCCCTAGCAAACTGGGGCACAACTTAATTAAgaattc

*Eef1a>H2B::CFP*

Eef1a -1955/-1 (Sasakura et al. 2010)

H2B::CFP (Stolfi et al. 2010)

ggcgcgccgtgacgggaaaacgatagtcgttataacacgagtattcgtacacctcgtgcgagctaacgagctaccatatatgttgtgggcgaataaaggttttataaatataacattggttttataaataaaacaacgccattttaaagtcggttacataattctgtaactagttcaaattgaacggtaaacgtaaataaaaaccttgaccgtcttacccaattatataaaaacactttgaacgctttttaagatggaagggtatggccatgcctagataattctgtggaccatctcaccccaacctattacagaacggtcgtaataatgaaaatgggtaccatttttaggcatatagactgattcctcctttctagaaacgtaagcagtatacacagaaaaaatgaagtgtgattctgtgcaattaaaccgttctaaattcatagccgactgaatttctaattaagtgaatgtctgacctagatttattgttaagtttagcaccaaatctgagccagcgataagcagtctaattaaattggctgctggcgataaaataggtcatcctgaaaaatcgtttgcgcctttatttaaaatatagtagagtggggaaagacgggacatcttatcgttctattttctcgtcccatttcgtagtaaacaaagaacattcaaaaaatataaaaccataacttcaaaacttcaatagaccgttgtcaactgtttaaaacacaataagagaatttggatattatgtgctaaaggtgtcccatctccccccaccctactatatctgtttatagttctgtggggtaagatgagataccgttaacacctaaacatttttactttaaacaatcaaccacgttttttatagtcgtaatggacatgtggttacataattctgaaaatattttttgcccccgaccaaaagacgcgaagagtaaaaacatgtctcagcttatattccccacataaatatatttttgtactgtttggtgaatttataaacttatattaccatgcatatacgttatgttactggtattttctcagtaggcaaattcatttgtccacgttttataggttttcaatatttatgatttttaaaatgctaaaaatgtgggaggggggttgaaagtacaatacaaacacacaaaacaactcaaactaaagatttatagttatgctaattcacctacacaatataacaagatgtgtaatgcaaccatgtgtttatgatgagcgctaacatattttgtaaccactcaaattccccgccacacgaggataatgaataggtgactctgtagtctgtacatcttagactgaaataaagattataaatctacgaaataaaataatttctgctcactgattatacttctgttttatagattagaaaccgtttctaataaatgacctaattcgctatacacacacgctgtgcgcgagataatcattctcgcaccccgtttattgtgttaaaattgccgcctagattcacaaagcgtgacggctagagccagcaacgtgtcgccttcaattacgcaacatccgggttgcgcaattctggatataaaagaactaacaaagatgacgtagctacctttttcagttcagacttacgaaagactcacgtgtcggcggtctacttgtccttttcgagctgtggcaatttggtgagtggttctatcttatatctgagtacatctctaaggaattatagtttgattagttaagtttttattgttaggaaagatgaaatcattaggttttacttagtttaagtatgttagtactggttaggcgtttgaattattgaaaaactcagttcgttaactgtagtagttctggtagcttagcaagtataccctgtatacgccttttggctttttaacaataacttaaacttattttacagcaaatttctgtgcattcggttaaccccaaccttccaaagcggccgcAACCATGGTTGCATCCAAAGGTCCCAAGAAAGCTATGAAGAGCACACCCCCAGTAGAGAAGAAGGAACGCAAGAATAAGCGCCATCGACGACGAAAGGAAAGCTTCGGCATCTACATTTACAAAGTGCTGAAGCAAGTCCACCCTGACACTGGTATCTCCAGCAAGGCAATGAACATCATGAATTCATTTGTTCACGACCTATTCGAAAGAGTTGCCGGAGAATCTTCTCGTCTCTGTAGCTACAACAAGCGCTCCACTATCAGCAGTCGGGAAGTGCAAACCGCAGTTCGCCTTTTGCTTCCAGGGGAGCTTGCAAAACATGCTGTATCGGAGGGTACAAAGGCCGTTACAAAGTACACCAGCTCAAAAACTAGTTCCAAAGGTGAAGAACTTTTCACTGGAGTGGTGCCTATCTTGGTTGAGCTTGACGGTGATGTGAACGGTCACAAATTCTCTGTAAGTGGTGAAGGAGAGGGAGACGCTACCTACGGCAAGTTAACGCTGAAATTTATATGCACTACGGGAAAGCTGCCTGTACCGTGGCCTACACTGGTTACCACTCTGACTTGGGGAGTACAATGCTTCGCCCGCTATCCGGACCACATGAAACGCCATGACTTCTTTAAATCAGCTATGCCAGAAGGATACGTGCAAGAACGAACCATTTTCTTCAAAGATGATGGTAATTATAAAACAAGGGCGGAAGTTAAATTCGAAGGAGACACGCTCGTAAACAGAATCGAACTTAAGGGTATCGACTTCAAGGAGGATGGCAACATTCTTGGACACAAACTGGAGTACAACGCCATTTCCGATAATGTTTACATTACTGCTGATAAACAGAAGAACGGAATTAAGGCGAATTTTAAAATCAGACATAACATTGAAGATGGTGGAGTGCAATTGGCTGATCACTACCAGCAAAATACTCCTATCGGAGACGGCCCTGTGTTGCTTCCTGACAACCACTACTTAAGTACGCAATCAGCTTTATCCAAAGATCCCAATGAAAAGCGAGATCACATGGTGCTGCTCGAATTTGTTACTGCTGCTGGTATTACACACGGAATGGACGAGCTGTACAAGTAAgaattc

**New constructs:**

*Eef1a>Nova(MLN)*

Eef1a -1955/-1 (Sasakura et al. 2010)

Nova(MLN)

ggcgcgccgtgacgggaaaacgatagtcgttataacacgagtattcgtacacctcgtgcgagctaacgagctaccatatatgttgtgggcgaataaaggttttataaatataacattggttttataaataaaacaacgccattttaaagtcggttacataattctgtaactagttcaaattgaacggtaaacgtaaataaaaaccttgaccgtcttacccaattatataaaaacactttgaacgctttttaagatggaagggtatggccatgcctagataattctgtggaccatctcaccccaacctattacagaacggtcgtaataatgaaaatgggtaccatttttaggcatatagactgattcctcctttctagaaacgtaagcagtatacacagaaaaaatgaagtgtgattctgtgcaattaaaccgttctaaattcatagccgactgaatttctaattaagtgaatgtctgacctagatttattgttaagtttagcaccaaatctgagccagcgataagcagtctaattaaattggctgctggcgataaaataggtcatcctgaaaaatcgtttgcgcctttatttaaaatatagtagagtggggaaagacgggacatcttatcgttctattttctcgtcccatttcgtagtaaacaaagaacattcaaaaaatataaaaccataacttcaaaacttcaatagaccgttgtcaactgtttaaaacacaataagagaatttggatattatgtgctaaaggtgtcccatctccccccaccctactatatctgtttatagttctgtggggtaagatgagataccgttaacacctaaacatttttactttaaacaatcaaccacgttttttatagtcgtaatggacatgtggttacataattctgaaaatattttttgcccccgaccaaaagacgcgaagagtaaaaacatgtctcagcttatattccccacataaatatatttttgtactgtttggtgaatttataaacttatattaccatgcatatacgttatgttactggtattttctcagtaggcaaattcatttgtccacgttttataggttttcaatatttatgatttttaaaatgctaaaaatgtgggaggggggttgaaagtacaatacaaacacacaaaacaactcaaactaaagatttatagttatgctaattcacctacacaatataacaagatgtgtaatgcaaccatgtgtttatgatgagcgctaacatattttgtaaccactcaaattccccgccacacgaggataatgaataggtgactctgtagtctgtacatcttagactgaaataaagattataaatctacgaaataaaataatttctgctcactgattatacttctgttttatagattagaaaccgtttctaataaatgacctaattcgctatacacacacgctgtgcgcgagataatcattctcgcaccccgtttattgtgttaaaattgccgcctagattcacaaagcgtgacggctagagccagcaacgtgtcgccttcaattacgcaacatccgggttgcgcaattctggatataaaagaactaacaaagatgacgtagctacctttttcagttcagacttacgaaagactcacgtgtcggcggtctacttgtccttttcgagctgtggcaatttggtgagtggttctatcttatatctgagtacatctctaaggaattatagtttgattagttaagtttttattgttaggaaagatgaaatcattaggttttacttagtttaagtatgttagtactggttaggcgtttgaattattgaaaaactcagttcgttaactgtagtagttctggtagcttagcaagtataccctgtatacgccttttggctttttaacaataacttaaacttattttacagcaaatttctgtgcattcggttaaccccaaccttccaaagcggccgcAACCATGCTAAATGCAATGGAGTATGAATGCCAGTACAATGCTGGCTACAGCATTGTGTCTAACGGTAACGAATACGGTCTCATACAGGCCTACACGGCACACGATTACCCCCTTGAAAACGGAGTGACGTTTTCAGCACCTCCGCCGGGCCAGCTCATTCTTAAAGTTCTAATACCGGGGTACGCTGCGGGGGCGGTGATCGGGAAAGGCGGTCAGATTATTGTACAACTTCAGAAAGATTCAGGGGCCATTATTAAGCTGTCAAAAGCGAAGGACTTTTACCCCGGAACCCAAGACCGAGTCGTTTTGATCCAAGGAACCGCCGAAGGCTTGATGAAGGTGCAAAATACCATTATAGAGAAGGTGTACGAGTTCCCTGTGCCCAAAGATTTAGCTGCGATCATCGGAGACCGACCGAAACAGGTGAAAATCATCGTACCCAACACAACTGCGGGACTGGTAATAGGAAAGGCCGGCGCAACGATAAAGACCATTATGGAAGAGAGTGGATCGAAGGTTCAACTCTCGCAAAAGCCAGACGGGGTAAACGTCCAAGAACGAGTCATCACAATCAAAGGAGAGAAGCACCAACTCATGACAGCATCTAATATTATTATTGATAAAATTAAAGACGACCCTCAAAGCGCCAGTTGCCCTCACATAAGTTACTCTGGCATCGCTGGCCCGATCGCTAACGCGAATCCCACCGGATCGCCCTACGCTGCTGGCTCGGCTGCATTAGTTGACGCTTCGCACCCATCCGTGGCCGCTATGTTGGGACATTATGTTATCCCAGGCCAACAGGTGCTGCAGACAGCAATGCCACTCTCCCATCACCCGCACCAGTCCGCGTTGTCCAGCGGCTCAGTGACACCGGCGCCTGAACTGACGACCATAAATCACGCCATGACAACGTTAGCGAACTATGGCTACACCTTAGGAGGCGTGAACTATGGTACCTTGGGTGTAATGCCTAGTGTACATCCAAGTGTACACCCTGGCATCGCTACCTCGGTCGGGATGATCTCTGCAGGCTCCCTAGCAGGAAGTCCAATCCCTTCAGCTACCCCCTTGCTCTCTGCCACTGCTCTACCGACGGAATCCAGTATTCCGACGGCTGTTCCCACTGCCCAAGCCATTTCAATGCAGAGCAATTACCTTGCAAACTTGGCTAATGCTGGTTACCTGACTACCGGTCACCCACAGTTGCTTGGAGCGACTATCCTAAGCATCGAAAAGTCAAGCGACGGACAAAAAGAAACAATTGAACTGGCAATTCCCGAAAACCTGATCGGAGCAGTCCTCGGAAAAGCGGGAAGGACACTGGTTGAGTATCAGGATGTATCAGGGGCGAAAATTCAAATTTCTAAAAAGGGTGATTACGTCGCCGGGACCAGGAACAGGAGGGTTACGATTACGGGGAAGCCCCCATGCCCACAGACTGCGCAGTTTCTTATTACGCAACGTGTCGCCTCTGCGCAAAACGCAAGGGCACAGCAGGCTAAGTTACTGTAGgaattc

*Eef1a>Nova(MMM)*

Eef1a -1955/-1 (Sasakura et al. 2010)

Nova(MMM)

ggcgcgccgtgacgggaaaacgatagtcgttataacacgagtattcgtacacctcgtgcgagctaacgagctaccatatatgttgtgggcgaataaaggttttataaatataacattggttttataaataaaacaacgccattttaaagtcggttacataattctgtaactagttcaaattgaacggtaaacgtaaataaaaaccttgaccgtcttacccaattatataaaaacactttgaacgctttttaagatggaagggtatggccatgcctagataattctgtggaccatctcaccccaacctattacagaacggtcgtaataatgaaaatgggtaccatttttaggcatatagactgattcctcctttctagaaacgtaagcagtatacacagaaaaaatgaagtgtgattctgtgcaattaaaccgttctaaattcatagccgactgaatttctaattaagtgaatgtctgacctagatttattgttaagtttagcaccaaatctgagccagcgataagcagtctaattaaattggctgctggcgataaaataggtcatcctgaaaaatcgtttgcgcctttatttaaaatatagtagagtggggaaagacgggacatcttatcgttctattttctcgtcccatttcgtagtaaacaaagaacattcaaaaaatataaaaccataacttcaaaacttcaatagaccgttgtcaactgtttaaaacacaataagagaatttggatattatgtgctaaaggtgtcccatctccccccaccctactatatctgtttatagttctgtggggtaagatgagataccgttaacacctaaacatttttactttaaacaatcaaccacgttttttatagtcgtaatggacatgtggttacataattctgaaaatattttttgcccccgaccaaaagacgcgaagagtaaaaacatgtctcagcttatattccccacataaatatatttttgtactgtttggtgaatttataaacttatattaccatgcatatacgttatgttactggtattttctcagtaggcaaattcatttgtccacgttttataggttttcaatatttatgatttttaaaatgctaaaaatgtgggaggggggttgaaagtacaatacaaacacacaaaacaactcaaactaaagatttatagttatgctaattcacctacacaatataacaagatgtgtaatgcaaccatgtgtttatgatgagcgctaacatattttgtaaccactcaaattccccgccacacgaggataatgaataggtgactctgtagtctgtacatcttagactgaaataaagattataaatctacgaaataaaataatttctgctcactgattatacttctgttttatagattagaaaccgtttctaataaatgacctaattcgctatacacacacgctgtgcgcgagataatcattctcgcaccccgtttattgtgttaaaattgccgcctagattcacaaagcgtgacggctagagccagcaacgtgtcgccttcaattacgcaacatccgggttgcgcaattctggatataaaagaactaacaaagatgacgtagctacctttttcagttcagacttacgaaagactcacgtgtcggcggtctacttgtccttttcgagctgtggcaatttggtgagtggttctatcttatatctgagtacatctctaaggaattatagtttgattagttaagtttttattgttaggaaagatgaaatcattaggttttacttagtttaagtatgttagtactggttaggcgtttgaattattgaaaaactcagttcgttaactgtagtagttctggtagcttagcaagtataccctgtatacgccttttggctttttaacaataacttaaacttattttacagcaaatttctgtgcattcggttaaccccaaccttccaaagcggccgcAACCatgatgatgacggccgtagtacctatgccgaacgggacatatcttatcgagtcgcgaaaaagaccgctggaagaaccgattgaactggtcgatttcaagagagaacgctccgaaatggaagATTACCCCCTTGAAAACGGAGTGACGTTTTCAGCACCTCCGCCGGGCCAGCTCATTCTTAAAGTTCTAATACCGGGGTACGCTGCGGGGGCGGTGATCGGGAAAGGCGGTCAGATTATTGTACAACTTCAGAAAGATTCAGGGGCCATTATTAAGCTGTCAAAAGCGAAGGACTTTTACCCCGGAACCCAAGACCGAGTCGTTTTGATCCAAGGAACCGCCGAAGGCTTGATGAAGGTGCAAAATACCATTATAGAGAAGGTGTACGAGTTCCCTGTGCCCAAAGATTTAGCTGCGATCATCGGAGACCGACCGAAACAGGTGAAAATCATCGTACCCAACACAACTGCGGGACTGGTAATAGGAAAGGCCGGCGCAACGATAAAGACCATTATGGAAGAGAGTGGATCGAAGGTTCAACTCTCGCAAAAGCCAGACGGGGTAAACGTCCAAGAACGAGTCATCACAATCAAAGGAGAGAAGCACCAACTCATGACAGCATCTAATATTATTATTGATAAAATTAAAGACGACCCTCAAAGCGCCAGTTGCCCTCACATAAGTTACTCTGGCATCGCTGGCCCGATCGCTAACGCGAATCCCACCGGATCGCCCTACGCTGCTGGCTCGGCTGCATTAGTTGACGCTTCGCACCCATCCGTGGCCGCTATGTTGGGACATTATGTTATCCCAGGCCAACAGGTGCTGCAGACAGCAATGCCACTCTCCCATCACCCGCACCAGTCCGCGTTGTCCAGCGGCTCAGTGACACCGGCGCCTGAACTGACGACCATAAATCACGCCATGACAACGTTAGCGAACTATGGCTACACCTTAGGAGGCGTGAACTATGGTACCTTGGGTGTAATGCCTAGTGTACATCCAAGTGTACACCCTGGCATCGCTACCTCGGTCGGGATGATCTCTGCAGGCTCCCTAGCAGGAAGTCCAATCCCTTCAGCTACCCCCTTGCTCTCTGCCACTGCTCTACCGACGGAATCCAGTATTCCGACGGCTGTTCCCACTGCCCAAGCCATTTCAATGCAGAGCAATTACCTTGCAAACTTGGCTAATGCTGGTTACCTGACTACCGGTCACCCACAGTTGCTTGGAGCGACTATCCTAAGCATCGAAAAGTCAAGCGACGGACAAAAAGAAACAATTGAACTGGCAATTCCCGAAAACCTGATCGGAGCAGTCCTCGGAAAAGCGGGAAGGACACTGGTTGAGTATCAGGATGTATCAGGGGCGAAAATTCAAATTTCTAAAAAGGGTGATTACGTCGCCGGGACCAGGAACAGGAGGGTTACGATTACGGGGAAGCCCCCATGCCCACAGACTGCGCAGTTTCTTATTACGCAACGTGTCGCCTCTGCGCAAAACGCAAGGGCACAGCAGGCTAAGTTACTGTAGgaattc

*Islet -7216/-3950 + bpFOG>Nova(MLN) rescue*

Islet -7216/-3950 (Stolfi et al. 2010)

bpFOG (Rothbächer et al. 2007)

Nova(MLN) rescue

Silent mutation in Nova2.1 target sequence

ggcgcgccaagtataccacgcgagttagttcggccaggaagcgcgctgtttattatcaagttattatgacgtcatataaaatacgtcacatttgtagcacctggttatctgccttcccacggttaaaacagatgcgacctcggttatcagtggtttgacatggagatcgtaaatcaagcgacgaatatctgtgacacgtcacgtgacgagattgctgacataccaggacacttaacggtatctggttaggggaagtacctgtagcgtcatacacataacctgcataatgctttatagcttcgaatacgatagcgaagatcaaattaattaaaacaacgaagagaaggattcgaagcaaaacgacagtcgttaaaacacactacgggtaaaaattacttggggaaaagcgtggctgttacatccaaatcaaaatgtaagccgtcaatggtttgtgaacaaacggtaactcttgtcacctctaagctaaactcattttttgcagagtttaaaaataccgagtagcaactgccgtctggaacgaataaatgaatctaacgacgcagacgttatgacaggtgtgttcatacaccatgtttcccttttacgggaaagcgtataaaatatatatttacattcttgcctaaagtataaacgaacttttattgaatggtcaaaatgggtcatcttaaattccattataacttttccatttcaaacatttttccgcagcatcgtcataaaacagggattccaatagaaacatcataaaacaccaaaccatgttgtttatggatcaccgtgtttgttgtattaacaagagttataactacaacatgacgtgactagattgacacaacaactatggttggcagtcaaaacacacgaccccaccccataagatggagccacgccaccaaatcaacacagcagaagctccaggtggtttggtaggttctgttatcacaacactacaggccatgagatctatgatctataccgtctgttatatttagtggatttgtgattttaaccgaacaccttcactagtttaaaccaaccgacaactcaccgcgaacaatcagcgaatatttcattaaaccgacaaaaatttccagttaatttaaaatatagtaagttggggtaagatgggacatgtttttattctcatctcgtcccatttggtagtcaacaaagaatatttacagaattataaattgaaatatcttcacgaagagaaaaatattgttatttgttaaaaacacgatcaggaaatatgggatattttgtgctaaccgtattcatatttgcaacccactgtgagttgcttcttataaggtttaacagccacggttttaactaaattttatttattttaaccttagcgtgttataacgactgttatttttctgttaattcccatttcttcgccatgttagttaatatgacttttgttattgtaattatataaaaatatattttttaaaataagtctaaaagtaaatgtctaagttgtatttacaaatttccgcaccagaataggcagcaggcaaccgcggatcgaaacaagtccctgatttatggttttgttgcaactaattatggctgtacagcacacaggtcccaggattgccgtaaaacttaatttgctggaatatgttgaacaacagttgttacattctgttgttttattaagttcagcggccacgcttttttaagccttttattccagtattttcgctcgtagcgtgttgataacgactgttgttttgctgctaattctcttcgttgttttaattaaacttcgctatcgtattcaaaatatgttcatttagttggtaggttatgtgtgtaagtgacatcataacaacttgtatgaggcaaacaaggggttatgacgtaataatagaattattgatgtttcacagggtgaggtgtgacgtaataatagttctgacagttaaagtgattttgaataaaaacaaaaaacagaatgtttaagtcgcgaaaatcgcgtgggcagcgttaaccagagcatcgcatgtgacaagcgaacgaaatcccagcagatctacgacgttggtccgctgagagttcgagcgaattcgggatgtaacgtcgatgaaagacgaaaagcggtccttgcctcgacaagaaaggacgaaccgcgcgatagtagattttatggcgtatggtgttctataacacagctgtgggttgaagtggtttcgaatatttaagcaaggtacgacttgaccggttacgtcactttgaccacgtggtcagctctcatcaatatttattttaaatttgattaaacagcgacctcaggtgagcggcctaattatgcgcctctatgacgtcatagtttaaacttcttttaatagcggaatcgaagataaataggttgcgttaccctgggggtcgagatccaaatttcgttacttcgattgtttaattgattcgacatcgagttaaagcgcgagatttgattacatttccattgttcaatccataattcgctttgtgacgtaacacaaacttaacaaacacgtggtagcgcgcaagagctaattttttaaacgtttcttgattttttttcgattttttttattaaaaaataaaataaattaatattttgttgaaatacgtcgaaaccgcgaaaacgcgatcgacgcaattcaatttgtaaatgaaatatcgaccagtggcgccgatagcggataatcctcacaattcttgtgataaattagtttcttactcggataatctgtgtgccggtttgattgtcgcacccaaggtgattggggggggggggatattacgtcattatgacaaataatttcgattaaaattggtaaaaatataaattaggggagtttttagttttgttttaaaagattttatttaaaaattaaaaaaaaaatcgaaaatttttgaatttgatcgattagctgtaattacgtcatcaatccgctcattatgacgtcatacgtcgccatattgcgtcataatcgggcgtcagaatgtcgtcacaaaccatattgtgttgaattaattggaattatgaaacgccgctcggtcgggggatttgtgacgtcacaaacgtgaatgttacgacacaaacggcgaactcgggcgaacttttagctttgcggataataagggtaagtgacgcaacaatgacgcaacaatgttgtcccttgtgacgtaacagagtgtattgtaaccgttgaattgagtctcgagcagctgaagcttgcatgcctgcaggtcgactctagaggatccggcaaAGCTTCGTGTATTGTACCGGCCCATTGTCAATCATGCAAACTTGATATTATATTGACAAGAGAAGAAGGCAGTTTAAATTAAAACTCTAAAGTAGAGAGACATTAATCTCAGCTGACAAGGCAGGTGGTCACAGTAAGTTCATTTAAATAGTTGGCCAACAATAGCCTTTCCAAGAAAGTATTTTTGTTCCAGGTCTATACAAAAATAACACACATAgcggccgcaaccATGCTAAATGCAATGGAGTATGAATGCCAGTACAATGCTGGCTACAGCATTGTGTCTAACGGTAACGAATACGGTCTCATACAGGCCTACACGGCACACGATTACCCCCTTGAAAACGGAGTGACGTTTTCAGCACCTCCGCCcGGCCAGCTCATTCTTAAAGTTCTAATACCGGGGTACGCTGCGGGGGCGGTGATCGGGAAAGGCGGTCAGATTATTGTACAACTTCAGAAAGATTCAGGGGCCATTATTAAGCTGTCAAAAGCGAAGGACTTTTACCCCGGAACCCAAGACCGAGTCGTTTTGATCCAAGGAACCGCCGAAGGCTTGATGAAGGTGCAAAATACCATTATAGAGAAGGTGTACGAGTTCCCTGTGCCCAAAGATTTAGCTGCGATCATCGGAGACCGACCGAAACAGGTGAAAATCATCGTACCCAACACAACTGCGGGACTGGTAATAGGAAAGGCCGGCGCAACGATAAAGACCATTATGGAAGAGAGTGGATCGAAGGTTCAACTCTCGCAAAAGCCAGACGGGGTAAACGTCCAAGAACGAGTCATCACAATCAAAGGAGAGAAGCACCAACTCATGACAGCATCTAATATTATTATTGATAAAATTAAAGACGACCCTCAAAGCGCCAGTTGCCCTCACATAAGTTACTCTGGCATCGCTGGCCCGATCGCTAACGCGAATCCCACCGGATCGCCCTACGCTGCTGGCTCGGCTGCATTAGTTGACGCTTCGCACCCATCCGTGGCCGCTATGTTGGGACATTATGTTATCCCAGGCCAACAGGTGCTGCAGACAGCAATGCCACTCTCCCATCACCCGCACCAGTCCGCGTTGTCCAGCGGCTCAGTGACACCGGCGCCTGAACTGACGACCATAAATCACGCCATGACAACGTTAGCGAACTATGGCTACACCTTAGGAGGCGTGAACTATGGTACCTTGGGTGTAATGCCTAGTGTACATCCAAGTGTACACCCTGGCATCGCTACCTCGGTCGGGATGATCTCTGCAGGCTCCCTAGCAGGAAGTCCAATCCCTTCAGCTACCCCCTTGCTCTCTGCCACTGCTCTACCGACGGAATCCAGTATTCCGACGGCTGTTCCCACTGCCCAAGCCATTTCAATGCAGAGCAATTACCTTGCAAACTTGGCTAATGCTGGTTACCTGACTACCGGTCACCCACAGTTGCTTGGAGCGACGTCAGGCCTCGGCGGTCTCACCACAGTGTCCCAGCACCCGCCACCAGCGGCGACACCAACGAGTTTTTCCGTCGCTTCTACCCCTTCTACCCCTGGTCTGCCGGTTTCATTTAGCCCCCATTCAACCGTGAGTATCCTAAGCATCGAAAAGTCAAGCGACGGACAAAAAGAAACAATTGAACTGGCAATTCCCGAAAACCTGATCGGAGCAGTCCTCGGAAAAGCGGGAAGGACACTGGTTGAGTATCAGGATGTATCAGGGGCGAAAATTCAAATTTCTAAAAAGGGTGATTACGTCGCCGGGACCAGGAACAGGAGGGTTACGATTACGGGGAAGCCCCCATGCCCACAGACTGCGCAGTTTCTTATTACGCAACGTGTCGCCTCTGCGCAAAACGCAAGGGCACAGCAGGCTAAGTTACTGTAGgaattc

*Vacht -2083/+15>Unc-76::mCherry*

Vacht (Slc18a3, Cirobu.g00000742) -2083/+15 (Based on Yoshida et al. 2004)

Unc-76::mCherry

GGCGCGCCattacgtcgtaaacctttggctaccatcatctgcctcaaaacaaaattaattaaagaaatgcgttagtgtatcctttgactcggaatcaatcaaatcaagcaaaaatcaatatgtgaaattaaccatttagaccttgtgtcattcccattgcggtgacttgtcctagttgtgcgtttttatcagcagattgatttaacagtcaccggaacaggcaagacaatatttcaaaccagccaatgttaatttcagacaaatgaagcaatctgaaaatcagaacaaatcaaaaaacataagttttggtttttaaagcatagaaaacgtaccgtattttttaatgtaattgttaaattttgttatttaatatagtagggtagggggagatgggacactttttcattctgttttctcgtcttggtagcaaacaaaaacattaaaagaattataaaaccgtatcctcgcgactcctacagaccgtttaaaacaggatatttggatattctgttctaaaggtgtcccatcttaccccacagtactatacactatacacattctgtaccgatatattttattgattaaatttgaagttgttaaacttaattacgattaaatttcggcaaattgaaaatgagccatgaattaatcaaaaattattcttgatcgttgttttgtaaaacataacttttttttgatttttttgggaggcgccctgtgttccacttattattatttgtttgttttgctatgtcaatactttaatataaaagaaattaatattggctgacatttcaatttaacgctaggcttatgttttgtttcgtgaataatctgcataaagaaaaaaagcagtatcgactctcctattgttgaatcactcttgcttcccttccattgtgcaggagatagtgacgcaatgaacattgattttaacgcttctagtcagttgggcccttgctcacaattgcctaaaatttgatcaattgtggattgaaaagttaatattctttcctgaattaacatgtctataggtaaggtattgagacggctcccatttaatttcttgtccgatctttataaaaaaatatttgttaaaggtacgtttaatttgaacatatagtgcagaagtcttcggaattttcatatagagttattttttagatactatatgcgacatattttcatagcccaaattaaattgttttataatttgtttggcttttacagattttaatttaacttaggtgtacgattcaatagaaagattttaacctgtaaaaatacgtgcaagttgtttggaaataccttttttgttggacaaagttgtatcaggtaagttgatagcatgtatcttaaatctggcgtggtgtttatgtattggcttcatcatgaaatagtttgttttgtccttttacttgtcaattttatttaaattacatgagggtacaattcattataacgtacttcggtgaaaggttaatgttaacaaatgccgtccggtcttctgcgtatctttcgatttcgtattaattatgcatagaaagggtttaagtgcaatgctatttctgatgatgtgtgtgtaacaccaacgacctgatgacgaaaaacttgactgtttattaaataaaatagcgcaacaagacggcggaatttataaatggcatgttgctcgtaaacagcagcgcgtgcctgttttaactctggagaaacaagttatattcaactatttgttatctcacaaagcacggtattgcactcttatctaatgtactaatacaaatatatatatatatatatatatatatatctagctgttgtcgaggtaagatgaactgcagttaacaataaaaaccaacttcgttatacacaaattcaatataaagcgacagctcagtgttgtagagtaggtacccaactttcttaatctgcaaaaggcaaatacatgatttataatatgtaactcagcataacaacctgtttttgccttttgcagattacatctgagggcgggttgtgtgggtcagaaatttatcaaaggaaaataaagttgattgaaagcaaatttgtttctactttattgttcatcatgGACGTTTGTAGAgcggccgcaaccATGGCGGATCTGCGAGTACCGGACATTCCGCTCGCCTCGTGTGATGATGATGATATCGATAGTAATAAGAATTTGAGCAACCATTCATCAGACGAGAAACATCACTGCAACAGCAACAGCGACGAGGAACGTCTTCATGACGAGTTCTCTGGATCCCTTGAGGACCTTGTCGGCAACTTTGACGAAAAAATTGCGGCATGCCTGAAGGACCACGAGGTGACGACAGCGGATATTGCACCTGTGCAGATACGTACTCAAGAGGAAGTTATGAATGAAAGCCAAACATGGTGGACATTAACCGGAAACTTTGGAAACATTCAACCTCTCGACTTTGGAACCTCTTCGATATGTAAAAAGATGGCCGCAGCTCTGGACAGTGATTCATTGAAAGACGACGCATCTACACGCCGAAGTATGACAAATTCCGATGATGAGGATCTTTTACGACAACAAATGGATGTTCATCAAATGATTGGACATCATCATGGATCTACGGATACTGGTGGTGAAACACCTCCACAGACTGCTGATCAAGTTATCGAAGAAATTGATGAAATGTTACAGGTACCGGTCGCCACCATGGTGAGCAAGGGCGAGGAGGATAACATGGCCATCATCAAGGAGTTCATGCGCTTCAAGGTGCACATGGAGGGCTCCGTGAACGGCCACGAGTTCGAGATCGAGGGCGAGGGCGAGGGCCGCCCCTACGAGGGCACCCAGACCGCCAAGCTGAAGGTGACCAAGGGTGGCCCCCTGCCCTTCGCCTGGGACATCCTGTCCCCTCAGTTCATGTACGGCTCCAAGGCCTACGTGAAGCACCCCGCCGACATCCCCGACTACTTGAAGCTGTCCTTCCCCGAGGGCTTCAAGTGGGAGCGCGTGATGAACTTCGAGGACGGCGGCGTGGTGACCGTGACCCAGGACTCCTCCCTGCAGGACGGCGAGTTCATCTACAAGGTGAAGCTGCGCGGCACCAACTTCCCCTCCGACGGCCCCGTAATGCAGAAGAAGACCATGGGCTGGGAGGCCTCCTCCGAGCGGATGTACCCCGAGGACGGCGCCCTGAAGGGCGAGATCAAGCAGAGGCTGAAGCTGAAGGACGGCGGCCACTACGACGCTGAGGTCAAGACCACCTACAAGGCCAAGAAGCCCGTGCAGCTGCCCGGCGCCTACAACGTCAACATCAAGTTGGACATCACCTCCCACAACGAGGACTACACCATCGTGGAACAGTACGAACGCGCCGAGGGCCGCCACTCCACCGGCGGCATGGACGAGCTGTACAAGTAAgaattc

*Nova[1b]-2011/+6>mScarlet*

Nova[1b]-2012/+6

mScarlet (Bindels et al. 2017)

ggcgcgccTCAAAAGCCCATACTGCTCTATATAGCGCTCTTTTGGTTTATAAATTAGGAAAACTCTGTCGTGCCAGTTTAGTAAATTGCGATTTTTTGGTTCACTGCGGTTGTCTTACACGGTTAATGTAACTAACCGTGTAATGTATTTCACGAAGAATTAAAAACAAGCAACGTTTTGACTTCTGTGTAGAGTGTGCTCTTCATTCGAAGTGATGTGTAGGTAGTTTTGTATCGATTATATGCTCTGGCTGGGCCATATGAAAGCCCAGCTCGATCTGCATGTTTGTCAACAAGTTACATAGCACAAACAACGCTAGACGGATGCACTTGACCGAACTTTAATACTGATTAGATAAGAAAAAAACGGCAAAATGTGCAATACTACATTGGGATCGATGCGTTTCGATTGTGACAATGAGCAAAATTCCGATAATTAAAAGTAGTTGATATTTTTTTAATACAAAACTATCGAATTAATTACAAAAGGTACCACATTCTTATACCAAAGCATTTAGATTTATTGATTTGAAGAATGTATGCGATCGAAACGAAGCAATTTCCTGATATAGTTAAAAGGTCATCAGAATAAATGGAAACTTAGTGACGTCGACCCCAATCATACGCTCGCAATTTTGGTCCATCGCTAATGCGCCTTTGGGAAATCATGGGACGACATCACGAGAGATTAGTTTCGAACTGCTTTGGTCTCTGCAGTGTTTCCAGACGGTTTCTATTTCGCACCCGGAAACACTGATTCGCCTTAGCGCGGCTTAGTGGGTTCGTCATTAAGAAATCGAAGTGAGATTGTTTACCAAACACATGCGCTTAGTTGTCTGTGGTTTCTGCCGTCGTATCATTAATTTGTATACATTTGGTAACAAATGCGAAATGACAGCGATTCGGACCGATTTTTTTCATCAAATTTTAACTGCGTGTTTCACATTATTTCTTAAAGCACGTCCGTGTTGCAATGCTTTGTAATGGATTATATTTGTGAGGCTGTAAATTACAAACCAGCGGCGGTACTTCAATTCCAGCACATGGTCGTAAAGAATTGTCCGAACATTACTGCGGGTAGCTCTGTACGTTTCGCCAATGATACTCTCAGTTAATGTTCAGCCATTAGTCGGGCATGCGATGCCAAAGTTTGCTCGGCAGTAAAATTGGATGCTTTGAAAAAAATCTAATAATTAACTGAAGCGGTTGTAGAACCGTCGTAAAAGTACCGCACTGAATGTAAAGGGGTGCAGAGGCTGACTACGCTAGACAAAGGTGCTGGTTTTACGACAAGAGTGCTTCCCGATTGCGATACGAAAAAAACAGTATTGTTGCGAGAGGGTGCGGTTAAAAAATGATTTTGAAACACTGTTTTCCGGAGTGCTGAGCTGTTCAAACGCAGCGTACAGCAACAATGCTTGTGCAGTGATCGAGTTGCTACGCGTTCTGTTTGATGGCTTCTATAAATAAAATGAAAGTTGTTGGTACCAGTCCGCCCAGAAGCGGTGCTTATTCGTTCGTAATAGGCGGTGCATTTCAGGAAGCAATACGTCGAGTGTTTTTCTTGCTTTTCTCCCAGAAATCTAAGAACAATAAATTGAATAAAGGACGGCGACTGCTAGGGCAAGATATCTGGCCAGATTCGAAAGCGCAGGTCCGCATCTAGGTTGCATCGTTGCCGGAAAGAGTGGGCTGAGTCTATAATGTGTATCCACGCGCGCAGTGATGTTGGAAGGCCCTGTTAGTATCAGCTCATTGTATGCAGCAGTGTTCTCGCCTGATTGACTGCGTTTGAGGTTAAAATTTAATGGCGTTGCGGTTTTCCTGTTTCTATGAACTGAGGTCGCAAGCAATTATAGATGTATTCGATCGATGAATATGAAAAGGGGCCGTATATTGTTGAGTGATACGTGTAAGGGTTAAAGCTTGTAGTTATATAAGTACAGAAGAGCCTGACGTATTGGAGGTTCATGATTTAATCATTAATCGCTCTTTTCATTCTGACAGAGAAAGTAGGATAATGCTAgcggccgcaaccATGGCTAGCGTGAGCAAGGGCGAGGCAGTGATCAAGGAGTTCATGCGGTTCAAGGTGCACATGGAGGGCTCCATGAACGGCCACGAGTTCGAGATCGAGGGCGAGGGCGAGGGCCGCCCCTACGAGGGCACCCAGACCGCCAAGCTGAAGGTGACCAAGGGTGGCCCCCTGCCCTTCTCCTGGGACATCCTGTCCCCTCAGTTCATGTACGGCTCCAGGGCCTTCACCAAGCACCCCGCCGACATCCCCGACTACTATAAGCAGTCCTTCCCCGAGGGCTTCAAGTGGGAGCGCGTGATGAACTTCGAGGACGGCGGCGCCGTGACCGTGACCCAGGACACCTCCCTGGAGGACGGCACCCTGATCTACAAGGTGAAGCTCCGCGGCACCAACTTCCCTCCTGACGGCCCCGTAATGCAGAAGAAGACAATGGGCTGGGAAGCGTCCACCGAGCGGTTGTACCCCGAGGACGGCGTGCTGAAGGGCGACATTAAGATGGCCCTGCGCCTGAAGGACGGCGGTCGCTACCTGGCGGACTTCAAGACCACCTACAAGGCCAAGAAGCCCGTGCAGATGCCCGGCGCCTACAACGTCGACCGCAAGTTGGACATCACCTCCCACAACGAGGACTACACCGTGGTGGAACAGTACGAACGCTCCGAGGGCCGCCACTCCACCGGCGGCATGGACGAGCTGTACAAGTAAgaattc

*Nova[1b]-2011/+6* driver (wild-type)

tcaaaagcccatactgctctatatagcgctcttttggtttataaattaggaaaactctgtcgtgccagtttagtaaattgcgattttttggttcactgcggttgtcttacacggttaatgtaactaaccgtgtaatgtatttcacgaagaattaaaaacaagcaacgttttgacttctgtgtagagtgtgctcttcattcgaagtgatgtgtaggtagttttgtatcgattatatgctctggctgggccatatgaaagcccagctcgatctgcatgtttgtcaacaagttacatagcacaaacaacgctagacggatgcacttgaccgaactttaatactgattagataagaaaaaaacggcaaaatgtgcaatactacattgggatcgatgcgtttcgattgtgacaatgagcaaaattccgataattaaaagtagttgatatttttttaatacaaaactatcgaattaattacaaaaggtaccacattcttataccaaagcatttagatttattgatttgaagaatgtatgcgatcgaaacgaagcaatttcctgatatagttaaaaggtcatcagaataaatggaaacttagtgacgtcgaccccaatcatacgctcgcaattttggtccatcgctaatgcgcctttgggaaatcatgggacgacatcacgagagattagtttcgaactgctttggtctctgcagtgtttccagacggtttctatttcgcacccggaaacactgattcgccttagcgcggcttagtgggttcgtcattaagaaatcgaagtgagattgtttaccaaacacatgcgcttagttgtctgtggtttctgccgtcgtatcattaatttgtatacatttggtaacaaatgcgaaatgacagcgattcggaccgatttttttcatcaaattttaactgcgtgtttcacattatttcttaaagcacgtccgtgttgcaatgctttgtaatggattatatttgtgaggctgtaaattacaaaccagcggcggtacttcaattccagcacatggtcgtaaagaattgtccgaacattactgcgggtagctctgtacgtttcgccaatgatactctcagttaatgttcagccattagtcgggcatgcgatgccaaagtttgctcggcagtaaaattggatgctttgaaaaaaatctaataattaactgaagcggttgtagaaccgtcgtaaaagtaccgcactgaatgtaaaggggtgcagaggctgactacgctagacaaaggtgctggttttacgacaagagtgcttcccgattgcgatacgaaaaaaacagtattgttgcgagagggtgcggttaaaaaatgattttgaaacactgttttccggagtgctgagctgttcaaacgcagcgtacagcaacaatgcttgtgcagtgatcgagttgctacgcgttctgtttgatggcttctataaataaaatgaaagttgttggtaccagtccgcccagaagcggtgcttattcgttcgtaataggcggtgcatttcaggaagcaatacgtcgagtgtttttcttgcttttctcccagaaatctaagaacaataaattgaataaaggacggcgactgctagggcaagatatctggccagattcgaaagcgcaggtccgcatctaggttgcatcgttgccggaaagagtgggctgagtctataatgtgtatccacgcgcgcagtgatgttggaaggccctgttagtatcagctcattgtatgcagcagtgttctcgcctgattgactgcgtttgaggttaaaatttaatggcgttgcggttttcctgtttctatgaactgaggtcgcaagcaattatagatgtattcgatcgatgaatatgaaaaggggccgtatattgttgagtgatacgtgtaagggttaaagcttgtagttatataagtacagaagagcctgacgtattggaggttcatgatttaatcattaatcgctcttttcattctgacagagaaagtaggataatgcta

*Nova[1b]-2011/+6 mEBF 1*

tcaaaagcccatactgctctatatagcgctcttttggtttataaattaggaaaactctgtcgtgccagtttagtaaattgcgattttttggttcactgcggttgtcttacacggttaatgtaactaaccgtgtaatgtatttcacgaagaattaaaaacaagcaacgttttgacttctgtgtagagtgtgctcttcattcgaagtgatgtgtaggtagttttgtatcgattatatgctctggctgggccatatgaaagcccagctcgatctgcatgtttgtcaacaagttacatagcacaaacaacgctagacggatgcacttgaccgaactttaatactgattagataagaaaaaaacggcaaaatgtgcaatactacattgggatcgatgcgtttcgattgtgacaatgagcaaaattccgataattaaaagtagttgatatttttttaatacaaaactatcgaattaattacaaaaggtaccacattcttataccaaagcatttagatttattgatttgaagaatgtatgcgatcgaaacgaagcaatttcctgatatagttaaaaggtcatcagaataaatggaaacttagtgacgtcgaccccaatcatacgctcgcaattttggtccatcgctaatgcgcctttAAgaaatcatgggacgacatcacgagagattagtttcgaactgctttggtctctgcagtgtttccagacggtttctatttcgcacccggaaacactgattcgccttagcgcggcttagtgggttcgtcattaagaaatcgaagtgagattgtttaccaaacacatgcgcttagttgtctgtggtttctgccgtcgtatcattaatttgtatacatttggtaacaaatgcgaaatgacagcgattcggaccgatttttttcatcaaattttaactgcgtgtttcacattatttcttaaagcacgtccgtgttgcaatgctttgtaatggattatatttgtgaggctgtaaattacaaaccagcggcggtacttcaattccagcacatggtcgtaaagaattgtccgaacattactgcgggtagctctgtacgtttcgccaatgatactctcagttaatgttcagccattagtcgggcatgcgatgccaaagtttgctcggcagtaaaattggatgctttgaaaaaaatctaataattaactgaagcggttgtagaaccgtcgtaaaagtaccgcactgaatgtaaaggggtgcagaggctgactacgctagacaaaggtgctggttttacgacaagagtgcttcccgattgcgatacgaaaaaaacagtattgttgcgagagggtgcggttaaaaaatgattttgaaacactgttttccggagtgctgagctgttcaaacgcagcgtacagcaacaatgcttgtgcagtgatcgagttgctacgcgttctgtttgatggcttctataaataaaatgaaagttgttggtaccagtccgcccagaagcggtgcttattcgttcgtaataggcggtgcatttcaggaagcaatacgtcgagtgtttttcttgcttttctcccagaaatctaagaacaataaattgaataaaggacggcgactgctagggcaagatatctggccagattcgaaagcgcaggtccgcatctaggttgcatcgttgccggaaagagtgggctgagtctataatgtgtatccacgcgcgcagtgatgttggaaggccctgttagtatcagctcattgtatgcagcagtgttctcgcctgattgactgcgtttgaggttaaaatttaatggcgttgcggttttcctgtttctatgaactgaggtcgcaagcaattatagatgtattcgatcgatgaatatgaaaaggggccgtatattgttgagtgatacgtgtaagggttaaagcttgtagttatataagtacagaagagcctgacgtattggaggttcatgatttaatcattaatcgctcttttcattctgacagagaaagtaggataatgcta

*Nova[1b]-2011/+6 mEBF 2*

tcaaaagcccatactgctctatatagcgctcttttggtttataaattaggaaaactctgtcgtgccagtttagtaaattgcgattttttggttcactgcggttgtcttacacggttaatgtaactaaccgtgtaatgtatttcacgaagaattaaaaacaagcaacgttttgacttctgtgtagagtgtgctcttcattcgaagtgatgtgtaggtagttttgtatcgattatatgctctggctgggccatatgaaagcccagctcgatctgcatgtttgtcaacaagttacatagcacaaacaacgctagacggatgcacttgaccgaactttaatactgattagataagaaaaaaacggcaaaatgtgcaatactacattgggatcgatgcgtttcgattgtgacaatgagcaaaattccgataattaaaagtagttgatatttttttaatacaaaactatcgaattaattacaaaaggtaccacattcttataccaaagcatttagatttattgatttgaagaatgtatgcgatcgaaacgaagcaatttcctgatatagttaaaaggtcatcagaataaatggaaacttagtgacgtcgaccccaatcatacgctcgcaattttggtccatcgctaatgcgcctttgggaaatcatAAgacgacatcacgagagattagtttcgaactgctttggtctctgcagtgtttccagacggtttctatttcgcacccggaaacactgattcgccttagcgcggcttagtgggttcgtcattaagaaatcgaagtgagattgtttaccaaacacatgcgcttagttgtctgtggtttctgccgtcgtatcattaatttgtatacatttggtaacaaatgcgaaatgacagcgattcggaccgatttttttcatcaaattttaactgcgtgtttcacattatttcttaaagcacgtccgtgttgcaatgctttgtaatggattatatttgtgaggctgtaaattacaaaccagcggcggtacttcaattccagcacatggtcgtaaagaattgtccgaacattactgcgggtagctctgtacgtttcgccaatgatactctcagttaatgttcagccattagtcgggcatgcgatgccaaagtttgctcggcagtaaaattggatgctttgaaaaaaatctaataattaactgaagcggttgtagaaccgtcgtaaaagtaccgcactgaatgtaaaggggtgcagaggctgactacgctagacaaaggtgctggttttacgacaagagtgcttcccgattgcgatacgaaaaaaacagtattgttgcgagagggtgcggttaaaaaatgattttgaaacactgttttccggagtgctgagctgttcaaacgcagcgtacagcaacaatgcttgtgcagtgatcgagttgctacgcgttctgtttgatggcttctataaataaaatgaaagttgttggtaccagtccgcccagaagcggtgcttattcgttcgtaataggcggtgcatttcaggaagcaatacgtcgagtgtttttcttgcttttctcccagaaatctaagaacaataaattgaataaaggacggcgactgctagggcaagatatctggccagattcgaaagcgcaggtccgcatctaggttgcatcgttgccggaaagagtgggctgagtctataatgtgtatccacgcgcgcagtgatgttggaaggccctgttagtatcagctcattgtatgcagcagtgttctcgcctgattgactgcgtttgaggttaaaatttaatggcgttgcggttttcctgtttctatgaactgaggtcgcaagcaattatagatgtattcgatcgatgaatatgaaaaggggccgtatattgttgagtgatacgtgtaagggttaaagcttgtagttatataagtacagaagagcctgacgtattggaggttcatgatttaatcattaatcgctcttttcattctgacagagaaagtaggataatgcta

*Nova[1b]-2011/+6 mEBF 1+2*

tcaaaagcccatactgctctatatagcgctcttttggtttataaattaggaaaactctgtcgtgccagtttagtaaattgcgattttttggttcactgcggttgtcttacacggttaatgtaactaaccgtgtaatgtatttcacgaagaattaaaaacaagcaacgttttgacttctgtgtagagtgtgctcttcattcgaagtgatgtgtaggtagttttgtatcgattatatgctctggctgggccatatgaaagcccagctcgatctgcatgtttgtcaacaagttacatagcacaaacaacgctagacggatgcacttgaccgaactttaatactgattagataagaaaaaaacggcaaaatgtgcaatactacattgggatcgatgcgtttcgattgtgacaatgagcaaaattccgataattaaaagtagttgatatttttttaatacaaaactatcgaattaattacaaaaggtaccacattcttataccaaagcatttagatttattgatttgaagaatgtatgcgatcgaaacgaagcaatttcctgatatagttaaaaggtcatcagaataaatggaaacttagtgacgtcgaccccaatcatacgctcgcaattttggtccatcgctaatgcgcctttAAgaaatcatAAgacgacatcacgagagattagtttcgaactgctttggtctctgcagtgtttccagacggtttctatttcgcacccggaaacactgattcgccttagcgcggcttagtgggttcgtcattaagaaatcgaagtgagattgtttaccaaacacatgcgcttagttgtctgtggtttctgccgtcgtatcattaatttgtatacatttggtaacaaatgcgaaatgacagcgattcggaccgatttttttcatcaaattttaactgcgtgtttcacattatttcttaaagcacgtccgtgttgcaatgctttgtaatggattatatttgtgaggctgtaaattacaaaccagcggcggtacttcaattccagcacatggtcgtaaagaattgtccgaacattactgcgggtagctctgtacgtttcgccaatgatactctcagttaatgttcagccattagtcgggcatgcgatgccaaagtttgctcggcagtaaaattggatgctttgaaaaaaatctaataattaactgaagcggttgtagaaccgtcgtaaaagtaccgcactgaatgtaaaggggtgcagaggctgactacgctagacaaaggtgctggttttacgacaagagtgcttcccgattgcgatacgaaaaaaacagtattgttgcgagagggtgcggttaaaaaatgattttgaaacactgttttccggagtgctgagctgttcaaacgcagcgtacagcaacaatgcttgtgcagtgatcgagttgctacgcgttctgtttgatggcttctataaataaaatgaaagttgttggtaccagtccgcccagaagcggtgcttattcgttcgtaataggcggtgcatttcaggaagcaatacgtcgagtgtttttcttgcttttctcccagaaatctaagaacaataaattgaataaaggacggcgactgctagggcaagatatctggccagattcgaaagcgcaggtccgcatctaggttgcatcgttgccggaaagagtgggctgagtctataatgtgtatccacgcgcgcagtgatgttggaaggccctgttagtatcagctcattgtatgcagcagtgttctcgcctgattgactgcgtttgaggttaaaatttaatggcgttgcggttttcctgtttctatgaactgaggtcgcaagcaattatagatgtattcgatcgatgaatatgaaaaggggccgtatattgttgagtgatacgtgtaagggttaaagcttgtagttatataagtacagaagagcctgacgtattggaggttcatgatttaatcattaatcgctcttttcattctgacagagaaagtaggataatgcta

*Nova[1a]-922/-1*

TGCTGGTTAAGTGTTGCTGCAAAAATGGCATTGGTTTGTGGATGTTCTTGTATACAGACTGTTAATGTTAATAAAATTGCACTTTTATTCGGAAATATTACGATTTTGGTCAACTAGCAAGATGAAGGAAATCCAGGTGCAGGTTAACGATTTTGTTTGCACGTTTTTTTAGGGTTTTATTTGCAAAAAAAGACAGTTTTTGTCGTTTTTTGACATTTTGCCAAGAGTTTTTGTTGATCCTGGCAGTTTATATTCTGTCATACACTGACATTTAGTGTACCGCTTGTCACCTCAGATTTTGCTACAAATTTAAAACAACGTGACGCCTATGAGTAATTGATGTCGCATTTTGTAACTTTTATGACACAAGAGTCGCCACAGTGTGTCGCTAGAGATGAAATACTAATCTCACGCTGGCGATGACGTCACGGCTGTTAACAATACGTTGGCGGGTATAGCTATATGCATATTATTTACATGCATATATCTATACCAATCATTAGTGGTAAATAAATGTTATACGGCTTTTTTATTATTGCAAATGGTATTAATGTGGTATATAGTAATTTATTTAATGCAGTATATTGTAATTGTCGGCACCGAAAAAAAGCAACGCCATATACTTAAACACTAAGTAGGAAGCCTCGAAATAAGTTGATCACGTGCATACGCATGCCGCAGTTTATTTATGTCACGATCTTAAGCTATTCGGTAAAGCTTATTGGGCGGGCGTAGGCAATTCAGCCATACGGGTAAATAGGTTTTCCGAACTGCCATAGAGCGGCGCGCGTACACTGTTGTCTGCGCGTTCATGAAGAGTCGAGGGAAAGAACGCCGGAGAAAAAAATTCGCTGCACTGTCAGTGTTCGAGAGCCAGCAAGCTAGTTCGCCAGAAGTGATCGCATTGTGACAGAATGCGCCG

**Electroporation mixes (per 700 µl of solution):**

Testing sgRNA combinations for Agrin CRISPR by RT-PCR (Fig5B)

40 µg Eef1a>Cas9

60 µl bead-purified U6>Agrin sgRNA OSO-PCR products (10 µl each sgRNA)

Control sgRNA to compare to Agrin CRISPR by RT-PCR (Fig5B)

40 µg Eef1a>Cas9

50 µl bead-purified U6>Control sgRNA OSO-PCR product

Neural-specific Z+ Agrin CRISPR to see AChRA1::GFP clustering (Fig 5C)

40 µg Sox1/2/3>Cas9

60 µl bead-purified U6>Agrin sgRNA OSO-PCR products (10 µl each sgRNA)

40 µg Vacht -2083/+15>Unc-76::mCherry

20 µg Tbx6-r.b>AChRA1::GFP

Neural-specific control CRISPR to compare to Z+ Agrin CRISPR (Fig5C)

40 µg Sox1/2/3>Cas9

50 µl bead-purified U6>Control sgRNA OSO-PCR product

40 µg Vacht -2083/+15>Unc-76::mCherry

20 µg Tbx6-r.b>AChRA1::GFP

Neural-specific control CRISPR to compare to Agrin CRISPR 1+3 (Fig5D)

40 µg Sox1/2/3>Cas9

50 µg U6>Control

40 µg Vacht -4315/+15>Unc-76::mCherry

20 µg Tbx6-r.b>AChRA1::GFP

Neural-specific Z+ Agrin CRISPR sgRNAs 1+3 (Fig5D)

40 µg Sox1/2/3>Cas9

25 µg U6>Agrin.1

25 µg U6>Agrin.3

40 µg Vacht -4315/+15>Unc-76::mCherry

20 µg Tbx6-r.b>AChRA1::GFP

Neural-specific control CRISPR to compare to Agrin CRISPR 5+8 (Fig3D)

40 µg Sox1/2/3>Cas9

50 µg U6>Control

40 µg Vacht -4315/+15>Unc-76::mCherry

20 µg Tbx6-r.b>AChRA1::GFP

Neural-specific Z+ Agrin CRISPR sgRNAs 5+8 (Fig3D)

40 µg Sox1/2/3>Cas9

25 µg U6>Agrin.5

25 µg U6>Agrin.8

40 µg Vacht -4315/+15>Unc-76::mCherry

20 µg Tbx6-r.b>AChRA1::GFP

Muscle-specific Lrp4 CRISPR (Fig3E,F)

35 µg Tbx6-r.b>Cas9

60 µg Tbx6-r.b>CD4::mCherry

25 µg U6>Lrp4.2

25 µg U6>Lrp4.4

20 µg Tbx6-r.b>AChRA1::GFP

Muscle-specific Nova (negative control) CRISPR (Fig3E,F)

35 µg Tbx6-r.b>Cas9

60 µg Tbx6-r.b>CD4::mCherry

50 µg U6>Nova2.3

20 µg Tbx6-r.b>AChRA1::GFP

Nova CRISPR for RT-PCR, sgRNA 1.2(Fig6B)

40 µg Sox1/2/3>Cas9

40 µg U6>Nova1.2

Nova CRISPR for RT-PCR, sgRNA 2.1(Fig6B)

40 µg Sox1/2/3>Cas9

40 µg U6>Nova2.1

Nova CRISPR for RT-PCR, sgRNA 2.3(Fig6B)

40 µg Sox1/2/3>Cas9

40 µg U6>Nova2.3

Control CRISPR for RT-PCR, sgRNA 1.2(Fig6B)

40 µg Sox1/2/3>Cas9

40 µg U6>Control

Nova sgRNA “Mix” CRISPR for RT-PCR (Fig6B,C)

25 µg Ef1a>Cas9

25 µg U6>Nova1.2

25 µg U6>Nova2.1

25 µg U6>Nova2.3

Control CRISPR for RT-PCR (Fig6C)

25 µg Ef1a>Cas9

75 µg U6>Control

Eef1a>Nova(MLN) for RT-PCR (Fig6C)

20 µg Eef1a>Nova(MLN)

Eef1a>H2B::CFP as control for Nova(MLN) overexpression RT-PCR (Fig6C)

20 µg Eef1a>H2B:CFP

Neural-specific Nova CRISPR to compare to rescue (Fig6E)

40 µg Sox1/2/3>Cas9

25 µg U6>Nova1.2

25 µg U6>Nova2.1

35 µg Islet -7216/-3950 + bpFOG>Unc-76::mCherry

20 µg Tbx6-r.b>AChRA1::GFP

Neural-specific control CRISPR to compare to rescue (Fig4E)

40 µg Sox1/2/3>Cas9

50 µg U6>Control

35 µg Islet -7216/-3950 + bpFOG>Unc-76::mCherry

20 µg Tbx6-r.b>AChRA1::GFP

Rescue of neural-specific Nova CRISPR (Fig4E)

40 µg Sox1/2/3>Cas9

25 µg U6>Nova1.2

25 µg U6>Nova2.1

35 µg Islet -7216/-3950 + bpFOG>Unc-76::mCherry

20 µg Tbx6-r.b>AChRA1::GFP

40 µg Islet -7216/-3950 + bpFOG>Nova(MLN) rescue

Neural-specific Nova CRISPR for cluster density (Fig4D,F)

40 µg Sox1/2/3>Cas9

15 µg U6>Nova1.2

15 µg U6>Nova2.1

15 µg U6>Nova2.3

40 µg Vacht -2083/+15>Unc-76::mCherry

20 µg Tbx6-r.b>AChRA1::GFP

Neural-specific control CRISPR for cluster density (Fig4D,F)

40 µg Sox1/2/3>Cas9

45 µg U6>Control

40 µg Vacht -2083/+15>Unc-76::mCherry

20 µg Tbx6-r.b>AChRA1::GFP

Neural-specific Ebf CRISPR to assay Nova>GFP expression (Fig7C,D)

35 µg Sox1/2/3>Cas9

40 µg U6>Ebf.C

60 µg Nova[1b] -2011/+6>GFP

45 µg Islet -7216/-3950 + bpFOG>tagRFP

Neural-specific Control CRISPR to assay Nova>GFP expression (Fig7C,D)

35 µg Sox1/2/3>Cas9

40 µg U6>DenhT2

60 µg Nova[1b] -2011/+6>GFP

45 µg Islet -7216/-3950 + bpFOG>tagRFP

WT vs WT (Fig7F-H)

60 µg Nova[1b] -2011/+6>GFP

60 µg Nova[1b] -2011/+6>mScarlet

WT vs mEbf1 (Fig7F,G)

60 µg Nova[1b] -2011/+6 mEbf1>GFP

60 µg Nova[1b] -2011/+6>mScarlet

WT vs mEbf2 (Fig7F,G)

60 µg Nova[1b] -2011/+6 mEbf2 >GFP

60 µg Nova[1b] -2011/+6>mScarlet

WT vs mEbf1+2 (Fig7G)

60 µg Nova[1b] -2011/+6 mEbf1+2>GFP

60 µg Nova[1b] -2011/+6>mScarlet
