# Supplementary material for: Neuron-specific Agrin splicing by Nova RNA-binding proteins regulates conserved neuromuscular junction development in chordates
Source: PLoS Biol. 2025 Sep 12;23(9):e3003392. doi: 10.1371/journal.pbio.3003392 (PMC12445529; doi:10.1371/journal.pbio.3003392)
Supplement: Attachment — Submitted filename: 152789_1_rebuttal_3441632_sz6swq.pdf [file pbio.3003392.s019.pdf]

We thank the reviewers for their helpful comments and suggestions. Below you may find the point-by-point replies to their concerns.

Reviewer #1

"The research is meticulously conducted, and the data are compelling, as they demonstrate that the Nova-agrin-Lrp4-MuSK axis is also operational in non-vertebrates. The conclusions drawn by the authors are generally adequate; however, I find some instances of "it is the first time..." to be unnecessary."

We have removed all unnecessary claims to that effect.

"The work also presents an unexpected finding that mouse Nova protein is unable to splice the Ciona agrin mini-gene (Figure 3). I believe the inability of mouse Nova1 and Nova2 to splice the Ciona agrin could also be due to insufficient expression levels of the mouse proteins. Therefore, the authors should include either a positive control (e.g., mouse agrin mini-gene) or demonstrate that the proteins are expressed at comparable levels."

We have now included two additional datasets supporting our conclusion. First, we have included the positive control with the mouse *Agrin* minigene as suggested by the reviewer, which shows that mouse Nova1 and Nova2 are indeed still able to splice the mouse *Agrin* minigene in our assay (Figure 3C). Second, we included fluorescence images of the GFP-fused mouse Nova1 and Nova2 showing their proper expression in the cells (Figure S7).

"I am also not fully convinced that the model of autoinhibition for Ciona Nova is supported by sufficient experimental data. Again, there are no data showing that the levels of the various deletion mutants of Nova are consistent and hence, there could be issues with the stbsility of some of the deletion mutants and this could explain the observed difference in activity."

We have added a few more datasets to further investigate the model. First, we have added an independent biological replicate of the "MLN" Nova isoform deletion mutant assays (Figure S8), as well as a separate assay using deletion mutants based on the "MMM" isoform (Figure S9). The results were consistent in both cases, confirming our initial observations. Next, we tested more directly the idea proposed by the reviewer that there are issues with stability, by looking at the fluorescence of the GFP-fused mutants. We did notice that the N/C-terminal deletion mutants were not expressed as well, but this was always mitigated by concurrent deletion of the KH3 domain. We have now expanded our discussion in the text to propose that there may be a negative effect of the KH3 domain on Nova expression/stability in the absence of the N/C termini. Although different from the model in which KH3 directly inhibits KH1/KH2, there does seem to be some inhibitory effect of KH3 on Nova expression/stability.

"- In all schematic presentations, exon Z6 appears larger than exon Z5. However, Z6 is only 24 bp long, while Z5 is 3434 bp long. Please adjust this representation."

To clarify, in *Ciona* Z6 is 18 bp long, and Z5 is 15 bp long, hence they code for 6 and 5 amino-acids, respectively. This is different from the mammalian Z exons, which may be the source of the confusion here. In our schematics, we are only representing the *Ciona* Z exons.

"- Is there consistency in the relative proportions of the 24-bp (Z6), 33-bp (Z5), and 57-bp

(Z6 + Z5) PCR products? Studies in vertebrates have shown that AChR clustering activity is highest with the Z8 and Z19 products, while the Z11 product appears to be somewhat less active. It would be nice to also point out the different splice products are detected in Ciona."

It was not clear if there was any consistency in the relative proportions of *Ciona Agrin* splice products in the minigene assays as performed in cultured mammalian cells, though in Figure 1 we have pointed out a more detailed characterization of the different splice products *in vivo* in *Ciona.* The different splice products' confirmed sequences are also shown in the supplemental sequences file.

"Line 111: 'Z11' Agrin should be corrected to 'Z19' Agrin."

To clarify again, we are only referring to the *Ciona Agrin* Z exons, which are not the same sizes as the mammalian Z exons. While Z19 would refer to the combination of exons Z8 and Z11 (8+11 = 19) in mammals, here in *Ciona* the equivalent combination is Z11 (Z5 + Z6).

"Line 168: "Figure H" should be updated to "Figure 2H.""

Fixed.

Reviewer #2:

"44 - ALS, references 8-12. These are old papers. A new review should be cited, either instead of in addition."

We have read some newer reviews and cite three more recent reviews (references 10-12) now.

56 - "many" cases of CMS - some are not due to mutations in this pathway

We have altered this to say "many".

57 - refs 29-46. This is a very large number of references for a point this is quite unimportant to the story. It would be better to cite recent reviews.

We have removed some references and also cited more recent reviews here (references 38, 39).

168 - should be 2H

Fixed.

205 - make N terminal extension more apparent in Figure 3D

We have recolored the N terminus to be red, as to make it more apparent, in figures 3 and S8 and S9.

235 - not a complete sentence

Fixed.

308+ - can the authors clarify whether EBF knockdown has a selective effect on Nova vs general failure of the neurons to acquire a MN phenotype

We have been investigating this in a separate study on MN specification and differentiation in *Ciona*, which will be published as a preprint soon. EBF does not have a selective effect on *Nova* expression, as it appears to be regulating multiple aspects of neuronal differentiation, consistent with its role as previously studied in *Ciona* and other organisms (e.g. Kratsios et al. 2012, Catela et al. 2019, etc).

614 - explain in figure legend the decrease in apparent MR from left to right in 4B

This is just an example of “bowed” or “curved” bands frequently seen in electrophoresis, usually due to uneven heat dissipation or other electrophoresis issues. However, the bands all correspond to the same products (Z+). We added an explanation in the legend.

General - three other key components of the pathway are MuSK, rapsyn, and DOK-7. Functional studies of these genes fall beyond the scope of this paper, but it would be helpful to know whether they are expressed in muscle and, if so, whether expression is muscle-specific.

We have added this to the discussion. While Musk and Dok-7 remain unstudied in *Ciona*, it has been shown that Rapsyn is muscle-specific in *Ciona* (Nishino et al. 2011).

Reviewer #3:

“1) The authors report two main Nova isoforms that seem to be produced by alternative promoters. They also claim that the MLN isoform is more strongly expressed in two of the studied conditions compared to the MMM protein (eggs and heart in Fig 1G), while both are equally abundant in st. 22.5 embryos and brain (Fig S1 and line 130). Therefore, both isoforms are likely involved in the regulation of the Agrin AS event. When performing the experiments that require to express the Nova protein, the authors choose to work with the "MLN" isoform arguing that it is more "ubiquitous" than the "MMM" isoform, although the last has a more evident nuclear localization signal (NLS) sequence. In the minigene analysis, the MLN isoform fails to produce transcripts with Z6 exon (which seems to be the most common Z+ isoform in the brain), and the amount of Z11-containing transcripts is very low compared to st. 22.5. Given that the N-terminal domain has a regulatory influence, as demonstrated by the authors, and that the MMM isoform is potentially more "neural-restricted" than the MLN, an intriguing possibility is that the MMM isoform might enhance the inclusion of Z6 and Z11 isoforms. To solve this issue, I suggest two experiments:

- Perform the minigene assay with the MMM isoform of Nova and the wild type version of the minigene to check the level of inclusion of Z6 and Z6+Z5 (Z11) exons.”

We have added additional minigene assay data using the MMM isoform (S9). We did not detect Z6 isoforms with MMM, though there may be slight differences in the ratio of Z5 and Z11 compared to the MLN assay. We believe this indicates that nuclear localization is not rate-limiting in our heterologous mammalian cell minigene assay, although it very well may regulate splicing activity more meaningfully *in vivo* in *Ciona.* This may be especially true in post-mitotic

cells, as opposed to during embryogenesis when actively proliferating cells will break down and then reconstitute their nuclear envelopes over and over again, thus potentially allowing some of the MLN isoform to find its way into the nucleus. We still believe the production of the Z6 isoform may depend on additional *Ciona*-specific factors missing from the mammalian cells in our heterologous assay.

"- Test the regulatory activity of the upstream genomic region of exon 1a, in an equivalent way as for exon 1b in Fig 7A and B, to explore whether the promoter of the MMM isoform has a neural-restricted expression that could explain the AS pattern observed in st. 22.5 and brain."

We have done this, shown in Figure S15, which revealed that the promoter upstream of exon 1a (encoding the MMM isoform) drives only expression in mesenchyme and some epidermal cells, with no neuronal expression visible. This suggests that the majority of the neural expression is due to the *cis*-regulatory elements in the region between exons 1a and 1b. However, this region does not necessarily activate transcription only at exon 1b (encoding MLN isoform), as intronic elements can loop back and regulate transcription off "upstream" promoters. Thus we propose that the *Nova [1b] -2011/+6* region drives expression of both MLN and MMM isoforms, though this remains to be fully tested. We believe the regulation and function of the different Nova isoforms in *Ciona* is beyond the scope of the current paper, though we are interested in investigating this more thoroughly in follow-up studies.

"2) The authors unveil the conservation of an Agrin AS event between mammals and a tunicate species with similar functional consequences for AChR clustering. While this is absolutely correct, the relatively low similarity of the AS exons between Ciona and mammals shown in Fig 1A may raise confusion or doubts in the readers regarding the homology of the event (as it did in my own case before I checked it in more detail). Therefore, an explicit alignment of both constitutive and alternative exons in a supplementary figure to clearly demonstrate the homology of the AS event across major taxonomic groups (with a few vertebrate and tunicate species) might help.

Furthermore, expression of Nova in motor neurons of amphioxus (Branchiostoma lanceolatum) was previously reported (ref. 60), and a quick look into publicly available Agrin transcripts (https://www.ncbi.nlm.nih.gov/gene/136443694) reveals a homologous AS event in this cephalochordate species.

C1 "Z7/Z6/Z8" C2 (partial)

Bla QADPAPLRQEGVG--LDGTTILNYPNAINK ... E-SNSIRE ...
QEPNQDDNHFEVTFRTTSDHGLLLWNHKPGGG-DFIALAI
Cro HSTDLLQDEQATAIYLDGTTKIMYRNAVKA ... --PNDFRE ... SRART-
HNNYEIVFRTTARHGLLLMVGKAREGVDYIALAI
Mmu IVEKSVGDLETLA--FDGRTYIEYLNAVTE ... ELTNEIPA ... EKALQ-
SNHFELSLRTEATQGLVLWIGKVGERADYMALAI
: . :** * : * **:. . *.: ... *.:*: :** : :**:* * *.:****

These two facts suggests a potential origin of the Nova-Agrin regulation at the base of the chordate phylum (and not restricted to Olfactores), which could be mentioned in the discussion as a relevant possibility."

We thank the reviewer for this suggestion. Indeed, we have now added a more detailed alignment with Agrin sequences from more species in Figures S2 and S3, including amphioxus as so helpfully identified by the reviewer. We have added the observation that amphioxus *Agrin* appears to have a single Z exon encoding the NxI/V motif (no evidence for two Z exons as in tunicates or vertebrates). This indeed suggests that this pathway may be a chordate innovation, as we now discuss. We also add AlphaFold-assisted predictions of the NxF motif binding to the equivalent pocket in Lrp4 in both *Ciona* and mammals (Figure S1).

Line 168: Figure 2H instead of Figure H.

Fixed.

Line 287: "Taken together, these results reveal that a Nova-Agrin-Lrp4 pathway for AChR receptor clustering at the neuromuscular synapse is conserved from mammals to tunicates." While this sentence might be true, from mammals to tunicates might imply that it is conserved in all vertebrate and tunicate lineages, and this is not explored in the manuscript (there might be secondary losses). It would be more technically correct to say something similar like "...the neuromuscular synapse is conserved in the studied mammalian and tunicate lineages" or "...the neuromuscular synapse originated before the evolutionary divergence of tunicates and vertebrates"

We have fixed this now in a few places.

"Line 342. At the end of this paragraph, the possibility of conservation of the mechanism also in amphioxus could be discussed."

We now discuss the amphioxus sequence and the idea that this mechanism was present in the last common chordate ancestor.

"Line 383: "the the apparent"."

Fixed.

"I agree that the mouse-specific agrin minigene to test the functionality of Nova1 and Nova2 would be a suitable positive control to discard protein stability/expression issues."

We have tested this now with GFP fusion images (Figure S7) and using the mouse *Agrin* minigene (Figure 3C). Both indicate proper expression/splicing activity of mouse Nova1 and Nova2, supporting the idea that there is still some type of cross-species incompatibility as tested in mammalian cells.

"The only minor limitation, in my opinion, is that it lacks testing of the MMM Nova isoform in the minigene assay, to explore whether it has (or not) a complementary function to the MLN isoform that could fully explain the endogenous AS pattern."

We have added MMM minigene assays, and these were largely identical to MLN assays. We propose that the N-terminus and nuclear localization do not significantly impact activity of *Ciona* Nova as tested in mammalian cells, however we cannot exclude the possibility that things may be different *in vivo* in *Ciona.*
